# Chiral Molecular Propellers of Triarylborane Ammonia Adducts

**DOI:** 10.1002/anie.202014130

**Published:** 2020-12-10

**Authors:** Michael Kemper, Elric Engelage, Christian Merten

**Affiliations:** ^1^ Ruhr Universität Bochum Fakultät für Chemie und Biochemie, Organische Chemie II Universitätsstrasse 150 44801 Bochum Germany

**Keywords:** chirality, circular dichroism, molecular propeller, stereochemistry, vibrational spectroscopy

## Abstract

Chiral molecular propeller conformations have been induced to various triaryl structures including trityl derivatives and triaryl boranes. For borane–amine adducts, such induced propeller chirality has not been reported yet due to the low energy barrier for racemization in common triarylboranes such as B(C_6_H_5_)_3_ or B(C_6_F_5_)_3_. Herein, we demonstrate that point chirality in side chains of chiral triarylborane–ammonia adducts, which feature intramolecular hydrogen bonds in addition to the dative N→B bond, can efficiently be transferred to triarylborane propeller chirality. Employing X‐ray crystallography and ECD/VCD spectroscopy for structural characterizations, we investigate three examples with different steric demands of the incorporated chiral alkoxy side groups. We elucidate the conformational preferences of the molecular propellers. Furthermore, we show that computationally predicted conformational preferences obtained for the isolated, only implicitly solvated molecules are actually opposite to the experimentally observed ones.

## Graphical abstract

Using VCD spectroscopy as key analytical tool, it was found that point chirality in side chains of chiral triarylborane–ammonia adducts, which feature intramolecular hydrogen bonds in addition to the dative N→B bond, can efficiently be transferred to triarylborane propeller chirality.

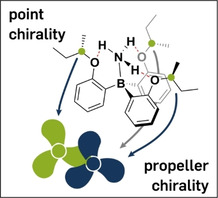

Molecular propeller conformations have attracted significant interest since Mislow's seminal papers on conformational preferences of triaryl compounds^[1]^ and the preparation of enantiopure propeller molecules with different pivot atoms.^[2]^ The chirality of trityl propellers has been utilized for chiral recognition studies, for instance using poly(trityl methacrylate) stationary phases, in which the helical chirality of the polymer backbone is stabilized by interlocked trityl groups, which themselves adopt a preferential propeller conformation.^[3]^ Recent studies on the dynamic stereochemistry^[4]^ of trityl ethers showed that point chirality of alcohols can successfully be transmitted to molecular propellers.^[5]^

As one key example, Mislow pointed out the propeller‐shape of triaryl boranes. Although triaryl borane indeed possesses enantiomeric propeller conformations in solid state,^[6]^ isolation in solution is prevented by rapid exchange between the two chiral forms. Only recently, Ito and co‐workers showed that propeller‐shaped triaryl boranes can be realized by introducing large 1,3‐diethynylphenylenes as aryl substituents, which effectively interlock the blades and thus prevent racemization.^[7]^

In triarylborane–nitrogen adducts, the tetrahedral borane also adopts a chiral conformation as confirmed by crystallography and NMR for neutral and anionic adducts of B(C_6_F_5_)_3_ with N‐heterocycles.^[8]^ It must be noted, however, that the triarylborane does not adopt a true propeller conformation, but a two‐bladed propeller‐like structure with one phenyl ring eclipsing the B−N bond. Such conformations are not uncommon and they can also often be observed for sterically unhindered trityl compounds.^[5a]^ Similar to boranes, isolation of enantiopure B−N adducts with predominant handedness of the borane propeller are prevented by low racemization barriers.

Trauner et al. introduced a class of borane–ammonia adducts in which the ammonia is tightly bound through a dative N→B bond and additional three intramolecular hydrogen bonds (**1 a**, Scheme 1).^[9]^ As evident from crystal structure analysis, this four‐point interaction forces the borane to adopt a true propeller shaped conformation. The additional interactions can be expected to raise the energy barrier for propeller inversion compared to boranes and borane–amine adducts. Based on the core structure **1**, we targeted chiral derivatives **1 b**–**d** (Scheme 1) expecting an efficient transfer of stereochemical information from the point chirality in the alkoxy side groups to the propeller structure of the adducts, which should lead to a significant preference of one of the two possible propeller conformations over the other.

**Scheme 1:**
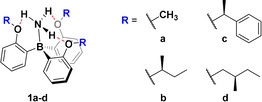
Structures of the borane ammonia complexes **1 a**–**d**.

We prepared the borane amines **1 b**–**d**, which possess alkoxy side groups with different steric demand, following a slightly modified synthetic route reported by Trauner et al.^[9]^ In brief, reaction of *ortho*‐bromo phenol **2** with alcohols **3 b**–**d** under Mitsunobu conditions leads to the phenyl ethers **4 b**–**d**. In a subsequent one‐pot reaction, *ortho*‐lithiation with *n*‐butyl lithium or, alternatively, formation of the corresponding Grignard reagent, and reaction with trifluoroborane etherate led to the in situ formation of the intermediate **5 b**–**d**. The target compounds **1 b**–**d** were obtained as white solids by complexation through quenching with a concentrated aqueous solution of ammonia (Scheme 2).

**Scheme 2:**
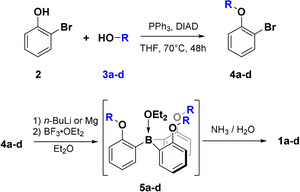
Synthetic routes to the target compounds **1 a**–**d**. The side groups of alcohols **4 a**–**d** are shown in Scheme 1.

Single crystals suitable for X‐ray crystallography were obtained from **1 b** and **1 d** after crystallization from ethanol (cf. Supporting Information). As found by Trauner et al. for **1 a**, the two complexes adopt a propeller conformation in which the alkoxy substituents point towards the ammonia (cf. Figure 1). Experimentally determined NH⋅⋅⋅O distances are below 2.2 Å, suggesting strong hydrogen bonding interactions. To our surprise, the unit cells of the two compounds contain both right‐ or left‐handed propeller structures. Hence, in solid state, no preference in the propeller shape is induced by the chiral field of the alkoxy side groups independent of the distance of the stereocenters to the formal *C*_3_‐axis of the propeller. In this regard it is worth noting that the propeller conformations are also not *C*_3_‐symmetric in solid state as the conformations of the alkoxy groups are not identical.

**Figure 1:**
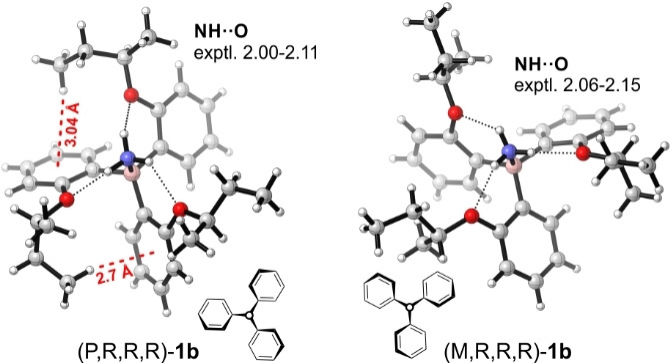
Molecular structures of (*P*,*R*,*R*,*R*)‐ and (*M*,*R*,*R*,*R*)‐**1 b** obtained from X‐ray crystallography.^[19]^ Note that both *P*‐ and *M*‐propeller conformations are found in the same unit cell. Further crystallographic information on **1 b** and **1 d** can be found in the SI file.

Due to packing effects, solid‐state structural preferences may differ from those in solution.^[10]^ Therefore, we used chiroptical spectroscopic methods to probe solution phase properties of the borane–ammonia complexes. In analogy to trityl propellers,^[5]^ we expected that a preferential propeller‐shaped spatial orientation of the aryl blades should give rise to significant electronic circular dichroism (ECD) intensity. No preference for either of the propeller conformations, however, should be reflected in low or no CD intensity.

The experimental UV and ECD spectra of **1 b**–**d** were recorded in CHCl_3_ solutions (cf. Figure 2). In the accessible spectral range of *λ*>240 nm, the UV spectra of all compounds show a band of a phenyl π–π* transition in the range of 260–290 nm that features some vibrational fine structure. Associated with this electronic transition, the CD spectra of **1 b** and **1 c** show negative CD bands for the (*R*)‐enantiomers. For both compounds, the anisotropy factor (g‐factor, g=Δ*ϵ*/*ϵ*) of the transition is g_(270 nm)_≈−10^−3^. For (*S*)‐**1 d**, there is an extremely weak yet observable ECD band associated with the π–π* transition of the aryl rings (g_(270 nm)_≈10^−5^).

**Figure 2:**
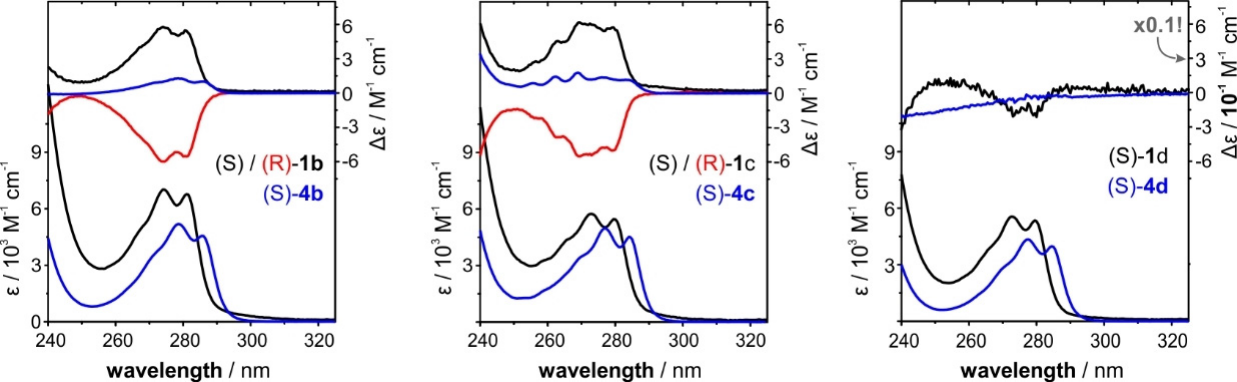
Experimental UV and CD spectra of **1 b**–**d** (black/red) and **4 b**–**d** (blue). The spectra of the blades **4 b**–**d** are multiplied by a factor of 3 for comparison with the data for the propeller molecules. Note that the *y*‐axis of CD spectra of **1 d**/**4 d** is different from those of **1 b**/**4 b** and **1 c**/**4 c**! Experimental conditions can be found in the SI file.

The observed UV bands of the propeller molecules **1 b**–**d** have about three times the intensities of the corresponding UV spectra of the single blades **4 b**–**d**. Compared to the single blades, the absorption band patterns and shapes of **1 b**–**d** did not change significantly and only a small blueshift of the entire band can be noted. The CD signatures of the single blades show a similar vibronic pattern as observed for **1 b**/**c**, but the band intensities **4 b**/**c** are generally significantly weaker. No CD activity is observed for blade **4 d**. Overall it is thus intriguing to consider the increased CD intensity observed for **1 b**/**c** as indication for a preference of one propeller conformation over the other. Likewise, the lack of CD intensity could indicate that there is no such distinct preference for **1 d**.

In order to shed some light on the conformational preferences from a computational perspective, we carried out a comprehensive conformational analysis of (*R*)‐**1 b** on the B3LYP/6‐31+G(2d,p)/IEFPCM(CHCl_3_) level of theory. In our systematic conformational search, we considered both *P*‐ and *M*‐propeller conformations and found a total of more than 100 unique structures (cf. SI for more information). In line with the preliminary conclusions drawn from the experimental ECD spectra, namely that there is a small excess of one propeller conformation over the other, the computed population ratio of all *P*‐conformers to all *M*‐conformers was found to be 54:46. This ratio would thus correspond to an overall 8 % diastereomeric excess (*de*) of the *P*‐propellers.

From a theoretical perspective it should be noted that the observed π–π* transition (denoted ^1^L_b_ in Platt notation) is electrically (and magnetically) forbidden by symmetry in an unsubstituted benzene.^[11]^ It becomes experimentally observable only due to the vibronic coupling with higher states. For chiral derivatives, in which the stereogenic center is attached directly to the ring or in short distance to it, the band is also often found to feature CD intensity (like in our examples **4 b**–**d**). In fact, the CD band may also be used for AC determinations using the semiempirical benzene sector and benzene chirality rules.[^11b^, ^12^] Therefore, we computed the UV and ECD spectra of the twelve lowest‐energy conformers of (*R*)‐**1 b**, that is, six conformers with either (*P*)‐ or (*M*)‐propeller conformation, to evaluate whether the observed CD signature can also be correlated with the predicted preference for *P*‐helicity. The lowest energy π–π* transition was indeed found to feature opposite sign for the (*P*)‐ and (*M*)‐helical propeller conformations, namely positive for (*P*) and negative for (*M*). Therefore, the computed spectra suggest that the experimentally observed negative CD signature of (*R*)‐**1 b** would actually indicate a preference for the *M*‐propeller conformation (cf. Figure S1) rather than for the energetically more favoured *P*‐epimers. While it is noted that the ^1^L_b_ band has been reported to be occasionally challenging to predict correctly due to the complex vibronic coupling patterns,^[13]^ a conclusion drawn on the sign of just one band may also generally not be fully reliable. Nonetheless, it appears as if the computed and experimental CD signatures point towards opposite conformational preferences.

Vibrational circular dichroism (VCD) spectroscopy is extremely sensitive to conformational changes.^[14]^ As it does not rely on a few UV chromophores but on vibrational modes spanning the entire mid‐infrared spectral range,^[15]^ it is the ideal method to confirm the computationally predicted preferences and to gain deeper insight into the dynamic stereochemistry of the borane–ammonia complexes.^[16]^ Figure 3 a shows the experimental IR and VCD spectra of **1 b** recorded in CDCl_3_. It is noteworthy that the few observable IR bands give rise to VCD spectra that are very rich in spectral features. The very good mirror‐image relation between the experimental VCD spectra of the two enantiomers further highlights the numerous spectral features that are available for a detailed characterization of the stereochemical preferences.

**Figure 3:**
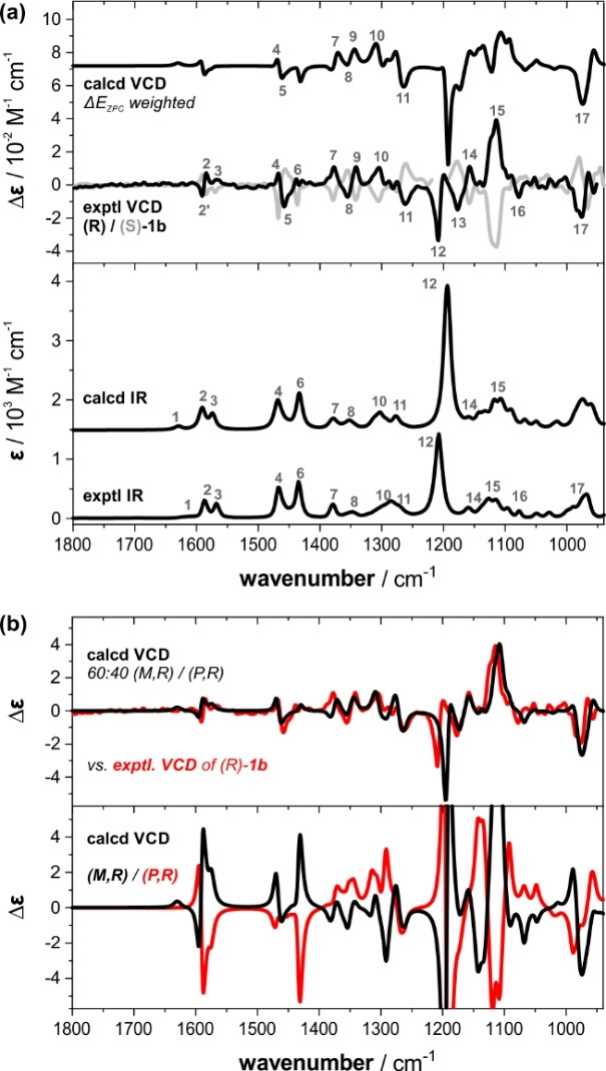
Experimental IR and VCD spectra of **1 b** (52 mm, 100 μm path length, CDCl_3_) compared to computed spectra: a) Comparison with the spectra of (*R*)‐**1 b** obtained by Boltzmann‐weighting over all conformers (Δ*E*_ZPC_). Numbers indicate band assignments. b) VCD spectra computed individually for (*M*,*R*)‐ and (*P*,*R*)‐conformers (bottom) and simulated VCD spectrum assuming a 60:40 ratio of *M*‐ and *P*‐helical propellers.

Based on the predicted conformational preferences and considering all populated conformers, we computed the IR and VCD spectra of (*R*)‐**1 b** that are shown alongside the experimental spectra in Figure 3 a. The good match between the computed and the experimental IR spectra can be noted immediately: All bands are predicted correctly in position and relative intensity. For the VCD spectra, however, the match is less satisfying. Only some major bands, such as bands 4–5 and 7–11, are predicted similar to the experimental pattern. For the bands 12–17, a rough match could be proposed, but one‐to‐one band correlations also reveal quite some mismatches. Finally, the entire pattern in the range of 1600–1550 cm^−1^ (bands 2–3) and also band 6 at 1440 cm^−1^ are obviously inverted in sign and thus do not match with the experimental VCD signatures.

Especially considering the high quality of the experimental spectra, we were not satisfied with the level of agreement and could not consider it as convincing enough to confirm the predicted conformational preferences. Therefore, going into further detail of the spectra predictions, we calculated the IR and VCD spectra of the two propeller conformations of (*R*)‐**1 b** individually. To this end, we co‐added only those structures with either *M*‐ or *P*‐helical propeller conformations while retaining the relative Boltzmann weights within the two sets (cf. Figure 3 b). The so‐obtained IR spectra of the two propeller conformer sets are almost identical with the one initially predicted for the whole set. Their VCD spectra, however, are significantly more intense than both the experiment and the initially predicted VCD spectrum. More importantly, they show a mirror image relation over a wide spectral range, which clearly suggests that the three identical stereogenic centers in the side groups of **1 b** have less influence on the VCD signature than the propeller conformation. In fact, only the bands giving rise to the experimental band pattern 11 feature the same sign in both the *M*‐ and *P*‐propeller VCD spectra. Further detailed comparison of the individual VCD spectra of the *M*‐ and *P*‐propeller conformations reveals that both the pattern in the range of 1600–1550 cm^−1^ (bands 1–3) and band 6 at 1440 cm^−1^, which are incorrectly predicted in the Boltzmann‐weighted spectrum, can be found with correct sign only in the spectrum of the *M*‐conformation. Likewise, several other experimental signatures also seem to arise dominantly from the *M*‐propeller spectrum. Together with the initial ECD spectra analysis, this observation directly led to the strong hypothesis that the contribution of the *M*‐propeller to the experimental spectrum of (*R*)‐**1 b** may be significantly higher than the computationally predicted 46 %.

Figure 3 b shows a simulated VCD spectrum that was obtained by assuming a 60:40 ratio of *M*‐ and *P*‐propellers, which corresponds to 20 % *de* of the *M*‐propeller (as opposed to 8 % *de* for the *P*‐propeller predicted based on Δ*E*_ZPC_). As the direct overlap with the experimental VCD spectrum nicely highlights, this simulated spectrum resembles almost the entire experimental VCD pattern and provides a major improvement over the initially predicted spectrum. A quantitative similarity analysis further confirms that the 60:40 ratio provides the best match with the experimental spectrum (cf. Supporting Information).^[17]^ The very good match of the population‐adjusted VCD spectrum can be seen as a reliable confirmation for an excess of the *M*‐propeller.

Interestingly, the computational VCD spectra analysis of (*R*)‐**1 c** leads to essentially the same conclusions (cf. Supporting Information, Figure S2). Although the conformational space is smaller and the alkoxy group larger, the relative Boltzmann weights suggest an almost 50:50 ratio of *P*‐ and *M*‐propeller conformers. The comparison of the independent spectral features of *P*‐ and *M*‐helical structures, however, indicates again a dominance of the *M*‐propeller conformations and an adjustment of the population ratio towards 60:40 significantly improves the match between experimental and predicted spectra.

As mentioned before, introducing a methylene unit distance between the ether oxygen and the stereogenic center of the side group leads to a drop in CD intensity of the π–π* (^1^L_b_) band for **1 d** as compared to **1 b**/**c** (cf. Figure 2 c), which we tentatively attributed to a lack of a preferential propeller conformation. In this regard, the predicted *M*/*P*‐ratio of 57:43 that was obtained for an ensemble of more than 150 conformers of (*S*)‐**1 d** is unexpected, as it should be closer to 50:50 (i.e., racemic with respect to the propeller helicity). We note, however, that the predicted IR and VCD spectra match quite well with the experimental spectra (Figure 4). Especially the bands in the range of 1600–1550 cm^−1^, whose VCD patterns are markers for the propeller helicity also for **1 d**, are reproduced with the correct sign. Solely the prediction for the pattern of bands 10–12 appears to be off, which can actually be explained by the slight misplacement of band 12 that affects the overall pattern. For a simulated 1:1 mixture of *P*‐ and *M*‐propeller conformations, this pattern would be opposite to the experimental spectra (cf. SI, Figure S3). Hence, the predicted small dominance of the (*P*,*S*)‐conformers of **1 d** seems to match with the experimentally observed conformational preferences.

**Figure 4:**
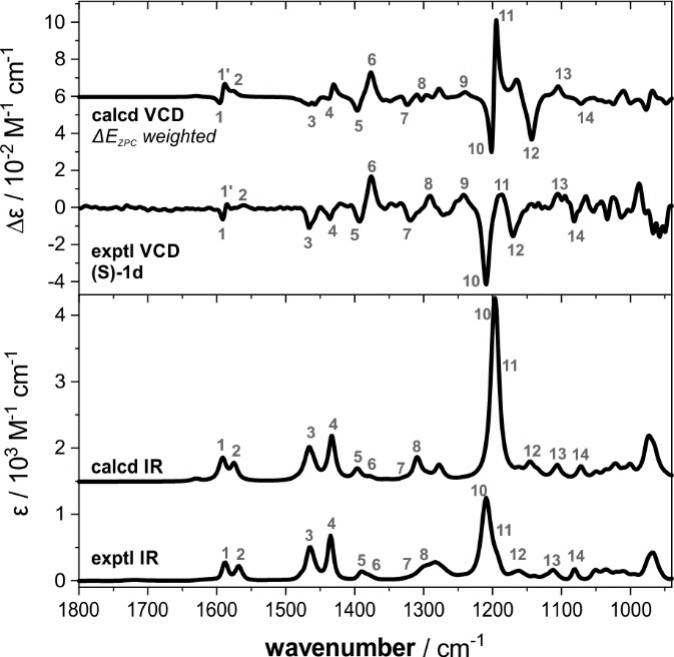
Experimental IR and VCD spectra of (*S*)‐**1 d** (77 mm, 100 μm path length, CDCl_3_) compared to computed spectra obtained by Boltzmann‐weighting over all conformers (Δ*E*_ZPC_). Numbers indicate band assignments.

In order to explain the contradiction between computed conformer energies of **1 b**/**c** and the corresponding preferences proposed based mostly on the VCD analysis, we examined the propeller structures in more detail. As indicated in Figure 1 for the molecular geometries of (*R*)‐**1 b** found in solid state, there are C−H⋅⋅⋅π interactions between the alkoxy substituents and the aryl blades in the (*P*)‐propeller structure, which are not found in the (*M*)‐propeller. Such interaction can be observed for the first five lowest‐energy (*P*)‐propeller conformers and in none of the five lowest‐energy (*M*)‐propellers (cf. Figure S4). Similarly, a C−H⋅⋅⋅π interaction between the phenyl ring of the alkoxy substituent and the aryl blade can be found for (*P*,*R*)‐**1 c**, but not for (*M*,*R*)‐**1 c** (cf. Figure S5). For **1 d**, however, such C−H⋅⋅⋅π interaction can be found in both propeller conformations (cf. Figure S6). This lets us propose that the C−H⋅⋅⋅π interactions have a stabilizing effect in the computations, which are less important in solution phase. This conclusion is further supported by the observation that the inclusion of dispersion corrections using the B3LYP‐GD3BJ and M06‐2X functionals leads to a further shift of the conformational equilibrium towards the (*P*)‐propeller structure. Therefore, we propose that the π‐faces of the aryl blades are preferentially exposed to the solvent with C−H⋅⋅⋅π‐like dispersive solute–solvent interactions dominating over the intramolecular C−H⋅⋅⋅π contacts.

The results presented herein can be summarized in four key points:

1. The crystal structure analysis reveals that the chiral borane–ammonia adducts do not have any preference for a chiral propeller conformation in solid state.
2. Despite initially appearing as the method of choice, the ECD spectra alone were not suitable to unambiguously establish any conformational preferences, but they point towards a preference for (*M*)‐helicity of (*R*)‐**1 b**.
3. The VCD analysis clearly shows a preference for the (*M*)‐helical propeller when the stereogenic centers of the alkoxy side groups in **1 b**/**c** have an (*R*)‐configuration.
4. The ECD and especially the VCD analysis oppose the DFT‐predicted conformational preferences obtained for the isolated, only implicitly solvated molecule, as explicit solute–solvent interactions play a major role in determining the conformational equilibrium.

In conclusion, we have demonstrated that point chirality of alkoxy substituents can indeed introduce a preferential propeller conformation to the (triarylborane) ammonia complex. This preference, however, seems difficult to capture and thus to accurately predict by DFT computations. It must be further explored how increasing the steric bulk of the substituents further affects the conformational equilibrium. Furthermore, using chiral amines instead of chiral side groups as stereochemical inducers could be an interesting way to lock the conformational preferences more efficiently, potentially leading to structures with externally switchable propeller conformations. Lastly, we strive to incorporate such chiral borane propellers in molecular cages.^[18]^

## Conflict of interest

The authors declare no conflict of interest.

## Supporting information

As a service to our authors and readers, this journal provides supporting information supplied by the authors. Such materials are peer reviewed and may be re‐organized for online delivery, but are not copy‐edited or typeset. Technical support issues arising from supporting information (other than missing files) should be addressed to the authors.

Supplementary
