# Supplementary material for: Chiral Molecular Propellers of Triarylborane Ammonia Adducts
Source: Angew Chem Int Ed Engl. 2020 Dec 10;60(6):2958–62. doi: 10.1002/anie.202014130 (PMC7898383; doi:10.1002/anie.202014130)
Supplement: Supplementary file 1 — Supplementary [file ANIE-60-2958-s001.pdf]

Angewandte Chemie
Eine Zeitschrift der Gesellschaft Deutscher Chemiker

Supporting Information

Chiral Molecular Propellers of Triarylborane Ammonia Adducts

*Michael Kemper, Elric Engelage, and Christian Merten**

anie_202014130_sm_miscellaneous_information.pdf

Supporting information

Table of Contents

1. Conformational analysis 2

Conformational analysis of 1b 2

Conformational analysis of 1c 3

Conformational analysis of 1d 5

2. Additional spectra plots 8

Figure S1. Computed UV and ECD spectra of 1b. 8

Figure S2. VCD spectra analysis of 1c. 8

Figure S3. Comparison of several computed VCD spectra of 1d 9

3. Additional figures 10

4. Similarity analysis 12

Figure S7. Similarity analysis of the VCD spectra of 1b/1c 12

5. X-ray crystallography 13

6. Spectroscopic and computational details 17

UV/CD spectroscopy. 17

IR/VCD spectroscopy. 17

Computational details. 17

7. Synthetic procedures 18

8. Selected Cartesian coordinates from computations 34

1. Conformational analysis

Conformational analysis of 1b

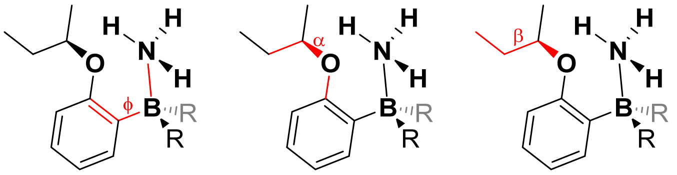

Table S1. Conformational analysis **1b**. We list only the first 50 conformers of more than 150 optimized conformers as Boltzmann weights are neglectable.

	ϕ–1 [°]	α-1 [°]	β-1 [°]	ϕ–2 [°]	α-2 [°]	β-2 [°]	ϕ–3 [°]	α-3 [°]	β-3 [°]	ΔE_{ZPC} [kcal mol^{-1}]	ΔG_{298K} [kcal mol^{-1}]	pop-ΔE_{ZPC} [%]	pop-ΔG_{298K} [%]
**1b**-c1	-45.5	156.2	-66.1	-44.6	156.2	-65.9	-44.5	156.2	-65.9	0.00[a)]	0.84	13.6	2.0
**1b**-c2	46.1	163.9	-67.7	46.4	163.4	-67.9	45.8	163.6	-67.8	0.36	0.80	7.4	2.1
**1b**-c3	-44.8	157.0	-65.6	-46.1	151.3	-174.0	-46.8	76.5	-170.3	0.48	0.00[a)]	6.0	8.2
**1b**-c4	-46.1	77.3	-170.3	-46.4	77.2	-169.7	-45.3	157.3	-65.9	0.50	0.25	5.9	5.3
**1b**-c5	-46.9	151.2	-174.0	-44.9	156.7	-65.9	-46.0	76.0	-170.4	0.59	0.26	5.0	5.3
**1b**-c6	45.7	160.1	-174.4	46.2	163.2	-67.8	46.3	84.3	-170.8	0.74	0.64	3.9	2.8
**1b**-c7	45.8	163.9	-67.8	47.1	162.5	-174.2	47.0	84.7	-170.7	0.75	0.62	3.8	2.9
**1b**-c8	46.8	162.0	-174.2	46.6	160.9	-174.4	46.7	161.3	-174.4	0.80	0.52	3.5	3.4
**1b**-c9	46.7	85.3	-170.6	45.8	84.4	-170.1	47.1	163.5	-67.9	0.81	0.72	3.4	2.4
**1b**-c10	-47.0	152.2	-173.1	-47.6	151.2	-173.7	-46.9	151.3	-173.7	0.83	0.21	3.3	5.8
**1b**-c11	-46.7	151.0	-173.8	-46.8	151.4	-173.9	-46.5	76.0	-170.4	0.85	0.31	3.2	4.9
**1b**-c12	-46.8	77.1	-170.3	-46.0	77.1	-169.8	-46.4	153.4	-173.7	0.88	0.49	3.1	3.6
**1b**-c13	47.2	162.0	-174.2	46.2	160.1	-174.6	46.1	84.4	-170.7	0.93	0.58	2.8	3.1
**1b**-c14	46.7	85.5	-170.6	46.0	84.3	-170.2	47.5	162.2	-174.4	1.00	0.69	2.5	2.6
**1b**-c15	45.1	163.7	-67.8	43.2	126.3	-64.9	47.0	163.9	-67.8	1.29	1.22	1.5	1.1
**1b**-c16	47.2	163.8	-67.8	44.7	163.4	-67.8	43.2	126.3	-64.8	1.29	1.05	1.5	1.4
**1b**-c17	-46.5	148.9	65.1	-45.6	157.0	-66.1	-43.8	156.5	-66.2	1.33	0.73	1.4	2.4
**1b**-c18	47.2	163.8	-67.8	45.9	162.0	-174.1	42.7	125.8	-64.7	1.41	0.67	1.3	2.6
**1b**-c19	-44.3	157.0	-66.0	-46.0	153.8	-173.8	-45.5	80.9	63.5	1.41	1.42	1.2	0.8
**1b**-c20	-46.6	152.6	-173.4	-45.5	157.6	-66.0	-44.4	80.6	63.1	1.42	0.96	1.2	1.6
**1b**-c21	42.9	125.5	-64.8	46.9	163.8	-68.0	45.9	161.6	-174.4	1.47	0.97	1.1	1.6
**1b**-c22	43.2	125.9	-64.9	47.5	85.1	-170.1	44.9	164.0	-67.5	1.48	0.94	1.1	1.7
**1b**-c23	42.6	126.1	-64.8	47.4	164.1	-67.8	45.6	84.5	-170.4	1.51	0.96	1.1	1.6
**1b**-c24	46.6	163.5	-67.9	47.0	161.3	-174.3	46.0	87.2	61.0	1.53	1.06	1.0	1.4
**1b**-c25	46.0	160.6	-174.4	46.8	163.9	-67.9	46.3	87.2	61.0	1.54	1.21	1.0	1.1
**1b**-c26	-44.9	157.1	-65.7	-44.9	157.0	-65.8	-46.1	108.8	-64.8	1.58	1.42	0.9	0.7
**1b**-c27	46.3	161.8	-174.3	46.6	162.2	-174.4	47.1	89.7	61.0	1.62	0.94	0.9	1.7
**1b**-c28	-46.7	148.9	65.4	-45.3	157.0	-66.4	-46.3	151.4	-173.2	1.63	0.46	0.9	3.8
**1b**-c29	-46.4	148.1	65.0	-47.1	76.1	-170.3	-44.6	156.7	-66.1	1.66	1.08	0.8	1.3
**1b**-c30	47.1	161.5	-174.5	45.7	162.0	-174.3	43.1	125.3	-64.8	1.66	1.12	0.8	1.2
**1b**-c31	-46.2	148.2	64.5	-47.5	149.9	-173.7	-45.3	156.8	-65.9	1.66	1.49	0.8	0.7
**1b**-c32	42.8	126.2	-64.9	47.7	162.3	-174.5	45.7	84.7	-170.8	1.69	0.92	0.8	1.7
**1b**-c33	42.5	126.2	-64.9	47.5	161.2	-174.5	45.7	161.5	-174.3	1.70	0.93	0.8	1.7
**1b**-c34	-46.4	150.7	-173.9	-47.1	151.1	-173.7	-45.0	80.5	62.8	1.73	1.53	0.7	0.6

**1b**-c35	43.1	126.5	-65.2	46.9	84.0	-170.3	45.5	160.4	-174.4	1.75	1.13	0.7	1.2
**1b**-c36	46.7	161.2	66.1	46.3	164.0	-67.7	46.2	162.8	-174.3	1.78	1.88	0.7	0.3
**1b**-c37	-47.3	150.2	-174.1	-44.1	157.2	-66.3	-46.9	146.6	65.4	1.80	1.92	0.7	0.3
**1b**-c38	47.9	161.4	66.2	45.9	161.5	-174.3	46.2	163.8	-68.0	1.80	1.73	0.6	0.4
**1b**-c39	-46.3	150.5	65.6	-45.4	156.5	-66.1	-46.1	77.2	-169.7	1.80	1.96	0.6	0.3
**1b**-c40	46.7	159.7	66.4	45.7	83.9	-169.9	46.9	164.1	-67.8	1.86	1.83	0.6	0.4
**1b**-c41	-45.2	109.7	-64.3	-46.9	76.6	-170.0	-45.5	156.7	-66.2	1.87	0.70	0.6	2.5
**1b**-c42	-44.6	156.8	-65.6	-46.2	151.7	-173.5	-46.9	109.2	-64.9	1.88	1.27	0.6	1.0
**1b**-c43	46.1	161.9	-174.2	46.0	164.0	-67.9	47.4	161.0	66.7	1.90	2.41	0.5	0.1
**1b**-c44	46.0	158.9	66.1	46.6	163.9	-67.8	46.7	85.4	-170.0	1.93	1.86	0.5	0.4
**1b**-c45	-47.1	108.6	-64.6	-44.7	156.4	-65.7	-46.5	151.5	-173.5	1.93	1.47	0.5	0.7
**1b**-c46	-46.6	108.2	-64.5	-45.0	156.5	-66.1	-46.2	77.1	-170.1	1.98	1.87	0.5	0.3
**1b**-c47	47.6	160.6	66.5	45.4	84.3	-170.4	46.5	161.0	-174.2	2.01	1.59	0.5	0.6
**1b**-c48	-47.2	148.2	63.4	-46.1	76.5	-169.8	-46.1	152.8	-173.7	2.02	1.49	0.4	0.7
**1b**-c49	-47.5	148.6	64.7	-47.2	150.9	-173.5	-46.8	77.0	-170.2	2.04	1.52	0.4	0.6
**1b**-c50	47.1	161.4	66.9	46.7	161.6	-174.1	47.0	86.9	-170.5	2.06	1.50	0.4	0.6
											Σ =	97.0%	95.5%

a) referenced to E_{ZPC}(**1b**-c1) = -1473.297383 hartree and G_{298K}(**1b**-c3) = -1473.368315 hartree.

Conformational analysis of 1c

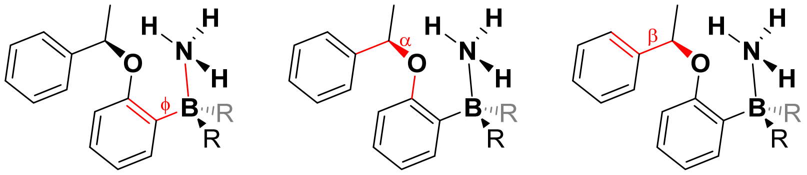

Table S2. Conformational analysis **1c**.

	ϕ-1 [°]	α-1 [°]	β-1 [°]	ϕ-2 [°]	α-2 [°]	β-2 [°]	ϕ-3 [°]	α-3 [°]	β-3 [°]	ΔE_{ZPC} [kcal mol^{-1}]	ΔG_{298K} [kcal mol^{-1}]	pop-ΔE_{ZPC} [%]	pop-ΔG_{298K} [%]
**1c**-c1	-45.0	77.5	-151.7	-45.5	77.1	-151.8	-45.2	76.9	-151.9	0.00[a)]	0.51	48.3	17.8
**1c**-c2	47.3	84.8	-150.9	46.8	83.5	-152.8	47.5	84.0	-152.6	0.29	0.00[a)]	29.5	41.8
**1c**-c3	47.1	84.7	-149.9	46.5	158.4	-122.2	47.7	86.1	-149.5	0.81	0.53	12.2	17.1
**1c**-c4	48.0	86.8	-148.7	47.0	160.2	-124.2	47.3	161.2	-123.9	1.36	1.13	4.8	6.2
**1c**-c5	47.0	160.3	-126.1	48.2	162.3	-126.3	47.1	160.2	-125.5	1.69	0.58	2.8	15.8
**1c**-c6	-46.4	77.7	-151.3	-44.0	153.5	-114.8	-45.8	76.7	-152.2	1.82	2.16	2.2	1.1
**1c**-c7	-46.3	77.8	-151.5	-45.9	151.5	-113.3	-45.4	151.4	-113.9	3.59	3.44	0.1	0.1
**1c**-c8	-45.4	76.8	-151.2	-44.9	-77.6	-36.8	-45.6	76.9	-151.1	4.70	4.76	0.0	0.0
**1c**-c9	46.9	84.8	-152.6	46.8	-67.8	-37.3	46.0	82.1	-152.4	5.11	4.37	0.0	0.0
**1c**-c10	-46.7	154.4	-114.3	-46.0	151.6	-112.9	-45.6	152.7	-115.7	5.42	4.48	0.0	0.0
**1c**-c11	44.5	83.0	-151.8	47.2	158.3	-122.4	47.7	-67.5	-37.4	5.65	5.37	0.0	0.0
**1c**-c12	45.6	-68.2	-37.0	46.9	159.1	-121.8	48.2	85.8	-150.1	5.71	5.92	0.0	0.0
**1c**-c13	-47.0	155.5	-6.6	-45.5	151.7	-112.7	-45.7	152.3	-115.3	5.85	5.24	0.0	0.0
**1c**-c14	-45.1	154.2	127.0	-46.3	152.1	-112.7	-45.2	153.0	-115.6	5.88	5.58	0.0	0.0
**1c**-c15	47.0	-68.7	-36.8	46.5	160.2	-124.8	47.5	160.4	-123.6	6.14	5.88	0.0	0.0
**1c**-c16	-47.4	155.3	-4.0	-44.4	152.4	-112.3	-46.1	153.4	137.2	6.29	5.62	0.0	0.0
**1c**-c17	-46.4	77.6	-151.1	-44.0	152.2	-112.7	-45.6	-78.4	-36.5	6.40	6.06	0.0	0.0

**1c**-c18	-45.2	153.0	125.3	-46.9	155.1	-2.6	-44.3	154.5	136.7	6.55	5.55	0.0	0.0
**1c**-c19	-44.3	154.0	127.0	-45.0	155.2	127.3	-45.7	154.2	140.0	6.63	6.51	0.0	0.0
**1c**-c20	-47.0	-79.0	-32.0	-43.2	154.0	-111.6	-45.5	76.5	-152.0	6.63	6.32	0.0	0.0
**1c**-c21	-44.2	77.2	-150.8	-37.6	164.5	-133.7	-46.5	-77.7	-37.3	6.84	6.50	0.0	0.0
**1c**-c22	-46.4	154.5	-7.3	-46.2	154.9	-9.2	-46.1	154.7	-9.1	6.85	6.03	0.0	0.0
**1c**-c23	-47.7	-79.9	-31.5	-44.8	153.2	-112.6	-44.2	153.2	-113.4	8.45	7.63	0.0	0.0
**1c**-c24	45.3	82.2	-152.0	46.9	-68.1	-36.8	46.2	-67.5	-36.9	10.02	10.40	0.0	0.0
**1c**-c25	45.2	-69.0	-37.2	46.2	158.5	-122.7	47.5	-68.4	-36.8	10.45	10.67	0.0	0.0
**1c**-c26	-47.2	-78.8	-32.7	-42.5	153.8	-112.3	-45.8	-79.1	-35.2	11.29	11.05	0.0	0.0
**1c**-c27	-45.4	-79.9	-37.5	-37.0	168.6	-135.3	-45.4	-77.3	-37.8	11.82	11.48	0.0	0.0
											Σ =	99.9%	99.9%

[a] referenced to E_{ZPC}(**1c**-c1) = -1930.522226 hartree and G_{298K}(**1c**-c2) = -1930.60258 hartree.

Conformational analysis of 1d

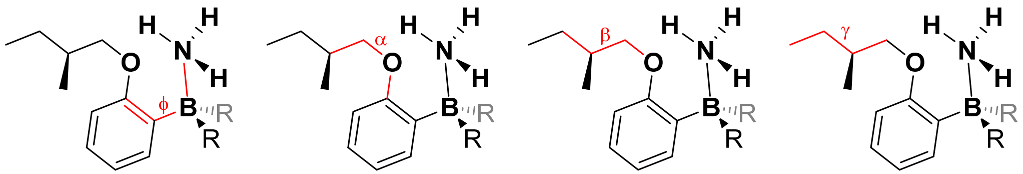

Table S3. Conformational analysis **1d**. We list only the first 50 conformers of more than 150 optimized conformers as Boltzmann weights are neglectable.

	**ϕ-1** [°]	**α-1** [°]	**β-1** [°]	**γ-1** [°]	**ϕ-2** [°]	**α-2** [°]	**β-2** [°]	**γ-2** [°]	**ϕ-3** [°]	**α-3** [°]	**β-3** [°]	**γ-3** [°]	**ΔE_{ZPC}** [kcal mol^{-1}]	**ΔG_{298K}** [kcal mol^{-1}]	**pop-ΔE_{ZPC}** [%]	**pop-ΔG_{298K}** [%]
**1d**-c1	-47.0	177	-65.5	169.4	-45.9	177.7	-65.5	169.5	-47.5	176.2	-65.6	169.5	0.00[a)]	0.28	9.2	4.4
**1d**-c2	45.9	-178.2	-170.7	172.4	46.7	-177.3	-170.7	172.5	46.2	-177	-171	172.6	0.16	0.19	7.0	5.1
**1d**-c3	46.1	-178.6	-170.5	172.6	46.3	-178	-170.9	172.4	46.0	-178.2	-171.5	69.3	0.37	0.55	4.9	2.8
**1d**-c4	46.7	-178.6	-170.3	172.5	46.1	-177.5	-171	172.4	47.4	-173.6	-68.5	169.1	0.40	0.56	4.7	2.7
**1d**-c5	46.0	-178.2	-170.4	172.6	46.1	-178.1	-171.4	69.2	46.3	-178.2	-171.3	69.5	0.53	0.79	3.8	1.8
**1d**-c6	47.0	-175.1	-68.9	168.7	46.3	-177.6	-171	172.6	45.8	-177.8	-170.7	69.7	0.54	0.28	3.7	4.4
**1d**-c7	46.5	-178.6	-170.4	172.6	47.0	-173.6	-68.6	169.7	47.7	-173.3	-68.4	169.2	0.56	0.23	3.6	4.8
**1d**-c8	47.1	-175.1	-68.9	168.8	46.2	-178.3	-171.2	69.5	45.6	-177.4	-170.1	70	0.64	0.06	3.1	6.3
**1d**-c9	46.3	-178.5	-170.5	69.6	46.3	-177.9	-171.3	69.2	46.2	-178.2	-171.2	69.5	0.70	0.66	2.8	2.3
**1d**-c10	-48.1	173.9	-167.8	173.2	-47.4	173.2	-167.8	173.1	-45.0	178.7	-65.4	169.8	0.71	0.27	2.8	4.4
**1d**-c11	46.9	-175.2	-68.8	168.6	46.0	-178.4	-171.5	69.2	46.4	-178.3	-171.3	69.4	0.78	1.00	2.5	1.3
**1d**-c12	47.7	-174.3	-68.5	168.8	47.6	-173.1	-68.4	169.8	47.1	-174.7	-68.9	168.5	0.81	0.38	2.3	3.7
**1d**-c13	-46.0	178.1	-64.9	170.2	-47.3	173.9	-167.8	173	-46.7	174.9	-167.2	70.9	0.82	0.37	2.3	3.8
**1d**-c14	-46.3	179.1	-65	169.9	-47.1	174.3	-167.4	173.5	-47.0	175	-167	70.8	0.88	0.88	2.1	1.6
**1d**-c15	-47.1	174.2	-167.5	173.2	-47.0	174.6	-167	173.4	-47.7	174.2	-167.5	173.2	0.99	0.55	1.7	2.8
**1d**-c16	-47.1	174.6	-167.1	173.5	-47.8	173.9	-167.4	173.3	-47.5	174.6	-167.3	173.4	0.99	0.00[a)]	1.7	7.0
**1d**-c17	-46.4	179.2	-65	170	-46.9	174.5	-167.3	71	-47.0	175.1	-166.9	70.9	1.00	0.56	1.7	2.7
**1d**-c18	-47.4	174	-167.3	173.3	-47.2	174.9	-167.1	173.3	-46.7	174.6	-167.4	173.2	1.00	0.21	1.7	4.9
**1d**-c19	-46.2	177.4	-64.8	169.8	-47.3	174.6	-167.2	71	-46.8	175.6	-167.3	71	1.02	0.75	1.6	2.0
**1d**-c20	-47.1	174.4	-167.2	173.2	-47.3	174.3	-167.5	173.1	-47.1	174.4	-167.6	173.3	1.03	0.64	1.6	2.4
**1d**-c21	-46.1	177.3	-65.2	169.7	-46.4	179	-64.9	170.1	-46.1	178.5	-62.6	-65.9	1.03	1.12	1.6	1.1
**1d**-c22	46.5	-179.2	-171.2	172.4	45.8	-178.5	-170.9	172.4	46.6	-174.3	64.1	169	1.18	1.57	1.3	0.5

**1d**-c23	-47.9	174.4	-167.5	173.4	-47.4	173.3	-167.7	173.1	-46.4	175.8	-166.7	71.2	1.21	0.53	1.2	2.9
**1d**-c24	-47.0	175	-167.4	173.2	-47.1	174.1	-167.3	71	-46.9	174.9	-167	70.9	1.29	0.42	1.0	3.4
**1d**-c25	46.5	-178.8	-170.4	172.6	46.2	-177.8	-170.9	172.4	46.1	-177.2	-166.4	-62.3	1.34	1.79	1.0	0.3
**1d**-c26	45.9	-178.8	-170.9	172.5	47.0	-173.9	-68.7	169.6	46.7	-174.5	64	169.1	1.36	1.54	0.9	0.5
**1d**-c27	47.3	-175	64.4	169.2	46.2	-177.9	-170.8	172.5	45.5	-178.3	-171.5	69.2	1.36	1.80	0.9	0.3
**1d**-c28	47.2	-175.4	-68.9	168.6	45.8	-177.8	-170.8	172.4	45.6	-176	62.5	62.9	1.38	1.73	0.9	0.4
**1d**-c29	-46.4	174.3	-167.5	173.1	-46.3	177.7	-64.4	170	-47.2	174.5	64.8	163.7	1.39	1.27	0.9	0.8
**1d**-c30	46.4	-178.2	-170.3	172.5	46.5	-175.7	63.9	169.3	47.5	-173.5	-68.5	169.1	1.40	1.65	0.9	0.4
**1d**-c31	-45.5	178.4	-64.9	169.6	-47.7	174.8	-163.5	-61.7	-46.5	176.5	-64.9	169.8	1.44	1.30	0.8	0.8
**1d**-c32	-46.7	174.9	-167.7	173	-46.3	175	64.9	164.5	-46.5	176.7	-64.7	169.9	1.45	1.93	0.8	0.3
**1d**-c33	-45.5	178.8	-65.1	169.7	-47.2	175.5	-167.2	173.4	-46.2	179.3	-61.3	-64.6	1.46	1.46	0.8	0.6
**1d**-c34	46.1	-178.4	-170.3	172.6	46.1	-178.1	-171.4	69.2	46.4	-177.5	-166.4	-62.3	1.47	1.77	0.8	0.4
**1d**-c35	46.0	-178.6	-170.7	172.5	46.2	-177.9	-166.2	-62.3	46.0	-178.3	-171.5	69.3	1.47	1.72	0.8	0.4
**1d**-c36	-46.2	175.3	64.6	163.7	-46.0	178.2	-64.7	169.9	-47.8	173.5	-167.9	70.6	1.48	1.28	0.8	0.8
**1d**-c37	-46.0	177.5	-64.9	170	-46.8	175.1	-167.3	173.4	-47.0	173.9	63.4	57.7	1.48	1.77	0.8	0.4
**1d**-c38	-47.2	174.6	-167	71.1	-46.7	175.1	-166.7	71.3	-47.5	175.6	-166.6	71.2	1.48	0.94	0.8	1.4
**1d**-c39	-45.8	177.2	-64.8	169.9	-47.0	174.1	64	163	-47.1	173.9	-168.1	70.6	1.52	1.86	0.7	0.3
**1d**-c40	47.4	-174.8	64.9	169.4	46.0	-178.2	-171.2	69.3	45.7	-178	-171.4	69.3	1.53	1.80	0.7	0.3
**1d**-c41	46.9	-175.4	-68.8	168.5	45.9	-178.2	-171.2	172.2	46.3	-177.4	-166.6	-62.3	1.54	1.95	0.7	0.3
**1d**-c42	46.3	-175.2	64.4	168.7	47.4	-174.1	-69	168.6	45.8	-178.2	-170.4	69.9	1.56	1.44	0.7	0.6
**1d**-c43	47.0	-175.9	63.1	64.2	46.1	-177.9	-171.2	69.3	46.2	-177.3	-171.1	69.5	1.61	2.20	0.6	0.2
**1d**-c44	46.8	-174.9	-68.9	168.9	46.6	-175.5	64	168.4	45.6	-178.3	-170.2	69.8	1.62	1.92	0.6	0.3
**1d**-c45	47.0	-175.6	-68.9	168.7	46.4	-178.1	-171.1	69.5	45.6	-177.4	-165.9	-62	1.63	1.55	0.6	0.5
**1d**-c46	47.2	-175.2	-68.8	168.7	46.0	-177.9	-166.4	-62.3	46.5	-176.3	-170.1	69.8	1.65	1.57	0.6	0.5
**1d**-c47	-47.7	173.3	-167.7	173.2	-46.1	174.5	-167	173.5	-47.2	174.9	64.5	163.6	1.65	1.26	0.6	0.8
**1d**-c48	47.3	-175.2	64.2	168.8	47.2	-173.3	-68.5	169.8	47.2	-173.5	-68.6	169.2	1.67	1.94	0.5	0.3
**1d**-c49	47.5	-175.1	-69	168.4	45.8	-178	-170.9	172.3	47.0	-172.9	-64	-62.3	1.68	1.77	0.5	0.4
**1d**-c50	46.6	-178.6	-170.7	69.4	46.2	-177.9	-171.3	69.2	46.3	-177.5	-166.4	-62.2	1.68	1.91	0.5	0.3
														Σ =	89.1%	91.7%

[a] referenced to E_{ZPC}(**1d**-c1) = -1591.157238 hartree and G_{298K}(**1d**-c16) = -1591.23554 hartree.

Table S4. Comparison of the computed relative zero-point corrected and Gibbs Free Energies (ΔE_{ZPC} and ΔG_{298K}) of the lowest energy conformers of **1b** obtained at different levels of theory.

B3LYP/6-31+G(2d,p)			B3LYP-GD3BJ/6-31+G(2d,p)			B3LYP/6-311++G(2d,2p)			M06-2X/6-311++G(2d,2p)		
**conf. No.**	**pop-ΔE_{ZPC}** [%]	**pop-ΔG_{298K}** [%]	**conf. No.**	**pop-ΔE_{ZPC}** [%]	**pop-ΔG_{298K}** [%]	**conf. No.**	**pop-ΔE_{ZPC}** [%]	**pop-ΔG_{298K}** [%]	**conf. No.**	**pop-ΔE_{ZPC}** [%]	**pop-ΔG_{298K}** [%]
P-**1b**-c1	21.8	4.1	*P*-**1b**-c1	79.1	44.8	*P*-**1b**-c1	23.9	4.2	*P*-**1b**-c1	51.6	22.4
M-**1b**-c2	12.0	4.4	*M*-**1b**-c2	10.1	33.4	*M*-**1b**-c2	13.3	3.7	*P*-**1b**-c17	1.2	0.9
P-**1b**-c3	9.6	16.9	*P*-**1b**-c4	2.8	2.8	*P*-**1b**-c3	9.1	13.6	*M*-**1b**-c2	1.9	6.2
P-**1b**-c4	9.4	11.0	*P*-**1b**-c3	2.3	4.7	*P*-**1b**-c4	8.9	8.0	*P*-**1b**-c4	1.6	1.6
P-**1b**-c5	8.0	10.8	*P*-**1b**-c5	2.3	3.8	*P*-**1b**-c5	7.8	10.2	*P*-**1b**-c5	2.0	1.9
M-**1b**-c6	6.2	5.7	*M*-**1b**-c7	0.7	2.1	*M*-**1b**-c7	6.3	8.2	*P*-**1b**-c3	3.8	7.3
M-**1b**-c7	6.2	5.9	*M*-**1b**-c6	0.6	1.5	*M*-**1b**-c6	6.3	5.5	*P*-**1b**-c20	23.3	39.2
M-**1b**-c8	5.7	7.0	*P*-**1b**-c12	0.5	1.8	*M*-**1b**-c9	5.8	4.4	*M*-**1b**-c6	1.2	1.7
M-**1b**-c9	5.5	5.0	*M*-**1b**-c9	0.4	1.1	*P*-**1b**-c10	4.8	22.3	*M*-**1b**-c8	9.6	8.4
P-**1b**-c10	5.4	11.8	*M*-**1b**-c8	0.4	1.2	*M*-**1b**-c8	4.6	5.3	*M*-**1b**-c15	1.2	1.8
P-**1b**-c11	5.2	10.1	*P*-**1b**-c11	0.4	1.8	*P*-**1b**-c11	4.6	9.4	*P*-**1b**-c19	1.2	1.6
P-**1b**-c12	4.9	7.4	*P*-**1b**-c10	0.2	1.0	*P*-**1b**-c12	4.4	5.2	*M*-**1b**-c7	1.3	6.9
**P/M-ratio**[a)]	**64.4/ 35.6**	**72.0/ 28.0**		**87.7/ 12.3**	**60.7/ 39.3**		**68.5/ 36.5**	**73.0/ 27.0**		**85.5/ 14.5**	**79.6/ 20.4**

[a)] We note that the P/M ratio of the B3LYP/6-31+G(2d,p) is different from the 54:46 reported in the main text and from Table S1 as the ratio given here is calculated over only the first 12 conformers, not over the entire set.

2. Additional spectra plots

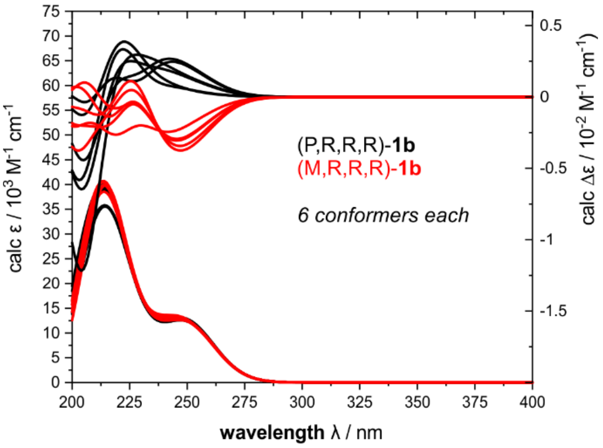

Figure S1. Computed UV and ECD spectra of **1b**.

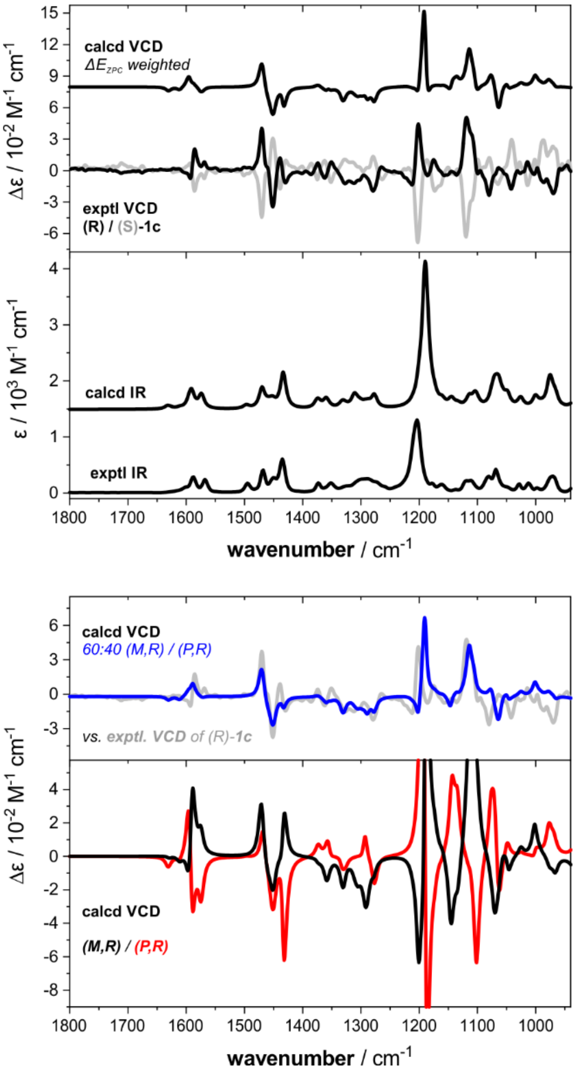

Figure S2. VCD spectra analysis of 1c. For more information, see main text.

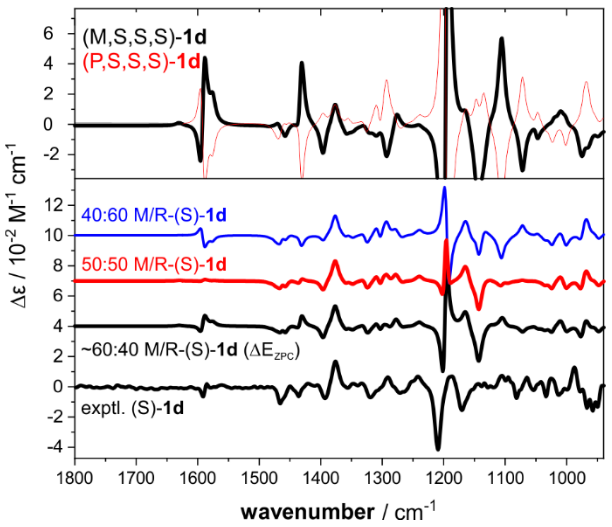

Figure S3. Comparison of several computed VCD spectra of **1d** with the experimental spectrum.

3. Additional figures

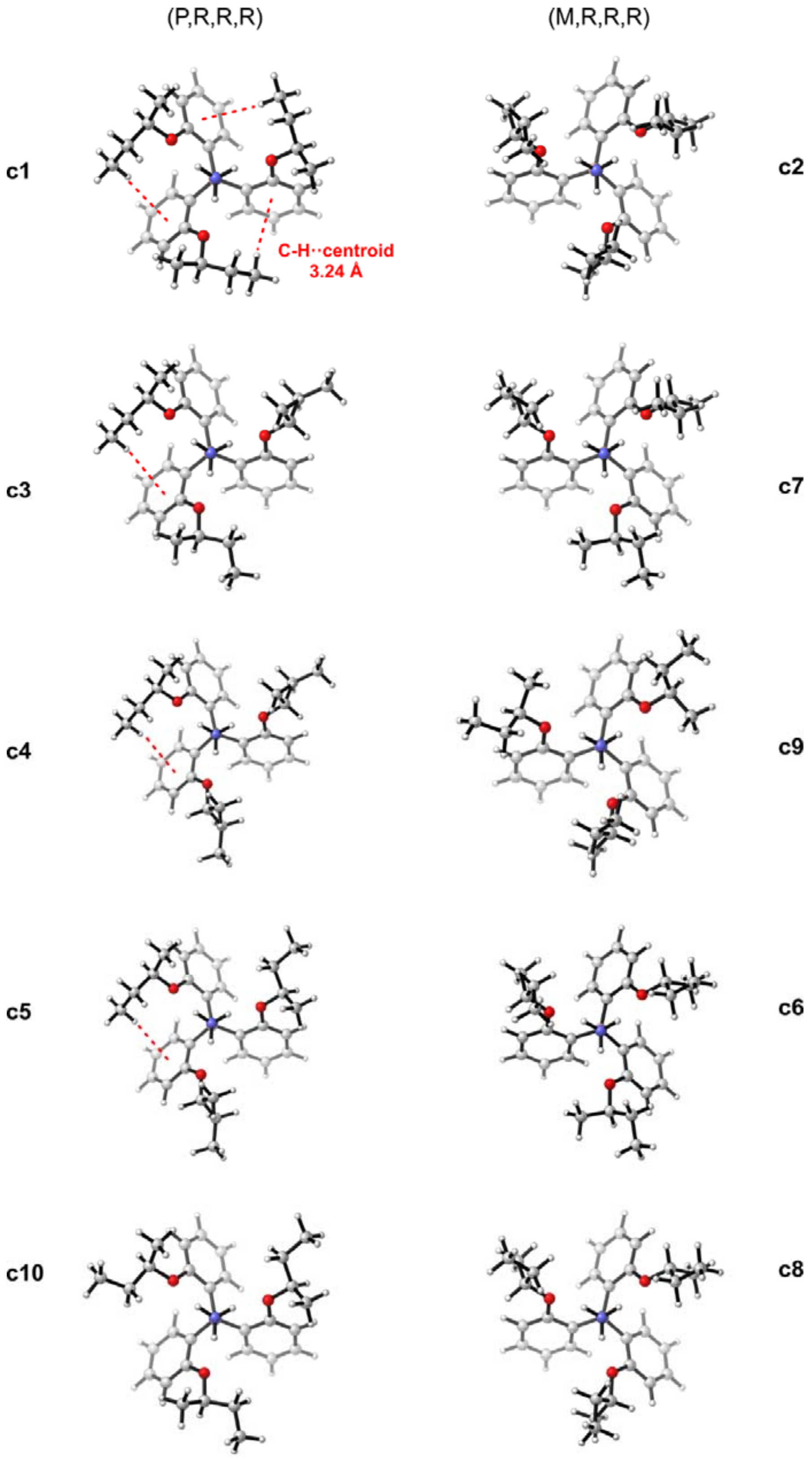

Figure S4. Structures of the ten lowest energy conformers of **1b**.

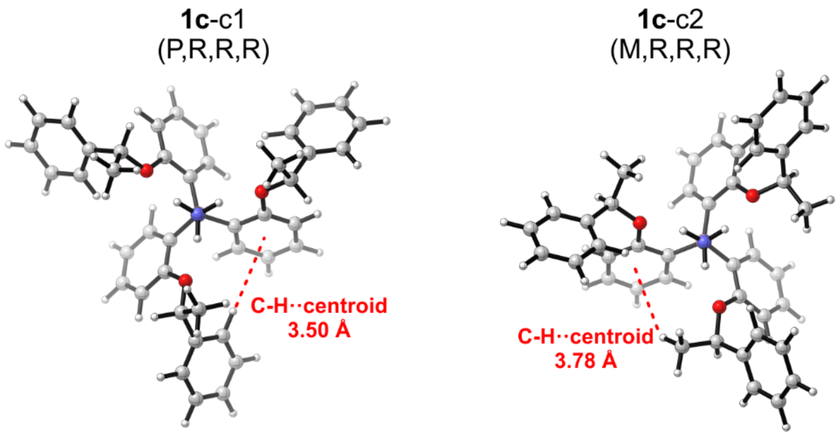

Figure S5. Structures of the two lowest energy conformers of **1c**.

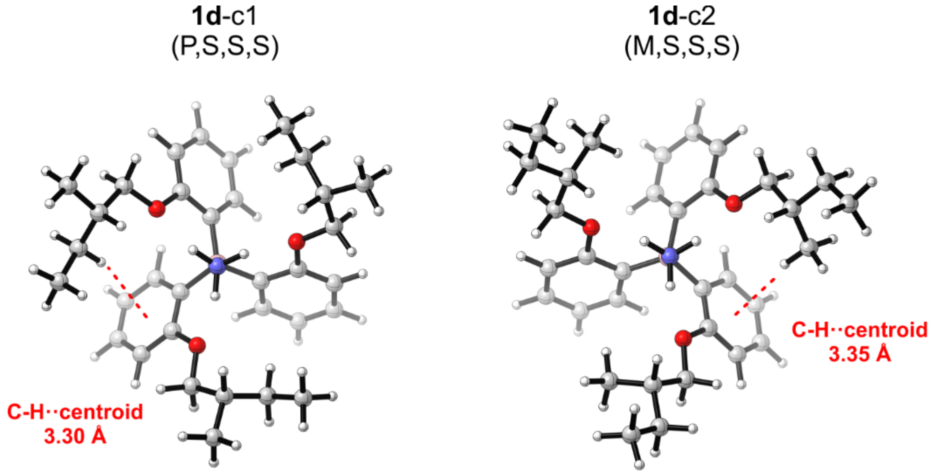

Figure S6. Structures of the two lowest energy conformers of **1d**.

4. Similarity analysis

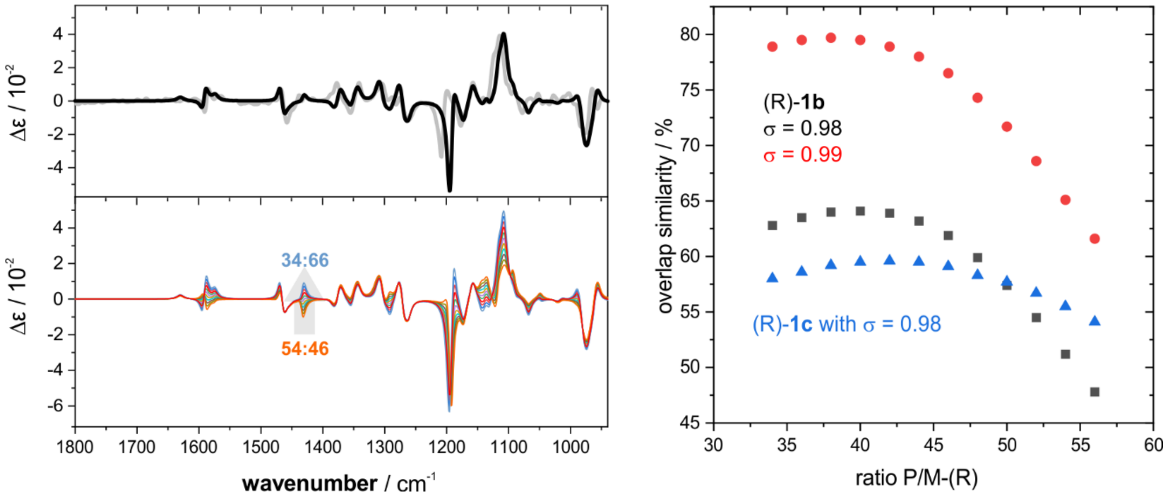

Figure S7. Similarity analysis of the VCD spectra of **1b**/**1c** simulated with different ratios of P/M-helical form.

In order to determine the optimum ratio of P/M-helical propeller conformers of **1b**, we simulated VCD spectra with different ratios and computed the corresponding overlap integrals with the experimental VCD. The left panel of Figure S7 shows the changes in the spectra, that are observable when going from a 54:46 ratio towards 34:66, and the spectral overlap obtained for a P/M = 40:60 ratio as presented in the main text. The right panel shows the computed similarity measures (Polavarapu, *Chirality* **2014,** *26*, 539–552) obtained for two different frequency scaling factors σ. Generally higher similarities are obtained for σ=0.99 than for σ=0.98. This is easily explained as for the larger σ the overlap of the strong bands is optimized, while for σ=0.98, the overlap is better for the many weak bands. In the top-left panel and throughout the main text, we therefore also use σ=0.98 for presentation of the results.

5. X-ray crystallography

Single crystals analyzed on a *Rigaku* Synergy dual source device, with Co micro focus sealed tube (Cu K_α) using mirror monochromators and a HyPix-6000HE: Hybrid photon counting X-ray detector. The crystals were mounted in *Hampton* CrypLoops using *GE/Bayer* silicone grease. Data was recorded and reduced using the CrysalisPro[1] Software. The structure was solved using WinGX[2] in combination with ShelXT[3] and refined with shelXle[4] and ShelXL. Tables for the publication were generated using a modified version of Ciftab. The pictures were generated with Diamond 4[5]

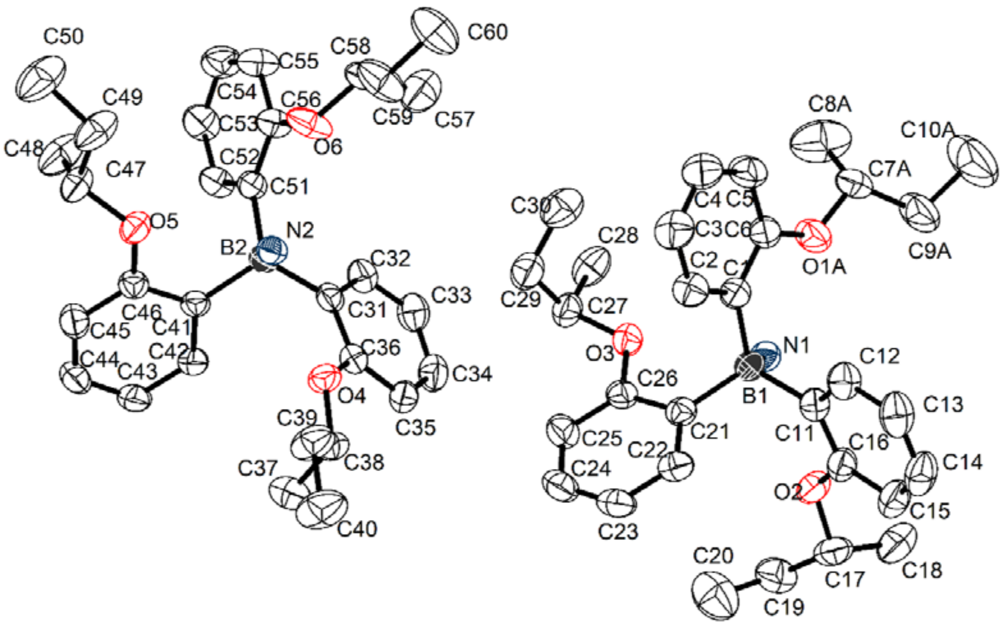

Figure S8. Asymmetric unit of **1b.**

1 Meyer, M.; Paciorek, W.; Kowalski, A.; Muszynski, A.; Wisniewski, A.; Pol, M.; Przewozniczek, M.; Stec, P.; Bujnik, D.; Kulza, H. *et al.* *CrysAlisPro*; Rigaku Oxford Diffraction (1995-2018), 2018.

2 Farrugia, L. J. WinGX suite for small-molecule single-crystal crystallography. *J. Appl. Crystallogr.* **1999**, *32*, 837–838, DOI: 10.1107/S0021889899006020.

3 Sheldrick, G. M. A short history of SHELX. *Acta Crystallogr A Found Crystallogr* **2008**, *64*, 112–122, DOI: 10.1107/S0108767307043930.

4 Hübschle, C. B.; Sheldrick, G. M.; Dittrich, B. ShelXle: a Qt graphical user interface for SHELXL. *J. Appl. Crystallogr.* **2011**, *44*, 1281–1284, DOI: 10.1107/S0021889811043202.

5 Putz, H.; Brandenburg, K. *Diamond*; Crystal Impact: Bonn, Deutschland, 2014

Table S5. Crystal data and structure refinement for **1b**.

Empirical formula	C30 H42 B N O3
Formula weight [g/mol]	475.45
Crystal system	Orthorhombic
Space group	$P2_12_12_1$ (19)
Lattice parameters [Å]	
a	13.14303(11)
b	20.25974(17)
c	21.34921(16)
α	90
β	90
γ	90
Density [g/cm^3]	1.111
Crystal size [mm^3]	0.332 x 0.076 x 0.070
Volume [Å^3]	5684.75(8)
Z	8
Temperature [K]	169.99(10)
Diffraction Device	XtaLAB Synergy, Dualflex, HyPix
Radiation Type	1.54184 Å (Cu K/ micro-focus sealed X-ray tube)
F(000)	2064
Absorption coefficient [mm^{-1}]	0.542
Absorption correction	Semi-empirical from equivalents
Measurement range	3.0 - 66.5
Index range	-15 < h < 15
	-24 < k < 24
	-25 < l < 21
Measured reflexes	67090
Independent	10028
Observed	9299
R(int)	0.0514
Completeness (%) / theta (°)	100.0 / 66.496
Transmission (min / max)	0.91188 / 1.00000
R1 (observed/all)	0.0405 / 0.0435
wR2 (observed/all)	0.1137 / 0.1164
GooF = S	1.049
Rest electron density max./min. [e-/Å^3]	-0.204 / 0.345

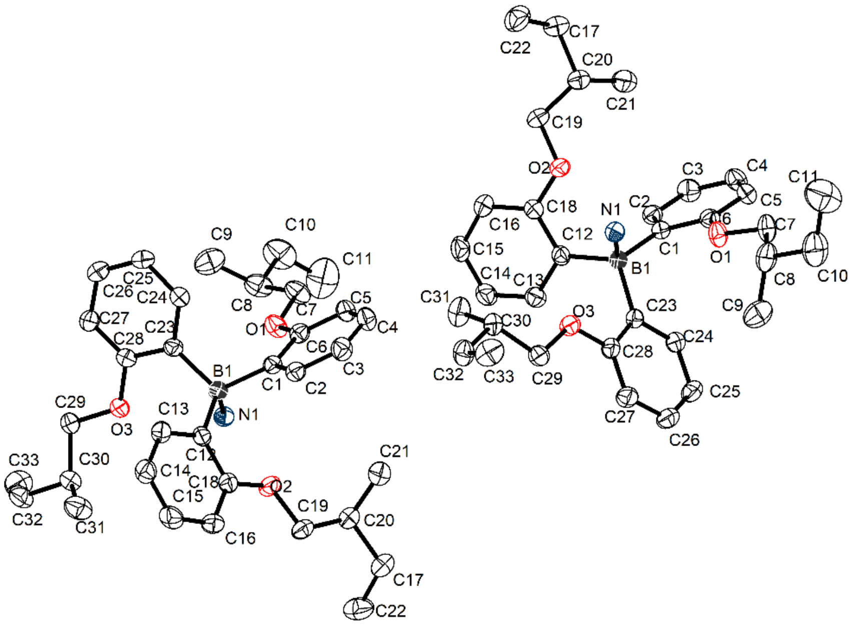

Figure S9. Asymmetric unit of **1d**.

**Table S6.** Crystal data and structure refinement for **1d**.	
Empirical formula	C33 H48 B N O3
Formula weight [g/mol]	517.53
Crystal system	Orthorhombic
Space group	$P2_12_12_1$ (19)
Lattice parameters [Å]	
a	12.04430(10)
b	21.8882(2)
c	23.4210(2)
α	90
β	90
γ	90
Density [g/cm^3]	1.113
Crystal size [mm^3]	0.613 x 0.133 x 0.102
Volume [Å^3]	6174.43(9)
Z	8
Temperature [K]	169.99(10)
Diffraction Device	XtaLAB Synergy, Dualflex, HyPix
Radiation Type	1.54184 Å (Cu K/ micro-focus sealed X-ray tube)
F(000)	2256
Absorption coefficient [mm^{-1}]	0.534
Absorption correction	Semi-empirical from equivalents
Measurement range	2.8 - 66.5
Index range	-10 < h < 14
	-25 < k < 26
	-27 < l < 27
Measured reflexes	56405
Independent	10400
Observed	9919
R(int)	0.0569
Completeness (%) / theta (°)	98.6 / 66.494
Transmission (min / max)	0.30670 / 1.00000
R1 (observed/all)	0.0417 / 0.0434
wR2 (observed/all)	0.1118 / 0.1136
GooF = S	1.021
Rest electron density max./min. [e-/Å^3]	-0.192 / 0.386

6. Spectroscopic and computational details

UV/CD spectroscopy.

UV-Vis and CD Spectra were recorded on an Applied Physics Chirascan™-plus CD Spectrometer in chloroform at room temperature. The quartz cuvettes used had a path length of 1 mm. Concentrations were c(**1b**) = 1.1 mM, c(**1c**) = 0.8 mM, c(**1d**) = 1.0 mM, c(**5b**) = 6.3 mM, c(**5c**) = 4.8 mM and c(**5d**) = 5.8 mM. Spectra were acquired in the spectral range of 240 to 340 nm with a step size of 0.5 nm between measurement points and a sampling time of one second per point. Measurements were repeated eight times and averaged to reduce the signal-to-noise ratio.

IR/VCD spectroscopy.

The IR and VCD spectra were recorded on a Bruker Vertex 70 equipped with a PMA 50 unit for polarization modulated measurements. Samples were held in BaF_2 cells with 100 μm path length. The IR spectra were accumulated for 32 scans, while the VCD spectra were recorded over a total measurement time of 4 hours each (~16000 scans). Background correction was carried out by subtraction of the solvent recorded under identical conditions.

Computational details.

Conformational searches for all investigated compounds were carried out systematically by preparing individual starting structures for unique combinations of torsional angles (threefold-rotation). All calculations were carried out using Gaussian 09 Rev. E.01[6] employing the B3LYP/6-31+G(2d,p)/IEFPCM($CHCl_3$) level of DFT. Spectra were simulated by assigning a uniform Lorentzian band shape of 6 cm^{-1} half-width at half-height to the computed dipole and rotational strength for the IR/VCD and a Gaussian shape of 0.3 eV width for the UV/ECD . The vibrational spectra presented in the main text are scaled with a frequency scaling factor σ of 0.98. Relative zero-point energy corrected energies, ΔE_{ZPC}, were used to determine Boltzmann weights. Note: Other DFT functionals such as M06-2X and B3LYP-GD3BJ were evaluated as well but gave worse conformational energies. This may be due to a further overestimation of C-H··π interactions (cf. main text for further discussion).

[6] Frisch, M. J.; Trucks, G. W.; Schlegel, H. B.; Scuseria, G. E.; Robb, M. A.; Cheeseman, J. R.; Scalmani, G.; Barone, V.; Mennucci, B.; Petersson, G. A.; Nakatsuji, H.; Caricato, M.; Li, X.; Hratchian, H. P.; Izmaylov, A. F.; Bloino, J.; Zheng, G.; Sonnenberg, J. L.; Hada, M.; Ehara, M.; Toyota, K.; Fukuda, R.; Hasegawa, J.; Ishida, M.; Nakajima, T.; Honda, Y.; Kitao, O.; Nakai, H.; Vreven, T.; J. A. Montgomery, J.; Peralta, J. E.; Ogliaro, F.; Bearpark, M.; Heyd, J. J.; Brothers, E.; Kudin, K. N.; Staroverov, V. N.; Keith, T.; Kobayashi, R.; Normand, J.; Raghavachari, K.; Rendell, A.; Burant, J. C.; Iyengar, S. S.; Tomasi, J.; Cossi, M.; Rega, N.; Millam, J. M.; Klene, M.; Knox, J. E.; Cross, J. B.; Bakken, V.; Adamo, C.; Jaramillo, J.; Gomperts, R.; Stratmann, R. E.; Yazyev, O.; Austin, A. J.; Cammi, R.; Pomelli, C.; Ochterski, J. W.; Martin, R. L.; Morokuma, K.; Zakrzewski, V. G.; Voth, G. A.; Salvador, P.; Dannenberg, J. J.; Dapprich, S.; Daniels, A. D.; Farkas, O.; Foresman, J. B.; Ortiz, J. V.; Cioslowski, J.; Fox, D. J. *Gaussian 09, Rev E.01*, Gaussian, Inc.: Wallingford CT, USA, 2013

7. Synthetic procedures

General procedure for the synthesis of chiral alkyl-phenyl ethers 4b to 4d

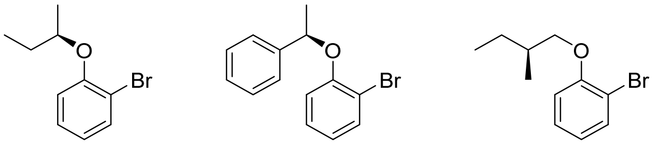

4b **4c** **4d**

In a heat dried schlenk tube, 2-bromophenol (519.0 mg, 3.0 mmol, 1.5 eq.) triphenylphosphine (524.6 mg, 2.0 mmol, 1 eq.) and the chiral alcohol (2.0 mmol, 1 eq.) were mixed under argon atmosphere. Dry THF (3 mL) was added and the mixture was cooled to 0 °C, before DIAD (0.39 mL, 404.4 mg, 2.0 mmol, 1 eq.) was added dropwise *via* syringe. The mixture was then allowed to warm to r.t. and stirred for another 0.5 h, before the schlenk tube was fitted with a reflux condenser and the reaction mixture was stirred under reflux. After completion of the reaction, as judged by TLC, the solvent was removed, yielding a yellow oil. The crude product was purified by column chromatography (eluent: cyclohexane) to afford **4b** to **4d** as colourless, viscous liquids.

(*R*)-4b: 274.9 mg, 1.2 mmol, 60%; **(*S*)-4b**: 252.0 mg, 1.1 mmol, 55%
^{1}H-NMR (200 MHz, $CDCl_3$): δ = 7.55-7.51 (m, 1H, CH-ar), 7.27-7.18 (m, 1H, C*H*-ar), 6.92-6.76 (m, 2H, C*H*-ar), 4.34 (sext, ^3J(H,H) = 6.02 Hz, 1H, C*H*), 1.92-1.58 (m, 2H, CH_2), 1.34 (d, ^3J(H,H) = 6.11 Hz, 3H, CH_3), 1.02 (t, ^3J(H,H) = 7.43 Hz, 3H, CH_3) ppm.

(*R*)-4c: 249.6 mg, 0.9 mmol, 45% **(*S*)-4c**: 332.3 mg, 1.2 mmol, 60%
^{1}H-NMR (200 MHz, $CDCl_3$): δ = 7.54-7.50 (m, 1H, C*H*-ar), 7.43-7.23 (m, 5H, C*H*-ar), 7.12-7.05 (m, 1H, C*H*-ar), 6.80-6.73 (m, 2H, C*H*-ar), 5.36 (q, ^3J(H,H) = 6.43 Hz, 1H, C*H*), 1.69 (d, ^3J(H,H) = 6.43 Hz, 3H, CH_3) ppm.

(*S*)-4d: 170.4 mg, 0.7 mmol, 35%
^{1}H-NMR (200 MHz, $CDCl_3$): δ = 7.55-7.50 (dd, 1H, C*H*-ar), 7.28-7.20 (m, 1H, C*H*-ar), 6.90-6.76 (m, 2H, C*H*-ar), 3.93-3.76 (dq, ^3J(H,H) = 9.56 Hz, ^4J(H,H) = 5.85 Hz, 2H, O-CH_2), 1.94 (oct, ^3J(H,H) = 6.74 Hz, 1H, C*H*), 1.73-1.55 (m, 1H, CH_2-CH_3), 1.40-1.22 (m, 1H, CH_2-CH_3), 1.07 (d, ^3J(H,H) = 6.74 Hz, 3H, CH-CH_3), 0.96 (t, ^3J(H,H) = 7.39 Hz, 3H, CH_2-CH_3) ppm.

Tris-(*o*-alkoxyphenyl)-borane ammonia complexes 1b to 1d

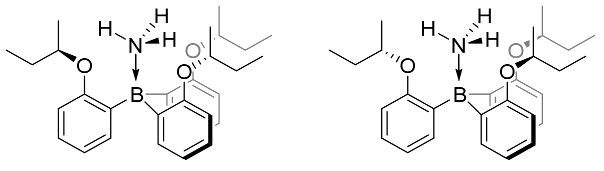

(*R*,*R*,*R*)-1b **(*S*,*S*,*S*)-1b**

Tris-(*o*-2-butoxyphenyl)-borane ammonia complex 1b

In a heat dried schlenk flask, the 2-butylphenylether **4b** (450.0 mg, 1.96 mmol, 3 eq.) was dissolved in dry diethyl ether (5 ml) under argon atmosphere. The mixture was cooled to 0 °C and a solution of *n*-butyl lithium in hexanes (2.39 mol/L, 0.82 mL, 1.96 mmol, 3 eq.) was added dropwise. After complete addition, the mixture was allowed to warm to r.t. and was stirred for 1 h at that temperature. Subsequently, the mixture was again cooled to 0 °C before $BF_3 \times Et_2O$ (80.0 µL, 92.0 mg, 0.65 mmol, 1 eq.) was added dropwise. The mixture was stirred an additional 16 h at r.t. Quenching with a saturated, aqueous solution of ammonia afforded a white precipitate, which was re-dissolved by adding diethyl ether. The aqueous phase was then separated and extracted with diethyl ether (3 × 10 mL) and the combined organic phases were dried with anhydrous magnesium sulfate. After removal of the solvent, a pale yellow oil was obtained. The crude product was purified by column chromatography on silica (eluent: cyclohexane/ethylactetate: 20/1) to afford a slightly yellow solid, which was recrystallized from ethanol to give **1b** as colourless crystals.

(*R*,*R*,*R*)-1b: 21.8 mg, 45.8 µmol, 7%; **(*S*,*S*,*S*)-1b**: 18.5 mg, 39.0 µmol, 6%

^{1}H-NMR (250 MHz, $CDCl_3$): δ = 7.34-7.04 (m, 3H, C*H*-ar), 6.79-6.66 (m, 9H, C*H*-ar), 5.66 (s_{br}, 3H, N*H*), 4.34 (sext, ^{3}J(H,H) = 5.96 Hz, 3H, O-C*H*), 1.53-1.22 (m, 6H, C*H*$_2$), 1.01 (s, 9H, C*H*$_3$), 0.75 (s, 9H, C*H*$_3$) ppm.

^{13}C-NMR (62.5 MHz, $CDCl_3$): δ = 160.38, 137.09, 126.15, 119.99, 111.18, 73.75, 29.38, 9.48 ppm.

^{11}B-NMR (80 MHz, $CDCl_3$): δ = -3.55 ppm.

MS (EI, 70 eV): cal. for $C_{30}H_{39}BO_3$ [$M^{+\cdot}$-NH_3] *m*/*z* = 458.2998, found *m*/*z* = 458.1985.

Tris-(*o*-2-phenylethoxyphenyl)-borane ammonia complex 1c

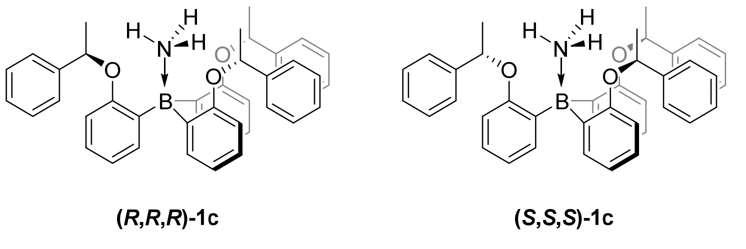

(*R*,*R*,*R*)-1c **(*S*,*S*,*S*)-1c**

In a heat dried schlenk flask, the 2-benzylphenylether **4c** (544.3 mg, 1,96 mmol, 3 eq.) was dissolved in dry diethyl ether (5 ml) under argon atmosphere. The mixture was cooled to 0 °C and a solution of *n*-butyl lithium in hexanes (2.39 mol/L, 0.82 mL, 1.96 mmol, 3 eq.) was added dropwise. After complete addition, the mixture was allowed to warm to r.t. and was stirred for 1 h at that temperature. Subsequently, the mixture was again

cooled to 0 °C before $BF_3 \times Et_2O$ (80.0 µL, 92.0 mg, 0.65 mmol, 1 eq.) was added dropwise. The mixture was stirred an additional 16 h at r.t. Quenching with a saturated, aqueous solution of ammonia afforded a white precipitate, which was re-dissolved by adding diethyl ether. The aqueous phase was then separated and extracted with diethyl ether (3 × 10 mL) and the combined organic phases were dried with anhydrous magnesium sulfate. After removal of the solvent, a pale yellow oil was obtained. The crude product was purified by column chromatography on silica (eluent: cyclohexane/ethylacetate: 20/1) to afford a slightly yellow solid which was washed with ethanol to give **1c** as a colourless solid.

(*R*,*R*,*R*)-1c: 8.0 mg, 13 µmol 2%; **(*S*,*S*,*S*)-1c**: 12.3 mg, 19.9 µmol 3%

^{1}H-NMR (250 MHz, CDCl$_3$): δ = 7.37-7.28 (m, 3H, C*H*-ar), 7.24-7.18 (m, 6H, C*H*-ar), 7.15-7.03 (m, 9H, C*H*-ar), 6.90-6.71 (m, 9H, C*H*-ar), 5.54 (s_{br}, 3H, NH_3), 5.20 (q, ^3J(H,H) = 6.44 Hz, 3H, O-C*H*), 1.23 (d, ^3J(H,H) = 6.44 Hz, 9H, CH_3) ppm.

^{13}C-NMR (62.5 MHz, CDCl$_3$): δ = 160.91, 144.32, 137.15, 128.71, 127.42, 126.77, 125.71, 120.81, 112.57, 23.64 ppm.

^{11}B-NMR (80 MHz, CDCl$_3$): δ = -2.34 ppm.

MS (EI, 70 eV): cal. for $C_{42}H_{37}BO_3$ [$M^{+\cdot}$-NH_3] *m*/*z* = 601.3029, found *m*/*z* = 601.8820.

Tris-(*o*-2-methylbutoxyphenyl)-borane ammonia complex 1d

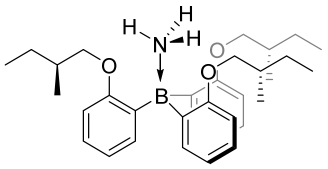

(*S*,*S*,*S*)-1d

In a heat dried three-neck flask, magnesium turnings (60.0 mg, 2.47 mmol, 4 eq.) were suspended in dry diethyl ether (10 mL) under argon atmosphere. A solution of 2-methylbutylphenylether **4d** (450.0 mg, 1.82 mmol, 3 eq.) in diethyl ether (5 mL) was slowly added upon which the ether started to reflux gently. Additional ether (10 mL) was added and the mixture was refluxed for 6 h, until no more magnesium turnings were observed in the reaction flask. The mixture was cooled down to 0 °C and $BF_3 \times Et_2O$ (98.0 µL, 87.6 mg, 0.61 mmol, 1 eq.) was added. The mixture was stirred for additional 16 h at r.t. and subsequently quenched with a saturated aqueous solution of ammonia. The resulting white precipitate was re-dissolved in diethyl ether and the aqueous phase was separated and extracted with diethyl ether (3 × 10 mL). The combined organic phases were dried with anhydrous sodium sulfate. Removal of the solvent gave a yellowish oil. The crude product was purified by column chromatography on silica (eluent: hexane/ethylacetate: 10/1) to afford a slightly yellow solid which was crystallized from ethanol to give **1c** as a colourless crystalline needles (18.9 mg, 36.5 µmol, 6%).

^{1}H-NMR (250 MHz, CDCl$_3$): δ = 7.16-7.07 (m, 3H, C*H*-ar), 6.82-6.66 (m, 9H, C*H*-ar), 5.59 (s_{br}, 3H, NH_3), 4.00-3.34 (m, 6H, O-CH_2), 1.63-1.41 (m, 3H, C*H*), 1.28-0.85 (m, 6H, CH_2-CH_3), 0.77-0.66 (m, 18H, CH_2-CH_3, CH-CH_3) ppm.

^{13}C-NMR (62.5 MHz, CDCl$_3$): δ = 162.08, 136.64, 126.66, 120.71, 110.81, 72.98, 34.98, 26.23, 16.66, 11.38 ppm.

^{11}B-NMR (80 MHz, CDCl$_3$): δ = -2.91 ppm.

MS (EI, 70 eV): cal. for $C_{33}H_{45}BO_3$ [$M^{+\cdot}$-NH_3] *m*/*z* = 500.3462, found *m*/*z* = 500.3827.

^{1}H-NMR 4b

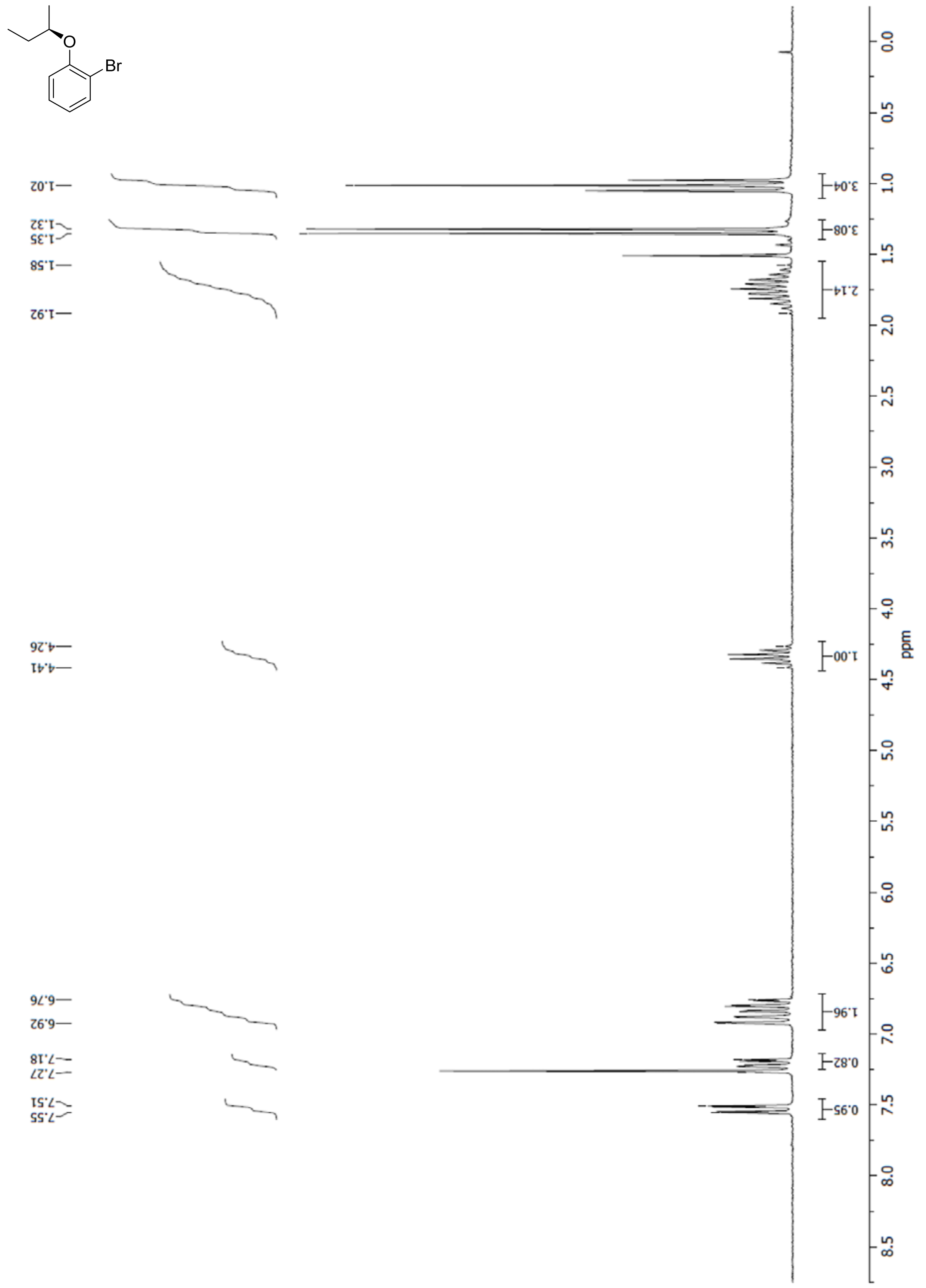
O
Br
1.02
1.32
1.35
1.58
1.92
4.26
4.41
6.76
6.92
7.18
7.27
7.51
7.55
3.04
3.08
2.14
1.00
1.96
0.82
0.95
ppm

^{1}H-NMR 4c

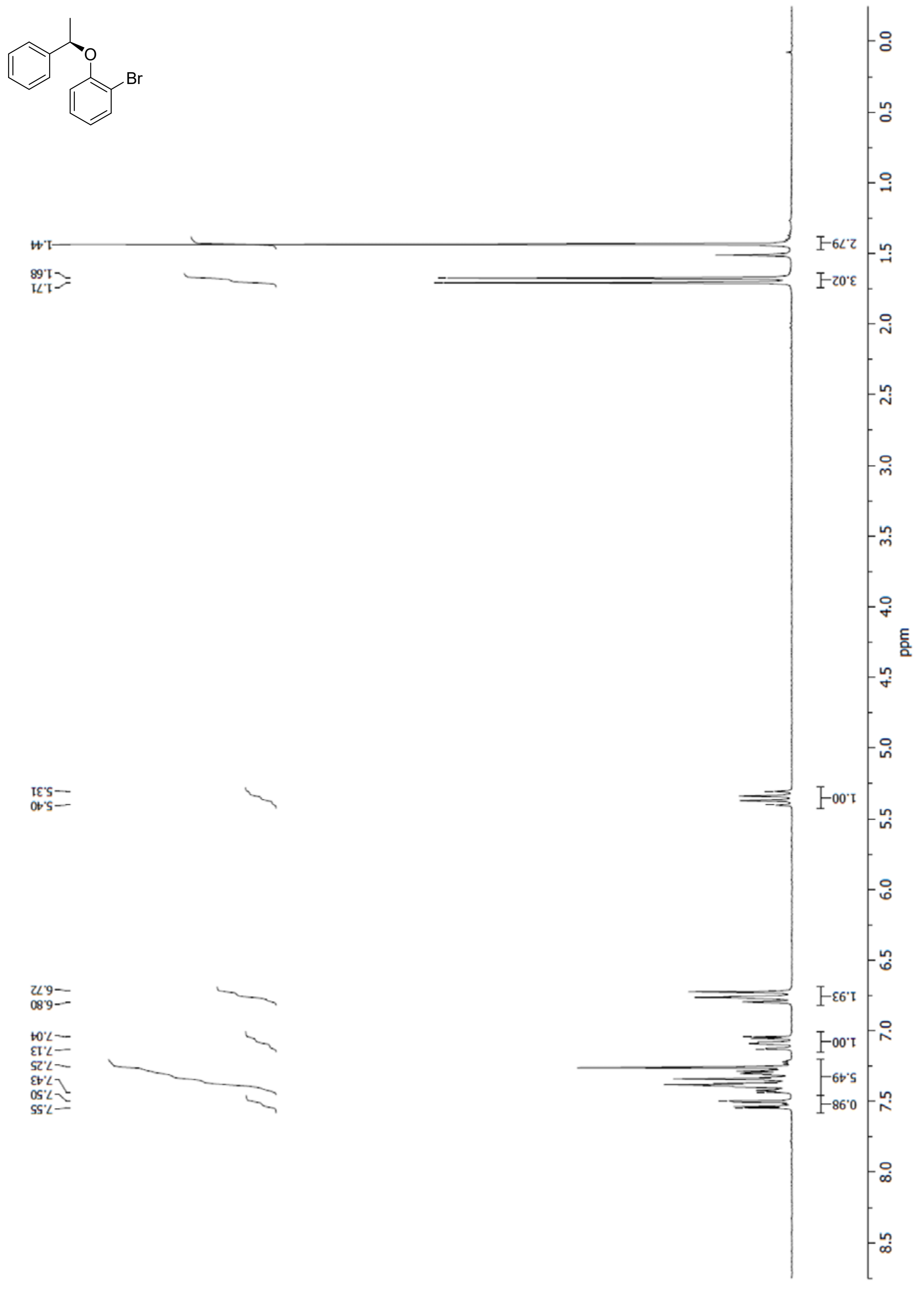

^{1}H-NMR 4d

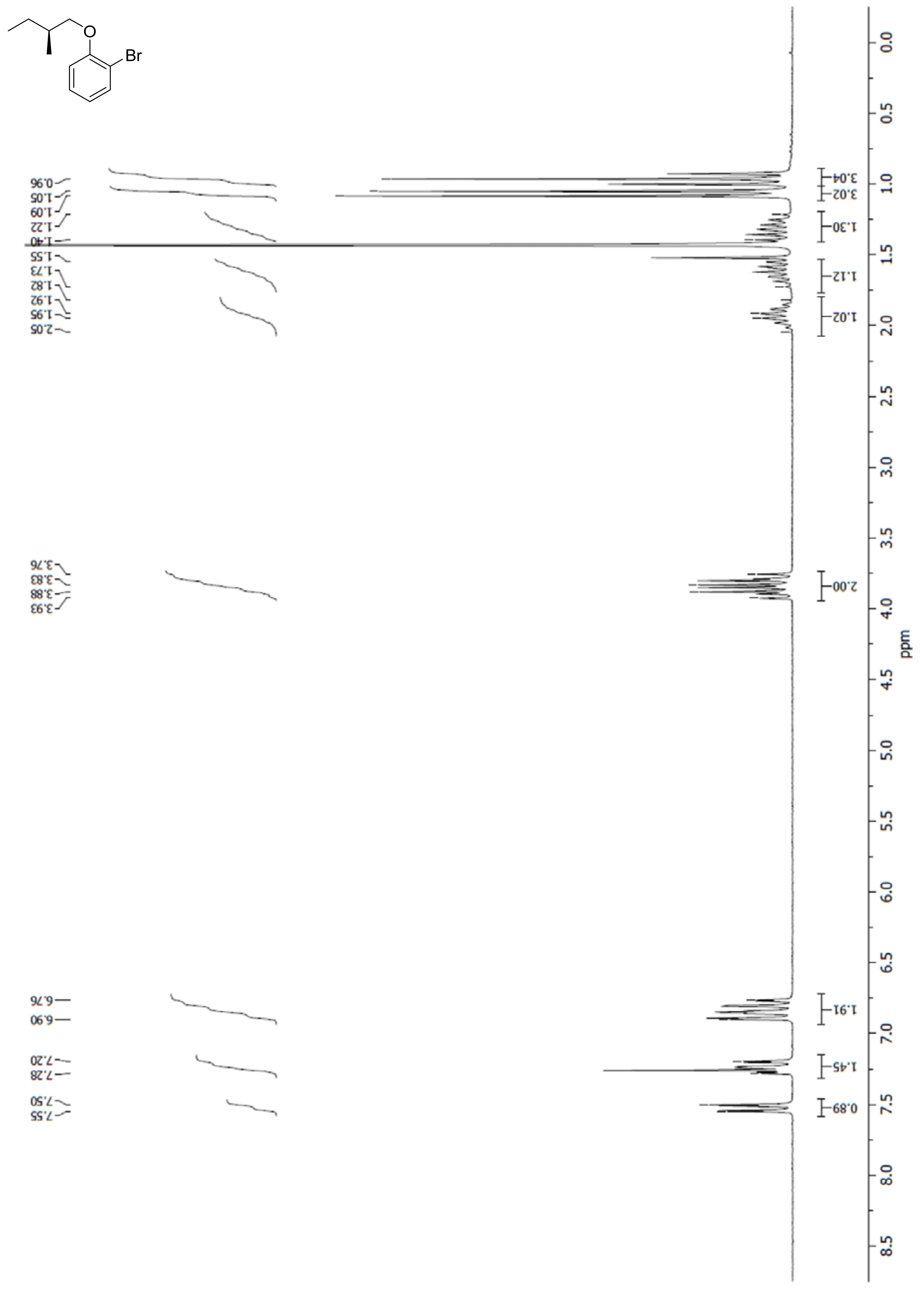

^{1}H-NMR 1b

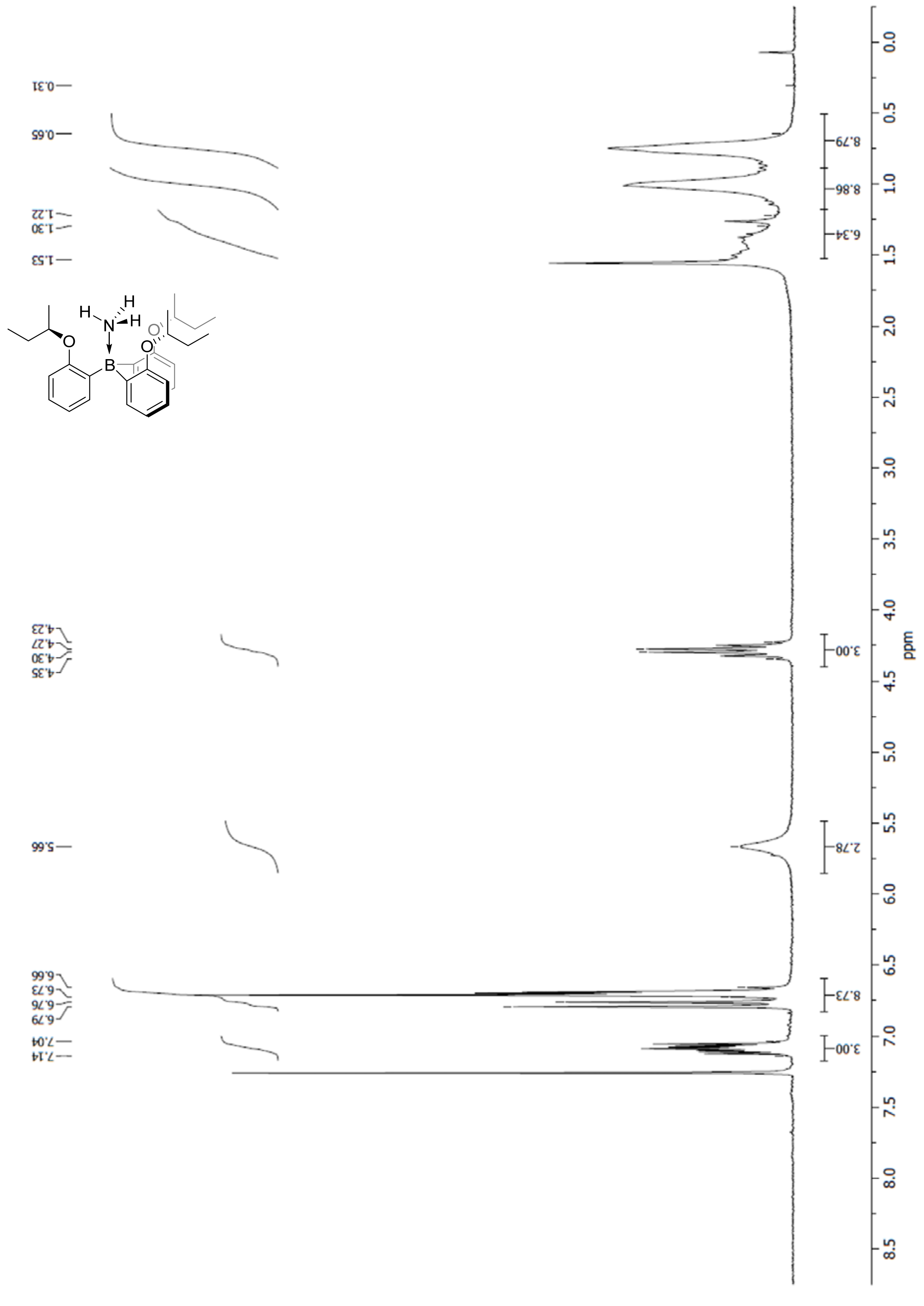

0.31
0.65
1.22
1.30
1.53
4.23
4.27
4.30
4.35
5.66
6.66
6.73
6.76
6.79
7.04
7.14
8.79
8.86
6.34
3.00
2.78
8.73
3.00
ppm

^{13}C-NMR 1b

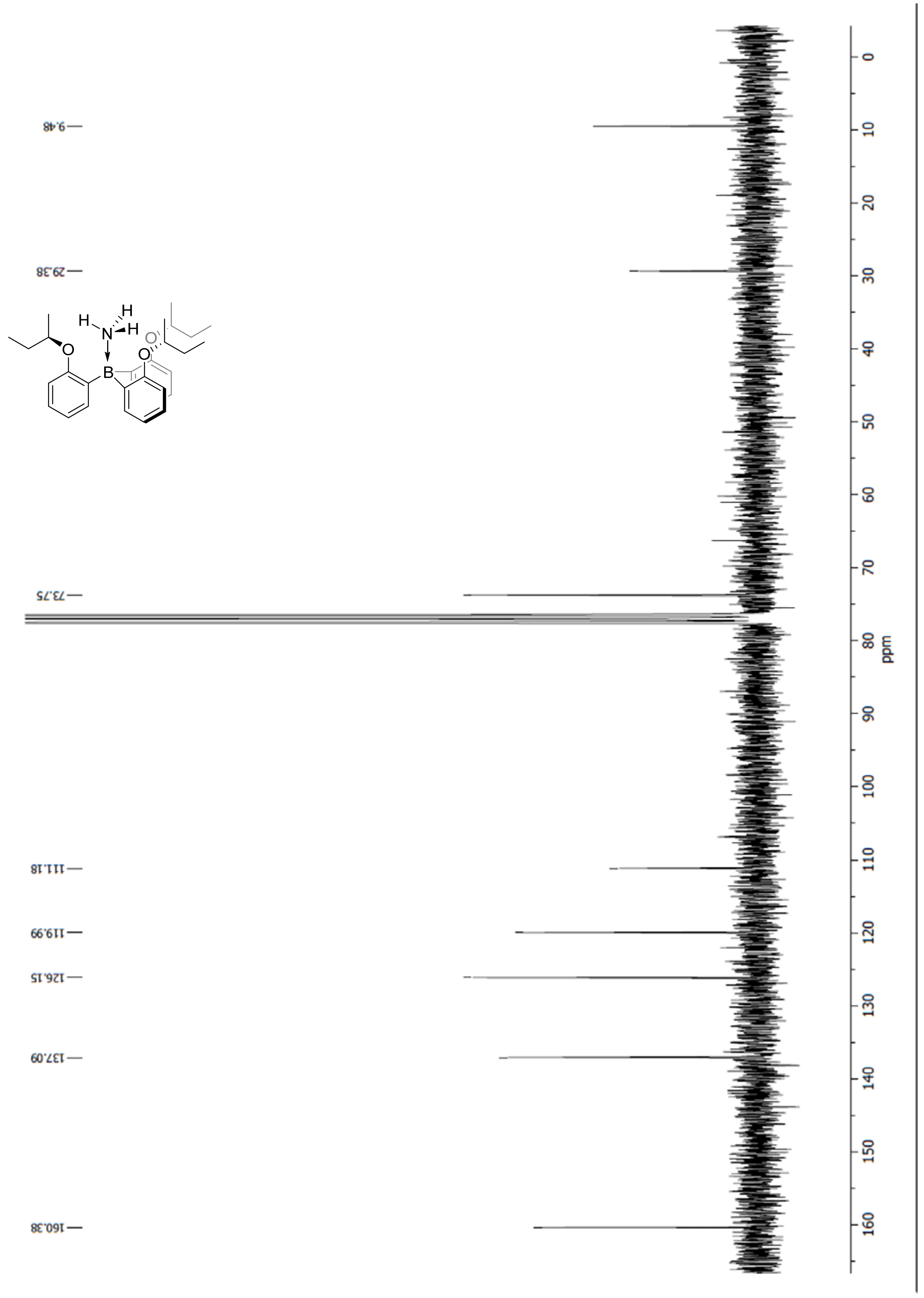

^{11}B-NMR 1b

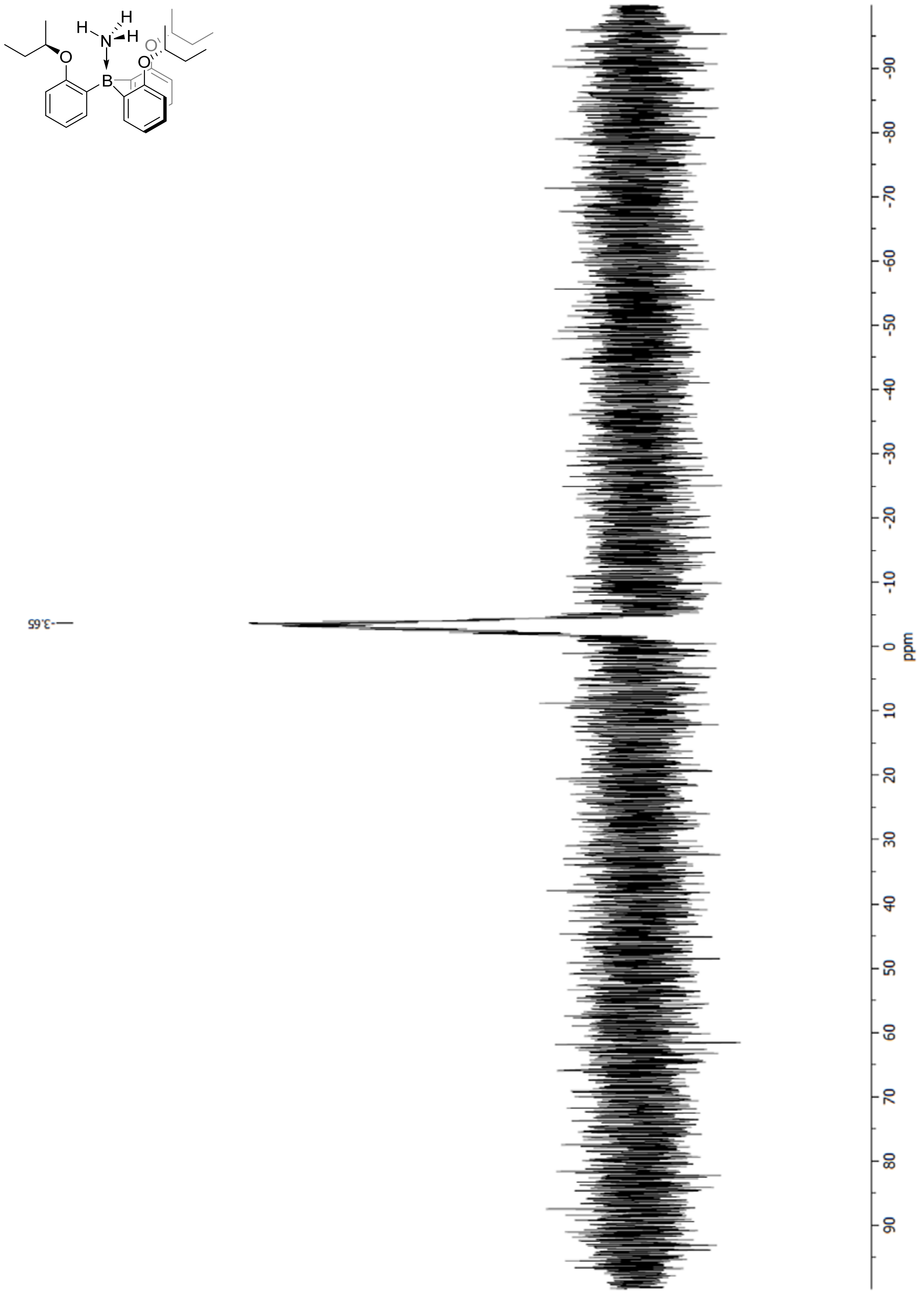

^{1}H-NMR 1c

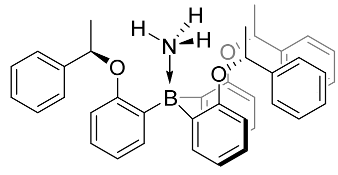
H
N
H
H
O
B
O
O

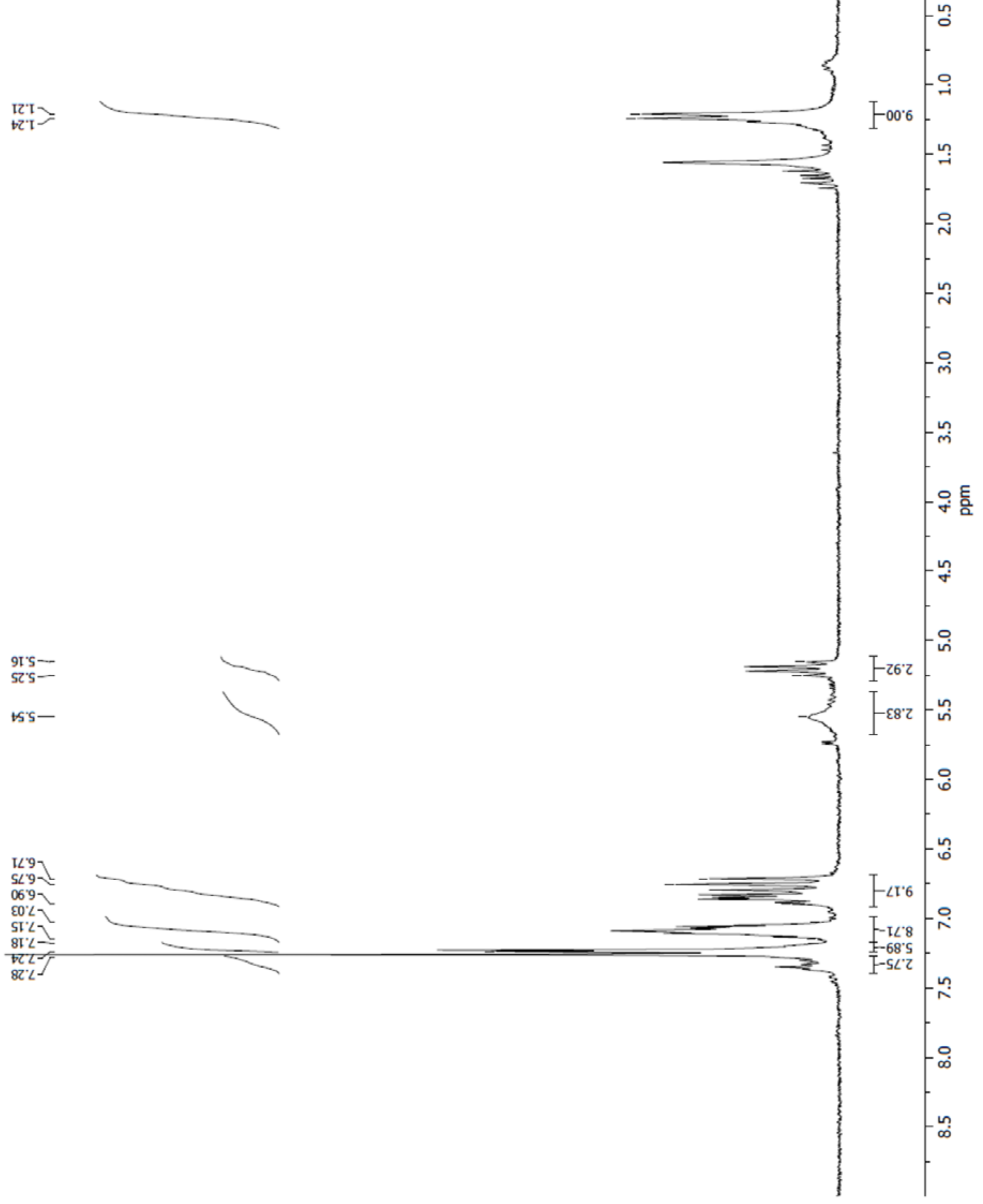
1.21
1.24
5.16
5.25
5.54
6.71
6.75
6.90
7.03
7.15
7.18
7.24
7.28
9.00
2.92
2.83
9.17
8.71
5.89
2.75
ppm

^{13}C-NMR 1c

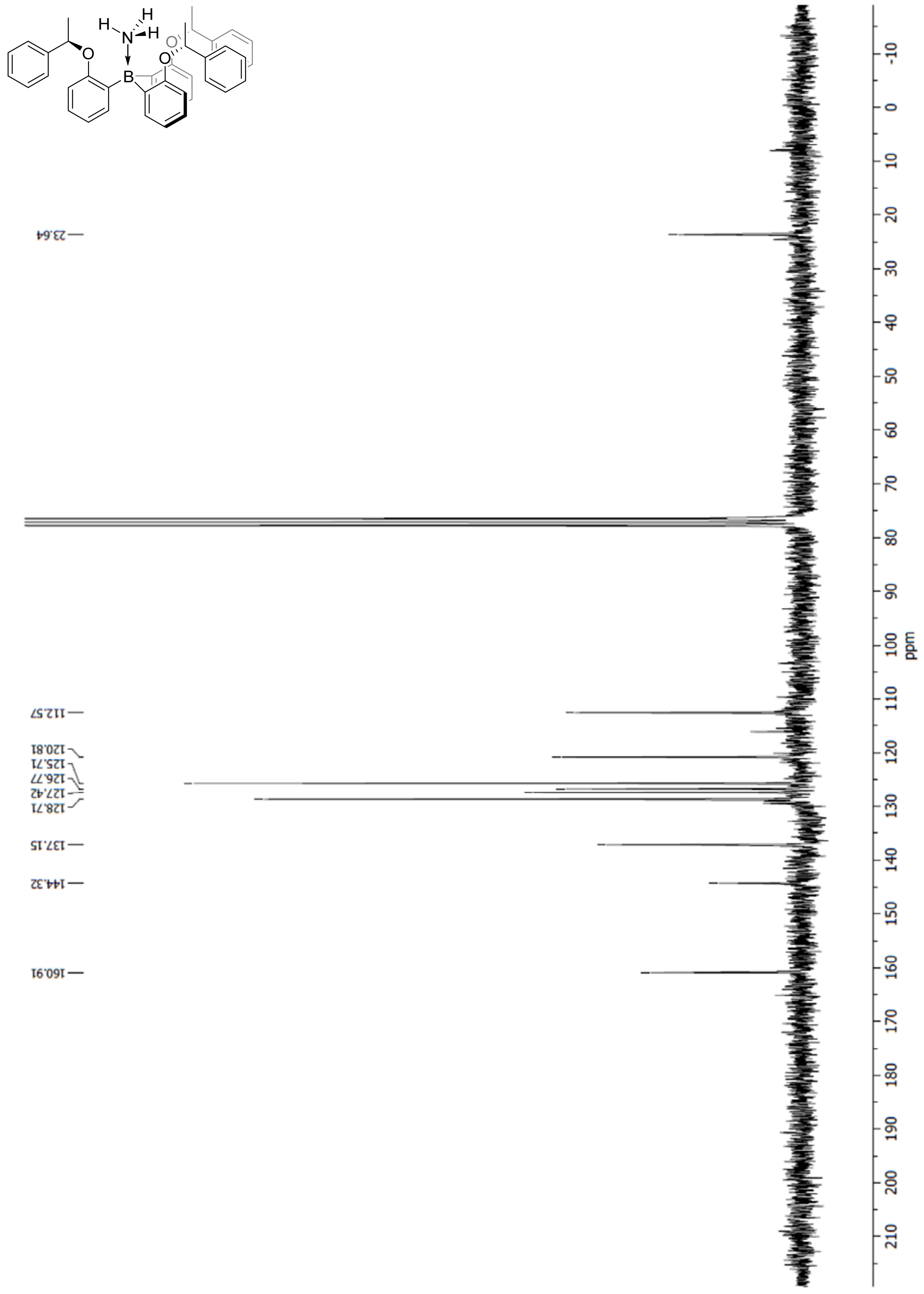
23.64
112.57
120.81
125.71
126.77
127.42
128.71
137.15
144.32
160.91
ppm

^{11}B-NMR 1c

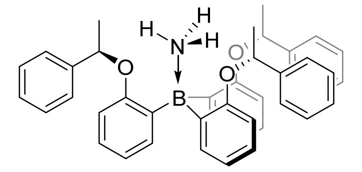

H
N
H
H
O
B
O
O

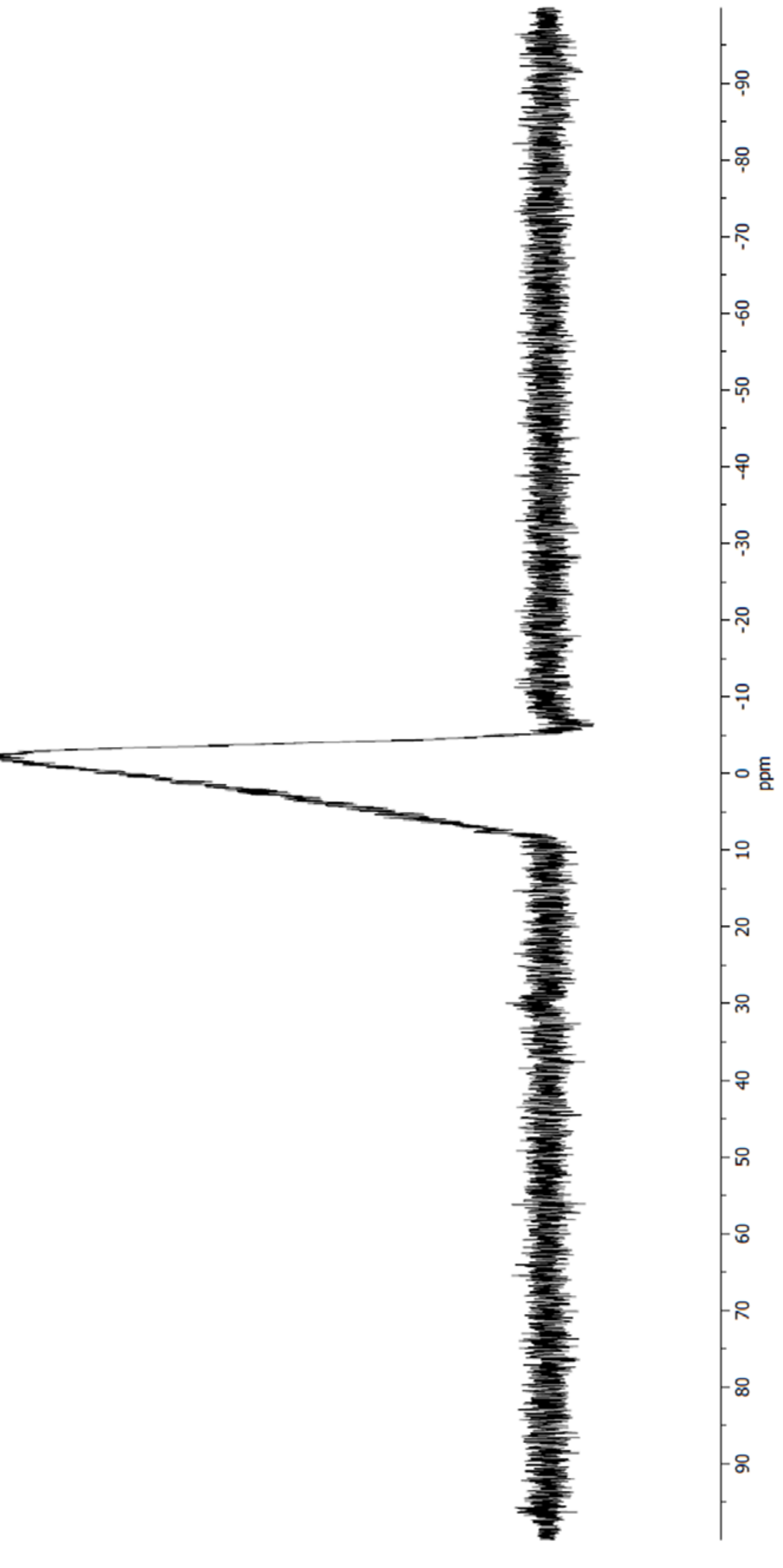

-2.34
ppm
-90
-80
-70
-60
-50
-40
-30
-20
-10
0
10
20
30
40
50
60
70
80
90

^{1}H-NMR 1d

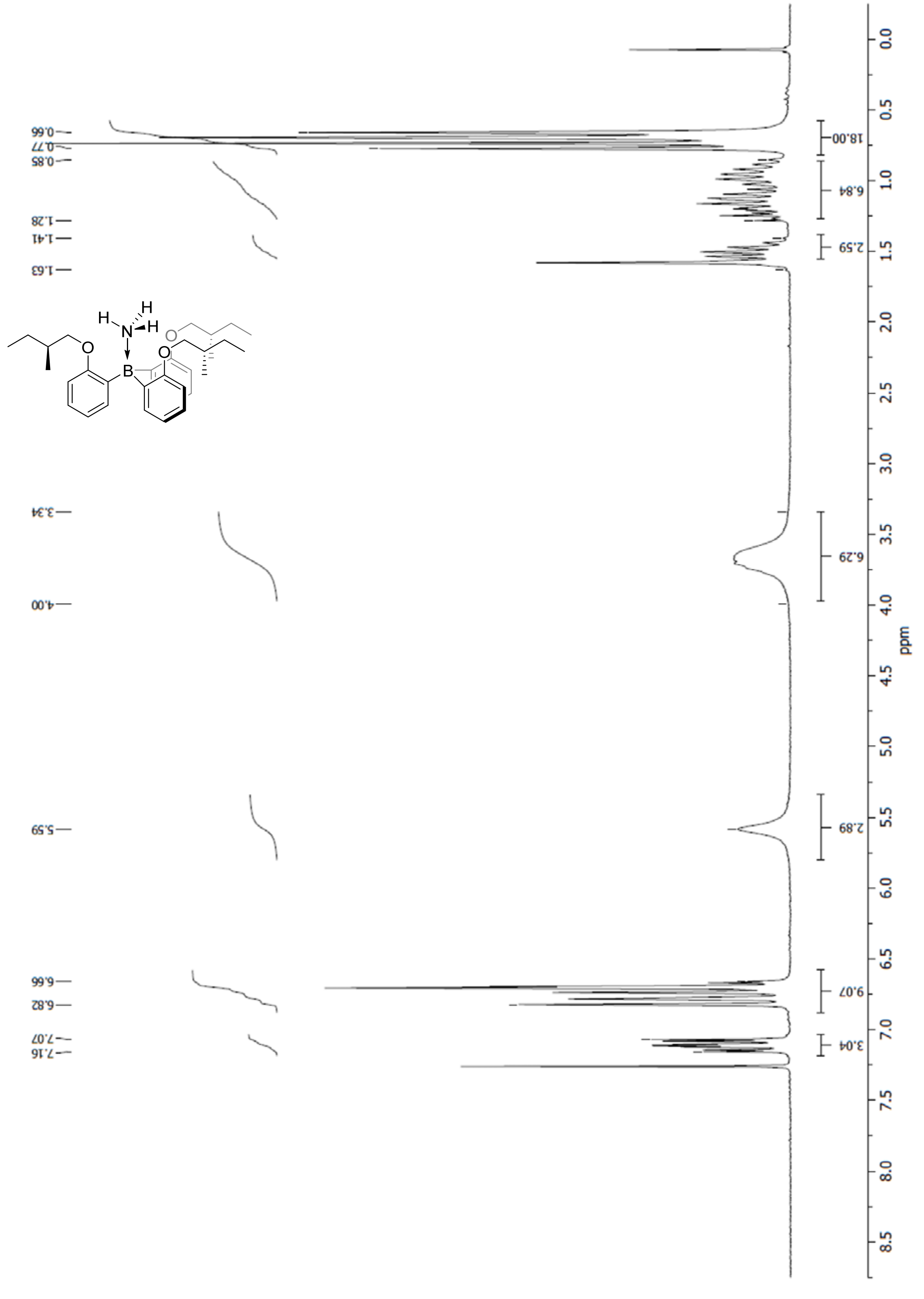

0.66
0.77
0.85
1.28
1.41
1.63
3.34
4.00
5.59
6.66
6.82
7.07
7.16
18.00
6.84
2.59
6.29
2.89
9.07
3.04
ppm

^{13}C-NMR 1d

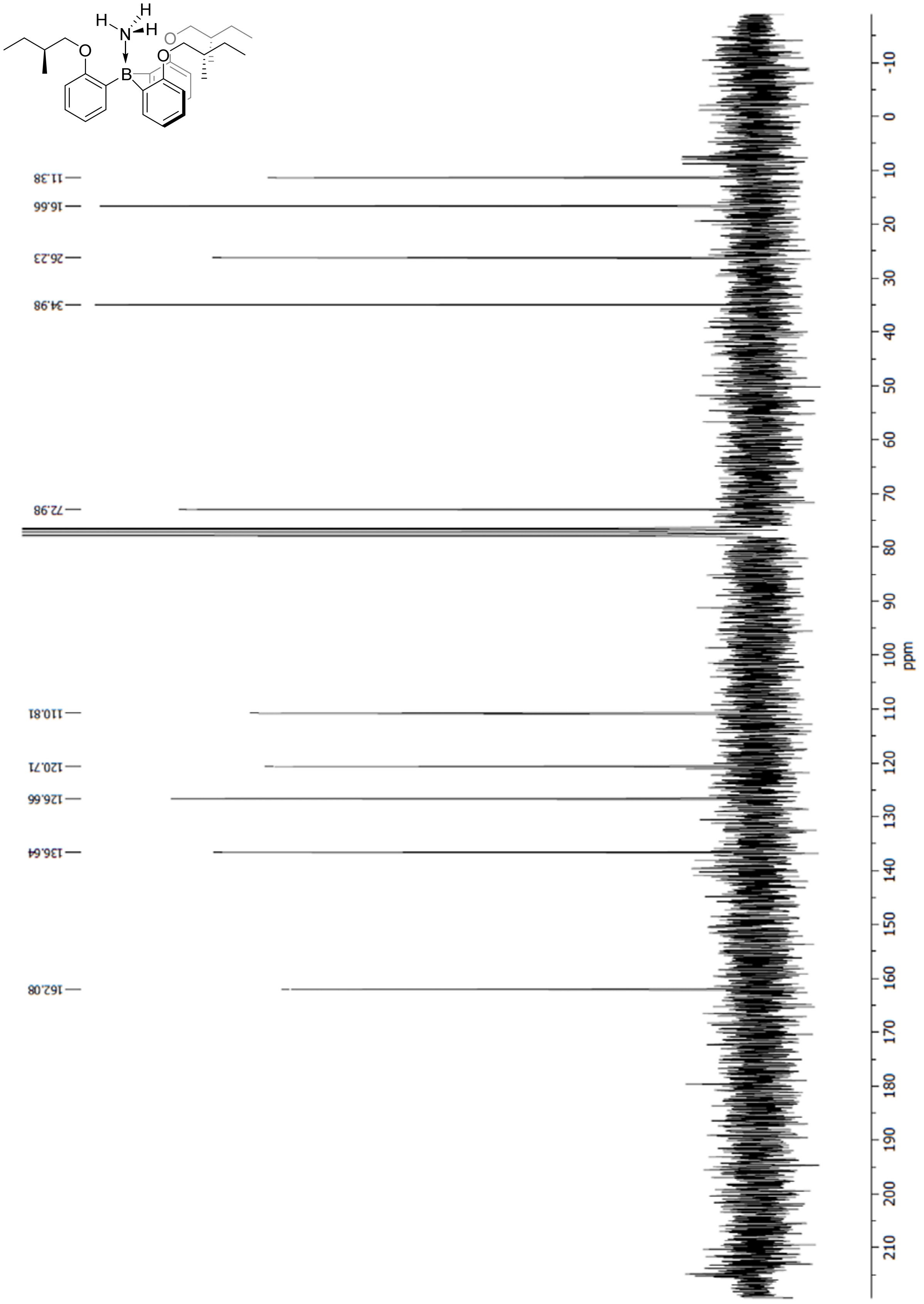

^{11}B-NMR 1d

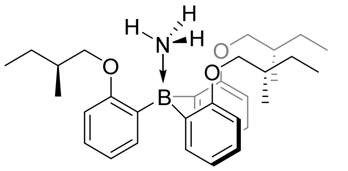
H
H
N
H
O
B
O
O

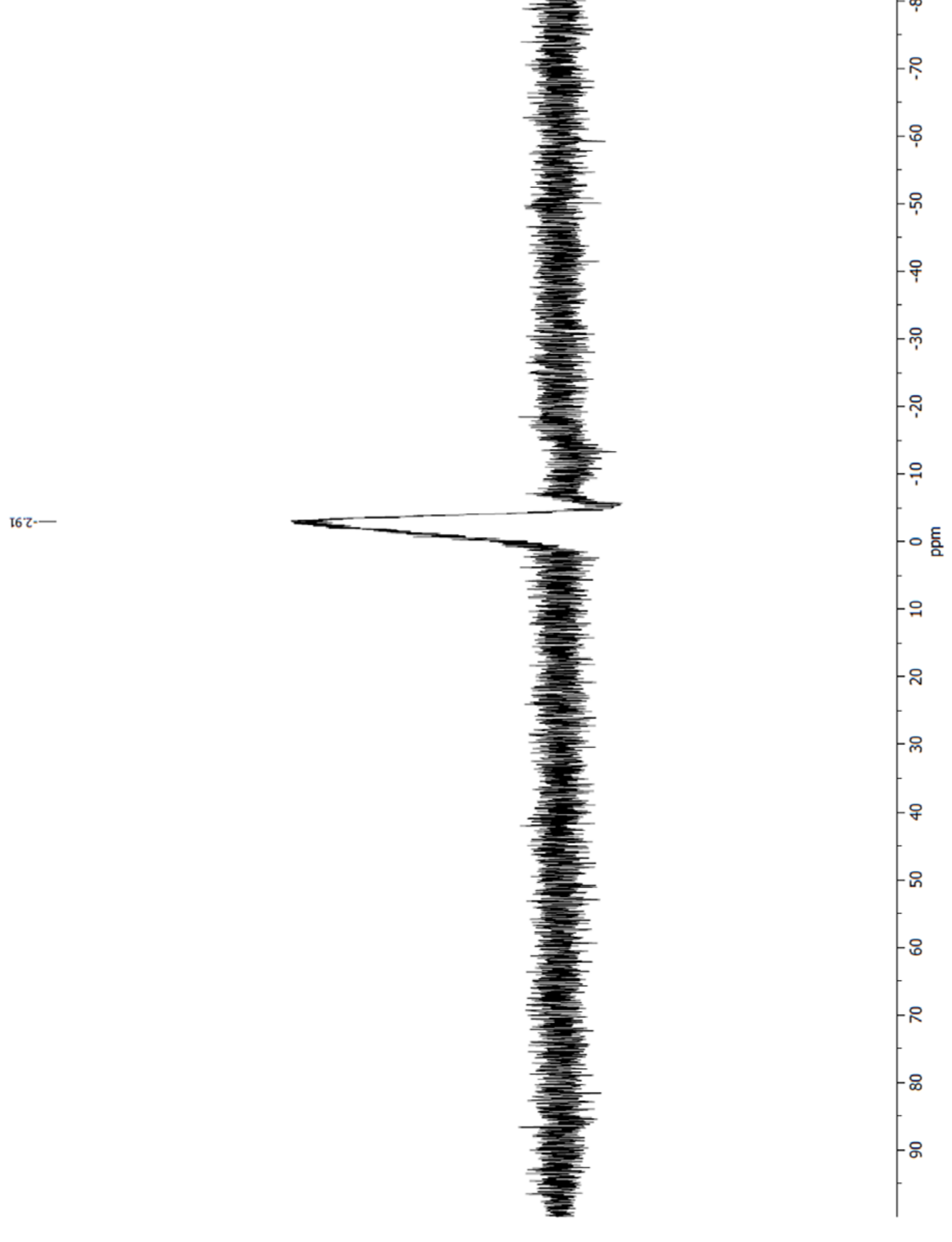
-2.91
-90
-80
-70
-60
-50
-40
-30
-20
-10
0
10
20
30
40
50
60
70
80
90
ppm

8. Selected Cartesian coordinates from computations

Conformers of 1b

1b-c1

B	0.01173700	-0.00498200	-1.02233600
C	-1.00814700	1.18669600	-1.51396000
C	-0.66049400	2.02475900	-2.58352600
C	-2.29265800	1.40738200	-0.95641100
C	-1.50080500	3.02148700	-3.08911000
H	0.31274000	1.88732500	-3.04480000
C	-3.14349600	2.41228800	-1.43252800
C	-2.74686100	3.21563100	-2.50415600
H	-1.17886200	3.63784100	-3.92299800
H	-4.11467200	2.58219800	-0.98655000
H	-3.41799400	3.98836700	-2.86734900
O	-2.65976400	0.56252900	0.07500800
C	-3.94316500	0.67454900	0.72474000
H	-4.70183300	0.89038800	-0.03691900
C	-3.91825400	1.78243900	1.77717000
H	-3.21129900	1.53199500	2.57604000
H	-4.91101300	1.90006400	2.22401700
H	-3.62318000	2.74291800	1.34680200
C	-4.24842400	-0.69506900	1.33366700
H	-5.14777700	-0.58539900	1.95200600
H	-3.43257800	-0.96085200	2.01908600
C	-4.45969300	-1.80720900	0.30283100
H	-4.67914200	-2.75854600	0.79884300
H	-3.57328500	-1.94621500	-0.32227300
H	-5.30389200	-1.57465000	-0.35713400
N	0.01498300	0.01293400	0.60795200
H	-0.94746900	0.06357300	0.94383500
H	0.45610600	-0.84127800	0.95093800
H	0.53892800	0.82656300	0.93231600
C	-0.51789400	-1.49009900	-1.48737100
C	-0.07704100	-2.70661200	-0.90859900
C	-1.41722300	-1.61957900	-2.55590700
C	-0.53399100	-3.95025200	-1.36156800
C	-1.87060800	-2.85043500	-3.03987900
H	-1.77691100	-0.71343100	-3.03381000
C	-1.42778200	-4.01985800	-2.43261300
H	-0.20502100	-4.87116600	-0.89830700
H	-2.56447600	-2.88857700	-3.87415000
H	-1.77047400	-4.99080200	-2.77797000
C	1.55608100	0.27193400	-1.51264900
C	2.39901200	1.26768700	-0.95832500
C	2.10094600	-0.45520500	-2.58128400
C	3.69710200	1.48800000	-1.43488800
C	3.38634000	-0.23966300	-3.08753500
H	1.48801300	-1.22445200	-3.04099200
C	4.18733400	0.73563000	-2.50469800
H	4.33739700	2.23875800	-0.99070300
H	3.75303300	-0.83209200	-3.92028200
H	5.19411400	0.91955800	-2.86801500
O	1.85848700	2.01783700	0.07035500
O	0.84178000	-2.59296200	0.11905900
C	1.37266900	-3.75386200	0.79151500
H	1.55385000	-4.53674100	0.04554700
C	2.60894200	3.06663700	0.71754200
H	3.18135700	3.60744900	-0.04542300
C	0.39584000	-4.25145400	1.85622600
H	0.77918300	-5.16523800	2.32218700
H	-0.58678800	-4.47272900	1.43159400
H	0.26958400	-3.49533800	2.63922000
C	2.71811800	-3.33805800	1.38856200
H	3.06525800	-4.16385500	2.02157400
H	2.55251300	-2.48374400	2.05839900
C	3.54927800	2.48366300	1.77174900
H	4.15811000	3.27883000	2.21458700
H	4.22369000	1.73737400	1.34397000
H	2.97367300	2.00734900	2.57325800
C	1.58552300	4.02885300	1.32283800
H	2.13730100	4.75047700	1.93762300
H	0.94175100	3.46494200	2.01104000
C	3.78705800	-2.99768600	0.34661600
H	3.99905100	-3.86198900	-0.29392800
H	4.72433400	-2.70760000	0.83293400
H	3.46832700	-2.17318000	-0.29717800
C	0.73563600	4.77038400	0.28760400
H	1.36473200	5.38016300	-0.37167300
H	0.02273500	5.44077000	0.77901000
H	0.17168700	4.07271600	-0.33770200

1b-c2

B	-0.07121500	-0.00827500	0.48220000
C	1.10068000	-1.07891400	0.05412300
C	1.98204500	-0.78889400	-0.99818200
C	1.26418200	-2.35224700	0.65385700
C	2.96443600	-1.67512200	-1.44988000
H	1.89007900	0.17414900	-1.49097700
C	2.24601600	-3.25303100	0.22478400
C	3.09574200	-2.91284800	-0.82971600
H	3.61382900	-1.39806500	-2.27481900
H	2.35844300	-4.22190500	0.69419100
H	3.85035500	-3.62213000	-1.15673900
O	0.41387000	-2.64666600	1.70559800
C	0.26906700	-3.99317500	2.20625600
H	1.26534600	-4.42949900	2.34851500
C	-0.54458100	-4.84185200	1.23182300
H	-1.55790400	-4.43968100	1.13246900
H	-0.61221900	-5.87138500	1.59867800
H	-0.08571000	-4.86427700	0.23998700
C	-0.39991300	-3.87829200	3.57674600
H	-0.66825900	-4.89202500	3.89681800
H	-1.34361000	-3.33054400	3.45791400
C	0.47149300	-3.22106800	4.65145000
H	-0.06011300	-3.17240700	5.60734100
H	0.75708900	-2.20066900	4.37662400
H	1.39452100	-3.79023400	4.81193700
N	-0.08680600	0.05237600	2.10985200
H	0.74615800	0.54433900	2.43418300
H	-0.92374100	0.54839000	2.41749800
H	-0.09246400	-0.89844900	2.48004000
C	0.26378800	1.51058300	-0.05028700
C	1.30003100	2.32121400	0.47621100
C	-0.44877500	2.06320400	-1.12516100
C	1.58667000	3.58856200	-0.04451500
C	-0.17692700	3.32339800	-1.66618800
H	-1.24972800	1.47566300	-1.56335700
C	0.84691700	4.08832900	-1.11821700
H	2.38293700	4.19475700	0.36795400
H	-0.75808900	3.69604600	-2.50426900
H	1.08188800	5.07106400	-1.51631900
C	-1.56252200	-0.51012300	0.00532800
C	-2.77493600	0.00468900	0.52801900
C	-1.70103100	-1.46084500	-1.01720600
C	-4.02367300	-0.41662400	0.05604400
C	-2.93643800	-1.89034100	-1.51107500
H	-0.79841300	-1.87975200	-1.45152100
C	-4.10273700	-1.36453300	-0.96615000
H	-4.94069900	-0.01363500	0.46619200
H	-2.98109400	-2.62410000	-2.31019900
H	-5.07732400	-1.67935100	-1.32743000
O	-2.65621200	0.93245200	1.54828500
O	1.99825200	1.79940700	1.55123300
C	3.24531600	2.37597700	1.99519200
H	3.13020500	3.46465900	2.06304400
C	-3.77873200	1.73328900	1.97639900
H	-4.65124800	1.08161500	2.10799700
C	4.37230100	2.02311700	1.02708100
H	5.30418800	2.50083200	1.34724700
H	4.14896100	2.36098100	0.01179600
H	4.52621400	0.93945000	1.00247300
C	3.49786900	1.83578100	3.40356200
H	4.51649500	2.12462100	3.68827700
H	3.48666600	0.73901700	3.36214000

C	-4.08249600	2.82148600	0.94905400
H	-4.96203000	3.39486700	1.26010300
H	-4.28568200	2.39622800	-0.03712700
H	-3.23353800	3.50671900	0.85878900
C	-3.40619500	2.31076600	3.34274800
H	-4.17043700	3.05190300	3.60491500
H	-2.46209800	2.86144200	3.24181300
C	2.51248500	2.34400700	4.46037600
H	2.55410300	3.43621900	4.54416300
H	2.74695400	1.92538100	5.44449000
H	1.48021500	2.07102100	4.21936700
C	-3.30458900	1.26992900	4.46200700
H	-4.25806500	0.74713900	4.60068200
H	-3.04416100	1.74563800	5.41309800
H	-2.54238100	0.51391400	4.24824700

1b-c3

B	-0.12565600	0.04184400	-0.55136500
C	0.41980700	1.48877000	-1.10781500
C	1.33312000	1.54075200	-2.17102700
C	-0.02225300	2.74396500	-0.61966900
C	1.79719000	2.73370000	-2.73376500
H	1.69468500	0.60229300	-2.58025900
C	0.44217300	3.95174000	-1.15467100
C	1.34925300	3.94405400	-2.21680800
H	2.50160700	2.71154400	-3.55979500
H	0.10911100	4.90352800	-0.76207300
H	1.69812900	4.88754200	-2.62618100
O	-0.95040300	2.70458300	0.40503400
C	-1.47423400	3.91046000	0.99884100
H	-1.65398300	4.64227500	0.20221000
C	-0.49324900	4.47453700	2.02603100
H	-0.36876700	3.77233100	2.85789500
H	-0.87203700	5.41930200	2.42981800
H	0.48963800	4.66245300	1.58623900
C	-2.81984300	3.54158500	1.62580700
H	-3.16447000	4.41210500	2.19729200
H	-2.65520800	2.73792500	2.35595800
C	-3.89104600	3.12753800	0.61333100
H	-4.82990400	2.88231500	1.12087600
H	-3.57768500	2.25292500	0.03657100
H	-4.09795400	3.94013800	-0.09318800
N	-0.14997100	0.13156600	1.07730300
H	-0.59697900	1.00504700	1.35824600
H	-0.67302100	-0.66191400	1.44945500
H	0.80836000	0.10787500	1.42760600
C	-1.66500900	-0.25966700	-1.04105200
C	-2.49956100	-1.23916200	-0.44711100
C	-2.21223000	0.41441600	-2.14227400
C	-3.79112000	-1.49916500	-0.92066100
C	-3.49247000	0.16040400	-2.64459100
H	-1.60523600	1.16880400	-2.63355600
C	-4.28402300	-0.80006200	-2.02518800
H	-4.42363500	-2.23817000	-0.44629200
H	-3.86203300	0.71085300	-3.50445200
H	-5.28579300	-1.01296600	-2.38657100
C	0.89088500	-1.18951000	-0.94328700
C	2.17402600	-1.36158200	-0.36713000
C	0.53832700	-2.12616700	-1.92567800
C	3.02567500	-2.40408500	-0.75138200
C	1.37398400	-3.17146300	-2.33147800
H	-0.43492800	-2.03071200	-2.39744700
C	2.62280600	-3.30930800	-1.73585200
H	4.00390300	-2.52232000	-0.30339900
H	1.04916200	-3.86423900	-3.10190900
H	3.29385600	-4.11134300	-2.02898100
O	2.52469800	-0.44987900	0.61200200
O	-1.95594000	-1.92726400	0.62079700
C	-2.63302800	-3.05012400	1.22268800
H	-3.15504000	-3.60459100	0.43378700
C	3.85576700	-0.40663200	1.16760400
H	4.18828400	-1.43143300	1.37488300
C	-3.62753600	-2.56721800	2.27753800
H	-4.19819100	-3.40729200	2.68488700
H	-4.33850200	-1.85113700	1.85760000
H	-3.09738300	-2.08002400	3.10377600
C	-1.52592100	-3.94490500	1.78760700
H	-0.98726300	-3.39031700	2.56730200
H	-0.80872200	-4.13322400	0.98132600
C	3.72260300	0.33553500	2.49265000
H	4.69089900	0.39804400	2.99658400
H	3.02916000	-0.18625800	3.15953200
H	3.35437700	1.35400100	2.32792500
C	4.82204300	0.26815800	0.18539400
H	4.53815300	1.32341000	0.09189700
H	4.68373200	-0.18006900	-0.80378200
C	-2.03128700	-5.27824200	2.34919600
H	-2.67841300	-5.14061800	3.22140100
H	-1.18857500	-5.90352700	2.66043600
H	-2.59776300	-5.83768100	1.59533700
C	6.29584300	0.14787800	0.59060500
H	6.50668200	0.65519300	1.53770100
H	6.94028200	0.59632600	-0.17219300
H	6.59373600	-0.90186100	0.69999800

1b-c4

B	-0.09111800	0.02674300	-0.53499300
C	0.39429400	1.47818400	-1.13397000
C	1.27426300	1.54366700	-2.22395400
C	-0.06528800	2.72594700	-0.64362400
C	1.69028600	2.74329100	-2.80979200
H	1.64959100	0.61150400	-2.63503300
C	0.34447800	3.94031000	-1.20689100
C	1.22366400	3.94636700	-2.29226300
H	2.36983900	2.73202000	-3.65656900
H	-0.01531600	4.88583800	-0.82220900
H	1.53054000	4.89442600	-2.72407400
O	-0.93054500	2.67098700	0.43395700
C	-1.57254500	3.85444800	0.95319700
H	-0.83471000	4.66483400	1.00429100
C	-2.00065700	3.49859800	2.37253400
H	-2.71098500	2.66467100	2.36209000
H	-2.48244100	4.35364600	2.85439800
H	-1.13389500	3.21532300	2.97764200
C	-2.74281100	4.26741000	0.05081700
H	-3.51832700	3.49472200	0.11690800
H	-2.39821400	4.27435800	-0.98824500
C	-3.33036700	5.64113100	0.39513400
H	-4.12419300	5.90567000	-0.31063400
H	-2.56568800	6.42545200	0.34214900
H	-3.76374100	5.66477000	1.40032800
N	-0.01996200	0.13400900	1.09111400
H	-0.46097800	1.00430200	1.39050600
H	-0.51154200	-0.66095600	1.50103300
H	0.95765300	0.12320400	1.38423500
C	-1.65327300	-0.30442200	-0.92541400
C	-2.43348800	-1.29311500	-0.27555500
C	-2.28480200	0.36504600	-1.98363700
C	-3.74983400	-1.57158600	-0.66220100
C	-3.59520400	0.09927900	-2.39347700
H	-1.72323100	1.12821500	-2.51376300
C	-4.32947600	-0.87296900	-1.72370300
H	-4.33286700	-2.33093000	-0.15736000
H	-4.03031300	0.64651300	-3.22427400
H	-5.34975000	-1.10104000	-2.01781400
C	0.92109100	-1.18928800	-0.98034700
C	2.23041000	-1.35853200	-0.46427900
C	0.53621900	-2.10912900	-1.96661900
C	3.07019100	-2.39089400	-0.89968400
C	1.36323800	-3.13681100	-2.43041300
H	-0.45710300	-2.01353600	-2.39449300
C	2.63556600	-3.27710000	-1.88797400
H	4.06183600	-2.51888200	-0.48591100
H	1.01149000	-3.81733500	-3.19992900
H	3.29813600	-4.07081300	-2.22038900
O	2.63373100	-0.43422600	0.48212300
O	-1.82113300	-1.95413300	0.77350200
C	-2.45091400	-3.06985600	1.43763100
H	-3.50805000	-2.83061300	1.60773300
C	3.93259000	-0.50771200	1.10599000

H	4.67206500	-0.77757000	0.34259400
C	-1.75742900	-3.18701600	2.79031800
H	-2.18604100	-4.00816500	3.37126400
H	-1.87960700	-2.26599000	3.36874900
H	-0.68747800	-3.38142600	2.65824600
C	-2.33266600	-4.33785900	0.58162900
H	-1.27608400	-4.62880600	0.53933500
H	-2.63026200	-4.09649300	-0.44384400
C	3.93167400	-1.54154100	2.23153100
H	4.93495200	-1.63208100	2.66083200
H	3.62454100	-2.52792200	1.87438900
H	3.24492300	-1.23546100	3.02861200
C	4.25579000	0.89913500	1.61198900
H	5.17151500	0.82943100	2.21177700
H	3.45894100	1.21374100	2.29911300
C	-3.18565100	-5.50560900	1.09121200
H	-2.87557500	-5.84038700	2.08653300
H	-3.10285100	-6.36333900	0.41615400
H	-4.24549200	-5.22911200	1.14656400
C	4.43980100	1.93716000	0.50181000
H	5.26693700	1.65874500	-0.16203400
H	4.67066700	2.92054500	0.92470600
H	3.53722900	2.03247700	-0.10806700

1b-c5

B	-0.05531800	0.04246900	-0.54618400
C	0.48213000	1.50868700	-1.05902900
C	1.41472300	1.60915200	-2.10133800
C	0.00703500	2.74011900	-0.54258800
C	1.86548100	2.82780900	-2.61894000
H	1.80068300	0.69069200	-2.53307000
C	0.45623100	3.97249600	-1.03184500
C	1.38348800	4.01346100	-2.07595100
H	2.58509600	2.84426000	-3.43189600
H	0.09747900	4.90494200	-0.61585100
H	1.72146500	4.97507400	-2.45085300
O	-0.93200600	2.64829500	0.46691100
C	-1.60952800	3.81450000	0.97950500
H	-1.77456200	4.51281900	0.15040700
C	-0.77186900	4.47762600	2.07199400
H	-0.67321800	3.80741200	2.93347900
H	-1.24299400	5.40490500	2.41187500
H	0.23077300	4.72296000	1.71262900
C	-2.97325300	3.31836700	1.46877200
H	-2.81595300	2.60493700	2.28840100
H	-3.43620600	2.75990000	0.64799500
C	-3.91442900	4.43862600	1.92462400
H	-4.89620100	4.02902000	2.18224800
H	-4.06321500	5.18011400	1.13077600
H	-3.53522000	4.96350600	2.80732800
N	-0.05488900	0.08310900	1.08465800
H	-0.50122500	0.94587700	1.39809100
H	-0.57480700	-0.72038100	1.43942800
H	0.90753100	0.04850100	1.42202100
C	-1.60403400	-0.23908400	-1.01941200
C	-2.44788000	-1.21382600	-0.43057100
C	-2.15377000	0.46186500	-2.10283400
C	-3.75097600	-1.44020200	-0.89033300
C	-3.44535200	0.24116500	-2.59113200
H	-1.53988700	1.21395700	-2.58913400
C	-4.24659900	-0.71369900	-1.97543000
H	-4.39114000	-2.17557100	-0.42089400
H	-3.81611500	0.81296000	-3.43646600
H	-5.25742700	-0.90176800	-2.32521000
C	0.95346100	-1.17267800	-1.00079400
C	2.24189400	-1.37702300	-0.44749300
C	0.59011800	-2.05442700	-2.02930500
C	3.08777900	-2.39845500	-0.89588300
C	1.42067700	-3.07585100	-2.50044600
H	-0.38784100	-1.93306700	-2.48497700
C	2.67465300	-3.24758900	-1.92495500
H	4.06984400	-2.54208600	-0.46401300
H	1.08747200	-3.72514400	-3.30439300
H	3.34154000	-4.03321900	-2.26772700
O	2.60567300	-0.51919200	0.57482200
O	-1.90276400	-1.93736200	0.61452100
C	-2.65719700	-2.95463200	1.30551300
H	-3.24073000	-3.51803500	0.56765000
C	3.94883900	-0.49300500	1.10222300
H	4.29280700	-1.52405600	1.25130200
C	-3.58351300	-2.32480000	2.34521400
H	-4.19624400	-3.09743600	2.82131900
H	-4.25403800	-1.58772000	1.89588200
H	-2.99681700	-1.82547200	3.12437000
C	-1.63744300	-3.90292700	1.93837200
H	-2.19091600	-4.59606200	2.58366900
H	-0.98378900	-3.31927400	2.60028400
C	3.84056900	0.18158700	2.46503000
H	4.81968500	0.22691700	2.94951700
H	3.16618200	-0.37822000	3.12044800
H	3.46170000	1.20402900	2.35933600
C	4.88787900	0.23712700	0.13323200
H	4.59315200	1.29288100	0.09685700
H	4.73192700	-0.16462800	-0.87316000
C	-0.80126700	-4.69071600	0.92634400
H	-1.44052300	-5.31633700	0.29210900
H	-0.09401000	-5.35146800	1.43857400
H	-0.23163800	-4.02305200	0.27405100
C	6.37125400	0.11151500	0.50011800
H	6.59851300	0.57354900	1.46638500
H	6.99459300	0.60348500	-0.25333700
H	6.68094700	-0.93928400	0.55072700

1b-c6

B	-0.02451600	0.13959200	-0.08869500
C	0.18592700	1.66866000	-0.65460800
C	-0.55414500	2.13173900	-1.75243400
C	1.13475200	2.58101900	-0.12905200
C	-0.39137200	3.40233900	-2.31336800
H	-1.28978700	1.46453600	-2.19118600
C	1.30924100	3.86144700	-0.66721900
C	0.54412600	4.27048700	-1.76178500
H	-0.98921600	3.70380000	-3.16817100
H	2.03802500	4.54725000	-0.25496600
H	0.69230000	5.26428000	-2.17396300
O	1.86505700	2.13738000	0.95831900
C	2.95804600	2.90578800	1.50387000
H	2.65292700	3.95739400	1.57214000
C	4.19667200	2.76909500	0.62119700
H	4.54266700	1.73016000	0.61387600
H	5.00631900	3.40381000	0.99370700
H	3.98488100	3.06645000	-0.40882500
C	3.15871000	2.37613000	2.92665600
H	3.44387500	1.31779900	2.87136100
H	2.19011300	2.42056800	3.43946700
C	4.19631200	3.15715100	3.74023100
H	4.23201700	2.78506100	4.76893800
H	3.94841600	4.22435700	3.78198600
H	5.20288100	3.06220900	3.32128600
N	-0.06373700	0.23354100	1.53845900
H	-0.94541500	0.65936900	1.82555200
H	0.00903400	-0.70614400	1.93014900
H	0.71694600	0.80558300	1.86220900
C	-1.46378800	-0.49592400	-0.56485500
C	-2.71938600	-0.06573600	-0.06898400
C	-1.51282100	-1.48790000	-1.55569600
C	-3.92461900	-0.60144900	-0.53784800
C	-2.70370300	-2.03381500	-2.04401500
H	-0.57411900	-1.84548100	-1.96794400
C	-3.91490900	-1.58643600	-1.52757700
H	-4.87592700	-0.26092600	-0.14959600
H	-2.67978900	-2.79593900	-2.81709100
H	-4.85658100	-1.99163500	-1.88610500
C	1.23720100	-0.83738700	-0.48115200
C	1.49269800	-2.08046000	0.15019500
C	2.10648100	-0.50024900	-1.52846600
C	2.55688300	-2.90369500	-0.23720200
C	3.16523200	-1.31355800	-1.94517000
H	1.94353800	0.44046200	-2.04558000
C	3.39014000	-2.51924600	-1.29054400

H	2.75169400	-3.84221100	0.26524100
H	3.80384600	-1.00262300	-2.76641900
H	4.20995900	-3.16786800	-1.58494700
O	0.62017800	-2.43311300	1.16245300
O	-2.68637300	0.89786500	0.92440600
C	-3.87290800	1.61970500	1.31782400
H	-4.69505700	0.90660800	1.45468800
C	0.69941200	-3.71608500	1.81801500
H	0.98347500	-4.47036800	1.07461000
C	-4.24286700	2.65579900	0.25888400
H	-5.16724900	3.16925300	0.54353100
H	-4.39941500	2.19123000	-0.71810500
H	-3.44591500	3.40006200	0.16137300
C	-3.56588900	2.25682300	2.67396200
H	-4.38835800	2.94310500	2.90773100
H	-2.66519400	2.87571800	2.57148900
C	-0.71213100	-4.02088000	2.30713300
H	-0.74822300	-5.00253900	2.78776300
H	-1.41475100	-4.02211600	1.46964300
H	-1.03983300	-3.27064100	3.03547300
C	1.74076300	-3.67354400	2.94502700
H	1.37658500	-2.99276100	3.72559000
H	2.66366100	-3.23298600	2.55326300
C	-3.40130800	1.25383900	3.82002900
H	-4.31412200	0.66319000	3.95982900
H	-3.19156800	1.77072800	4.76214300
H	-2.58083900	0.55324100	3.63464000
C	2.05482500	-5.04688100	3.55034900
H	1.18060500	-5.49266200	4.03544900
H	2.84281900	-4.96251700	4.30549200
H	2.40486100	-5.74762300	2.78306900

1b-c7

B	-0.02559600	0.00715100	-0.09163000
C	0.18972100	1.54288800	-0.63537000
C	-0.60464300	2.04621800	-1.67648000
C	1.19250000	2.42040200	-0.15382100
C	-0.44260600	3.32197000	-2.22485200
H	-1.38321600	1.40636500	-2.08050700
C	1.36827700	3.70534900	-0.68096100
C	0.54941100	4.15481700	-1.71879700
H	-1.08380900	3.65464200	-3.03554100
H	2.13905000	4.36394700	-0.30185200
H	0.69892800	5.15182900	-2.12263700
O	1.97507600	1.94470300	0.88418200
C	3.19465700	2.61056700	1.27596700
H	2.99922100	3.68624000	1.36647600
C	4.29698000	2.35906400	0.24935400
H	4.53703400	1.29211200	0.20218100
H	5.20199000	2.90767600	0.53072100
H	3.99777500	2.68753100	-0.74925300
C	3.55858600	2.07503100	2.66160200
H	4.56397400	2.44127800	2.90072500
H	3.63278100	0.98149300	2.60313500
C	2.59056100	2.48947200	3.77398800
H	2.90896800	2.08120700	4.73871100
H	1.57287200	2.13512400	3.58198000
H	2.54722300	3.58045100	3.87167800
N	0.03986100	0.06225200	1.53736200
H	-0.80722000	0.50725000	1.89266700
H	0.10912600	-0.88813700	1.90290100
H	0.85730300	0.60297900	1.82071600
C	-1.50813700	-0.58119200	-0.48702400
C	-2.71438400	-0.11459700	0.09115700
C	-1.65196400	-1.56751800	-1.47363100
C	-3.96384100	-0.61470400	-0.29326900
C	-2.88904100	-2.07648500	-1.88116900
H	-0.75406900	-1.95204400	-1.94795100
C	-4.04898000	-1.59718300	-1.28218100
H	-4.87613200	-0.24832400	0.15985300
H	-2.94002000	-2.83626100	-2.65523400
H	-5.02393500	-1.97479000	-1.57607600
C	1.18156700	-0.99328000	-0.58260600
C	1.42918700	-2.26290700	-0.00315700
C	2.00366300	-0.65126900	-1.66572000
C	2.44056200	-3.10856000	-0.47453800
C	3.00909000	-1.48542000	-2.16514900
H	1.84454500	0.30980500	-2.14523000
C	3.22715300	-2.71850900	-1.56120100
H	2.62975300	-4.06852200	-0.01206000
H	3.61189500	-1.16984500	-3.01132300
H	4.00600000	-3.38437300	-1.92107300
O	0.60435400	-2.61647400	1.04778700
O	-2.58144400	0.84691100	1.07652400
C	-3.71953800	1.57092800	1.58943300
H	-4.52565200	0.85929300	1.80765100
C	0.66029800	-3.92877000	1.64507900
H	0.86659400	-4.66134100	0.85559600
C	-4.18968200	2.60944000	0.57356300
H	-5.08617400	3.12218300	0.93483500
H	-4.43376300	2.14452000	-0.38488000
H	-3.40475900	3.35388900	0.40440000
C	-3.25099600	2.17796100	2.91487600
H	-2.43208000	2.87934600	2.71067900
H	-2.83360500	1.36718200	3.52447500
C	-0.73426600	-4.19115600	2.20269500
H	-0.78957800	-5.18998000	2.64455800
H	-1.48200100	-4.12460600	1.40808700
H	-0.98478400	-3.45902400	2.97858100
C	1.76392200	-3.98264100	2.71070700
H	1.47575900	-3.32282300	3.53950700
H	2.68297200	-3.56546000	2.28584700
C	-4.36084500	2.88177400	3.70307700
H	-4.74367300	3.76168600	3.17681200
H	-3.98376200	3.21788600	4.67406900
H	-5.20527000	2.20791500	3.89030800
C	2.04745500	-5.39455700	3.23746500
H	1.18104000	-5.82432100	3.75025200
H	2.87750900	-5.37862100	3.95093200
H	2.32426200	-6.07390000	2.42247000

1b-c8

B	-0.02948300	0.15697300	-0.29467200
C	0.46817700	1.62258200	-0.84669400
C	-0.20395500	2.25910700	-1.90017900
C	1.60619700	2.30341400	-0.34774100
C	0.19732400	3.48345800	-2.44381700
H	-1.07970400	1.77127100	-2.31727700
C	2.02241200	3.53397400	-0.86896100
C	1.31547800	4.12281300	-1.91976200
H	-0.35887800	3.92644400	-3.26444600
H	2.89398900	4.04174800	-0.47611700
H	1.65034700	5.07531400	-2.31989500
O	2.26762200	1.68969400	0.70037400
C	3.55242000	2.15516300	1.16402300
H	3.51934900	3.24696900	1.26721200
C	4.65058300	1.74883300	0.18370000
H	4.72906500	0.65777500	0.13459000
H	5.61648500	2.15471500	0.49910900
H	4.44283100	2.12328900	-0.82174900
C	3.72360800	1.55201800	2.56104100
H	3.74063200	0.45831000	2.47267300
H	2.83435000	1.81199900	3.14815400
C	4.97550400	2.03891700	3.29860900
H	4.99630700	1.64027600	4.31774500
H	4.99539000	3.13286400	3.36985900
H	5.89610800	1.71794500	2.80137500
N	-0.00841300	0.23242500	1.33514300
H	-0.78037600	0.81883800	1.65411300
H	-0.11057300	-0.70891700	1.71592600
H	0.87860900	0.63349000	1.64164900
C	-1.57762000	-0.17376600	-0.73765300
C	-2.70760400	0.49620800	-0.20598900
C	-1.85184700	-1.13160700	-1.72479300
C	-4.00922100	0.21013600	-0.63430300
C	-3.14186600	-1.42859700	-2.17557800
H	-1.01592900	-1.66749600	-2.16395400
C	-4.22419000	-0.75396900	-1.62177300
H	-4.86225100	0.73055700	-0.21825600
H	-3.29295300	-2.17668500	-2.94794400

H	-5.23795100	-0.96391300	-1.94997600
C	1.00256900	-1.04523400	-0.73188100
C	1.01740000	-2.32627600	-0.12642800
C	1.90665400	-0.86557200	-1.78893800
C	1.88320600	-3.33936600	-0.55452100
C	2.77547000	-1.86418700	-2.24007900
H	1.92817200	0.09979300	-2.28536700
C	2.76228900	-3.10589300	-1.61444600
H	1.88167100	-4.31323400	-0.08206000
H	3.44983300	-1.66955800	-3.06855700
H	3.42589900	-3.90094500	-1.94157200
O	0.13958000	-2.50820800	0.92654400
O	-2.44818600	1.43500500	0.77566600
C	-3.47992200	2.30509700	1.28607700
H	-4.38214900	1.71123600	1.47872600
C	-0.08388500	-3.80766800	1.51305100
H	0.88493800	-4.29821300	1.66961700
C	-3.78108600	3.41724300	0.28386300
H	-4.60849700	4.03981300	0.63741100
H	-4.06229600	3.00832100	-0.68974900
H	-2.89936700	4.05216800	0.14802800
C	-2.95241300	2.81731400	2.62935400
H	-2.03668000	3.39506900	2.45036600
H	-2.66839500	1.94537800	3.23119000
C	-0.96696700	-4.65733300	0.60180100
H	-1.08484000	-5.66501600	1.01149900
H	-0.53226800	-4.75098800	-0.39650000
H	-1.95792700	-4.20188200	0.50364400
C	-0.70441300	-3.53348700	2.88594000
H	-1.66514600	-3.02285600	2.74224300
H	-0.04627200	-2.83458800	3.41654500
C	-3.96222900	3.66331400	3.41225100
H	-4.20434200	4.59688100	2.89499400
H	-3.55752300	3.92714100	4.39438800
H	-4.89841200	3.11641500	3.57535000
C	-0.90050700	-4.78973100	3.74142300
H	-1.62863000	-5.47753700	3.30040200
H	-1.26541000	-4.51909200	4.73720800
H	0.04204200	-5.33482300	3.87038600

1b-c9

B	-0.03056700	0.05494000	-0.13014400
C	0.20998300	1.58728200	-0.67161100
C	-0.57181400	2.11361400	-1.70976200
C	1.23260900	2.43820600	-0.18329600
C	-0.38135700	3.39046900	-2.24787300
H	-1.36269800	1.49305200	-2.12009700
C	1.43241700	3.72631400	-0.69432700
C	0.62521900	4.19894000	-1.73196700
H	-1.01532300	3.74418400	-3.05531900
H	2.20585300	4.37260800	-0.30009300
H	0.79151900	5.19837000	-2.12309400
O	2.02386400	1.91370600	0.82105100
C	3.18594300	2.61433700	1.31163800
H	3.65383400	3.14176900	0.47183900
C	4.14448700	1.53667700	1.80617900
H	3.69916800	0.97225700	2.63311200
H	5.07631500	1.98553000	2.16153500
H	4.38432100	0.83932300	0.99914200
C	2.78124000	3.61819000	2.40013900
H	2.42066800	3.05586400	3.27141700
H	1.93237100	4.20680200	2.03645400
C	3.91097000	4.56700500	2.81801700
H	3.55067500	5.29242000	3.55438100
H	4.29395000	5.12981900	1.95844200
H	4.75291400	4.03143900	3.26820800
N	0.01426100	0.11561300	1.49969000
H	-0.83159000	0.57195900	1.84322300
H	0.06691900	-0.83457600	1.86755700
H	0.83648000	0.64489200	1.79203600
C	-1.51330200	-0.51361000	-0.54986000
C	-2.72359000	-0.04858600	0.02251400
C	-1.65245100	-1.46632300	-1.56926800
C	-3.97187900	-0.53044900	-0.38977500
C	-2.88847300	-1.94823800	-2.01197900
H	-0.75120900	-1.84418000	-2.04257700
C	-4.05171600	-1.47827400	-1.41291900
H	-4.88748100	-0.18125400	0.06948900
H	-2.93542400	-2.68294900	-2.81008000
H	-5.02565700	-1.84121900	-1.72784800
C	1.17338800	-0.96016800	-0.59913000
C	1.40338500	-2.23096900	-0.01635700
C	2.02230000	-0.61950800	-1.66268700
C	2.41387400	-3.08438900	-0.47488800
C	3.03402500	-1.45700100	-2.14190800
H	1.88032200	0.34450900	-2.14169400
C	3.22882600	-2.69544200	-1.53999300
H	2.57637200	-4.05311300	-0.02008400
H	3.65683900	-1.14235700	-2.97386100
H	4.00684800	-3.36785900	-1.88932900
O	0.58831300	-2.57035100	1.04971300
O	-2.59528000	0.91700400	1.00326900
C	-3.74754600	1.55544000	1.59194800
H	-4.50951600	1.68450800	0.81410100
C	0.52353400	-3.92284100	1.55012100
H	1.54360600	-4.30900900	1.66655500
C	-3.27386600	2.93248500	2.04366500
H	-4.10220600	3.50085900	2.47592600
H	-2.87495800	3.49620500	1.19617400
H	-2.48816100	2.84176400	2.80205000
C	-4.30592100	0.69591200	2.73471700
H	-3.56992300	0.68430100	3.54932200
H	-4.39816600	-0.33728400	2.38352800
C	-0.27056300	-4.80970200	0.59370700
H	-0.27789300	-5.84192300	0.95919700
H	0.16433400	-4.80765600	-0.40912800
H	-1.30469900	-4.45821100	0.52025700
C	-0.11632400	-3.84429500	2.93703200
H	-0.32790500	-4.87068800	3.25936100
H	-1.08761700	-3.34177200	2.84347500
C	-5.66488800	1.17275200	3.26103100
H	-5.60839300	2.17445200	3.69901100
H	-6.03568000	0.49497800	4.03654100
H	-6.41275700	1.19998400	2.45959800
C	0.74885700	-3.14960800	3.99300900
H	1.70208100	-3.67385800	4.12782400
H	0.23956500	-3.13011900	4.96202600
H	0.97787500	-2.11555900	3.71622500

1b-c10

B	-0.09368000	0.07607200	-0.47750400
C	0.70512000	1.39230900	-1.04963800
C	1.59756500	1.27931200	-2.12524700
C	0.50990800	2.70447800	-0.55161000
C	2.26576400	2.36966600	-2.69153200
H	1.77280700	0.29250800	-2.54302900
C	1.17710100	3.81000900	-1.09271000
C	2.05294300	3.63988500	-2.16765100
H	2.94308000	2.22258000	-3.52736800
H	1.02867700	4.80485400	-0.69340800
H	2.56259200	4.50505900	-2.58146600
O	-0.38542400	2.82477100	0.49375600
C	-0.77898600	4.11356400	1.00889500
H	-0.84192200	4.81801300	0.17088200
C	0.23473400	4.60630800	2.04077300
H	0.24265200	3.94088500	2.91149800
H	-0.01990600	5.61416700	2.38243400
H	1.24510100	4.63820000	1.62526500
C	-2.18665300	3.91815900	1.57963300
H	-2.13298700	3.21375000	2.42020700
H	-2.79437200	3.43900200	0.80456500
C	-2.85904400	5.21927000	2.03087400
H	-3.88635300	5.02417400	2.35427600
H	-2.90236700	5.94822700	1.21303800
H	-2.33352000	5.68698000	2.86967800
N	-0.01737900	0.14478600	1.15250600
H	-0.28292900	1.08025600	1.46209000
H	-0.65811500	-0.54485700	1.54688000
H	0.93627700	-0.06030500	1.45263600
C	-1.68652400	0.10033400	-0.87811100

C	-2.65893000	-0.72236000	-0.25714800
C	-2.15758600	0.91708900	-1.91576500
C	-4.00308400	-0.70691300	-0.64826100
C	-3.49196800	0.94013000	-2.33419600
H	-1.44583700	1.56010100	-2.42466600
C	-4.41667900	0.12380100	-1.69253400
H	-4.73780300	-1.32984000	-0.15486700
H	-3.79917500	1.59017900	-3.14788200
H	-5.46111300	0.12522900	-1.99031200
C	0.63232400	-1.32675500	-0.92819300
C	1.88307800	-1.75874300	-0.42119500
C	0.05616500	-2.15315800	-1.90345200
C	2.49069100	-2.94280100	-0.85606900
C	0.65056300	-3.33179600	-2.36550000
H	-0.89955300	-1.85708400	-2.32540100
C	1.87315200	-3.72655200	-1.83372900
H	3.43842300	-3.26916900	-0.44794400
H	0.15965500	-3.93030100	-3.12696200
H	2.35542300	-4.64105100	-2.16631400
O	2.46790400	-0.93563100	0.52229300
O	-2.19527300	-1.54402600	0.75208000
C	-3.04106400	-2.53629100	1.36944900
H	-3.71108800	-2.94656200	0.60453200
C	3.80230100	-1.17786400	1.01462100
H	4.41010400	-1.57213800	0.19159400
C	-3.85173100	-1.91057700	2.50351600
H	-4.54620200	-2.64002100	2.93123700
H	-4.43632000	-1.05642300	2.15228300
H	-3.18378500	-1.56460800	3.30053200
C	-2.09937000	-3.65208600	1.83165500
H	-1.41715600	-3.24714100	2.59074000
H	-1.48198400	-3.94005700	0.97385900
C	3.76794500	-2.17471200	2.17222400
H	4.78201100	-2.41873300	2.50301500
H	3.27641900	-3.10681200	1.88199000
H	3.22273900	-1.74979000	3.02266900
C	4.35537100	0.19607900	1.40411100
H	3.74518200	0.60499000	2.22022400
H	4.22095200	0.86199300	0.54481600
C	-2.82280600	-4.88535900	2.38321700
H	-3.38027300	-4.66256600	3.29868300
H	-2.10244100	-5.67446400	2.62070400
H	-3.52946400	-5.29199100	1.65013200
C	5.83169100	0.17706800	1.81493000
H	5.99920800	-0.40249000	2.72846600
H	6.18748000	1.19518100	2.00170200
H	6.45844800	-0.25275100	1.02463600

Conformers of 1c

1c-c1

C	0.10456300	-4.53960600	1.50404600
C	0.29235500	-4.75303500	2.87141800
C	0.32297100	-3.66860700	3.74072000
C	0.17045400	-2.37736700	3.22614200
C	-0.01888100	-2.10382000	1.86315100
C	-0.04861000	-3.23806600	1.01540700
H	0.05777300	-5.39383400	0.84191800
H	0.40388100	-5.76750000	3.24225500
H	0.46057300	-3.81874600	4.80723200
H	0.19706100	-1.54062000	3.91730000
B	-0.25571400	-0.55972200	1.34931200
C	0.30203600	0.53731300	2.43977000
C	-0.59188400	1.27021000	3.23469500
C	1.67498300	0.77561800	2.69294200
C	-0.18471900	2.18096700	4.21470400
H	-1.65588100	1.11882100	3.08128500
C	2.10868100	1.68421900	3.66369500
C	1.17364600	2.38912900	4.42491000
H	-0.92345800	2.71554900	4.80412600
H	3.16255700	1.83698200	3.85429400
H	1.51947000	3.08755800	5.18102200
C	-1.82812700	-0.28413700	0.95412800
C	-2.84329100	-1.11359600	1.45399800
C	-2.26873100	0.80094300	0.15748700
C	-4.20284400	-0.90963700	1.19817100
H	-2.55640900	-1.95738300	2.07382600
C	-3.62166900	1.02418800	-0.11787700
C	-4.59008000	0.16384000	0.40420600
H	-4.94441700	-1.58263200	1.61777100
H	-3.93809700	1.86992400	-0.71361200
H	-5.63851600	0.34893100	0.19058200
O	-0.24260100	-2.98504500	-0.33413000
O	2.57040800	0.05097900	1.92118300
O	-1.27760700	1.63645100	-0.33489400
N	0.61956400	-0.36657400	-0.01129300
H	0.33255500	0.49552700	-0.47539200
H	1.60942100	-0.31459700	0.23073400
H	0.45829000	-1.16132800	-0.63046600
C	3.98486600	0.27069400	2.02765300
H	4.17566800	1.35067100	2.05094100
C	-0.20671500	-4.04790300	-1.29826400
H	0.64300500	-4.70375400	-1.07264300
C	-1.59621600	2.71935400	-1.22154400
H	-2.30307100	2.36125000	-1.98014000
C	0.04128900	-3.37667900	-2.65067000
H	0.07794800	-4.13129100	-3.44111100
H	0.99435500	-2.83787700	-2.64282800
H	-0.76642900	-2.67552800	-2.88322800
C	4.59368200	-0.30576700	0.74782100
H	5.67914000	-0.17478300	0.75866600
H	4.19236700	0.20605300	-0.13288100
H	4.37788000	-1.37604600	0.67071400
C	-0.28364700	3.09163200	-1.91408700
H	0.46095400	3.40765600	-1.17670900
H	-0.45021300	3.91901900	-2.60925100
H	0.10861900	2.23948900	-2.47854000
C	-2.20001000	3.91247800	-0.49660900
C	-3.07905400	4.76234200	-1.17682100
C	-1.84783600	4.21555300	0.82235400
C	-3.59441700	5.90042900	-0.55484900
H	-3.36717100	4.53189000	-2.19955800
C	-2.36624200	5.35041700	1.44792800
H	-1.17964700	3.55463100	1.36408900
C	-3.23826300	6.19790300	0.76188100
H	-4.27735100	6.54841600	-1.09540000
H	-2.08924400	5.57043200	2.47428300
H	-3.64150100	7.07904300	1.25085000
C	4.58600000	-0.37158300	3.26846800
C	5.73013500	0.18540800	3.85029600
C	4.05355800	-1.54639900	3.80869900
C	6.33826100	-0.42210400	4.94976400
H	6.14809300	1.10346800	3.44443600
C	4.65719800	-2.15259100	4.91148600
H	3.15764900	-1.97788300	3.37513600
C	5.80209900	-1.59520200	5.48405600
H	7.22393700	0.02401900	5.39138500
H	4.22956900	-3.06089800	5.32457000
H	6.26892900	-2.06734000	6.34269000
C	-1.48618500	-4.87028900	-1.31047500
C	-1.44056600	-6.21486000	-1.69486000
C	-2.72283500	-4.29426000	-1.00294800
C	-2.60975200	-6.97229500	-1.77844000
H	-0.48379600	-6.67613000	-1.92716700
C	-3.89268500	-5.05143000	-1.08091700
H	-2.76744400	-3.25726600	-0.68762400
C	-3.84132500	-6.39096400	-1.47132700
H	-2.55718100	-8.01501300	-2.07585400
H	-4.84524800	-4.59322900	-0.83350400
H	-4.75213800	-6.97841700	-1.52981600

1c-c2

C	0.57795000	-4.34939900	1.56051200
C	-0.66582400	-4.77194700	2.03459600
C	-1.69706100	-3.85022200	2.18171500
C	-1.47060200	-2.51187100	1.84580800
C	-0.24044100	-2.03180200	1.37200100
C	0.78214900	-3.00168500	1.24729000
H	1.36901300	-5.07607100	1.42736600
H	-0.81667700	-5.81942300	2.27796700

H	-2.66882300	-4.16356400	2.55127900
H	-2.28493300	-1.80405400	1.96722000
B	-0.01820700	-0.43378800	1.06593000
C	0.93976100	0.27989400	2.19238500
C	1.12803800	-0.30574300	3.45330700
C	1.56533000	1.53524600	2.00687400
C	1.87457700	0.28947800	4.47495500
H	0.66002300	-1.26604600	3.64757700
C	2.32940500	2.14618800	3.00635600
C	2.47900800	1.52112400	4.24620900
H	1.98032000	-0.20742800	5.43463600
H	2.82058300	3.09412900	2.82855400
H	3.07215000	2.00195100	5.01832500
C	-1.44431600	0.36016200	0.88583000
C	-1.91388100	1.23557100	1.87643100
C	-2.31131700	0.16939100	-0.21585600
C	-3.15114500	1.88354400	1.80770000
H	-1.28782500	1.40852600	2.74647700
C	-3.54780800	0.81507400	-0.31643300
C	-3.96941200	1.67121600	0.70320400
H	-3.46623100	2.54517800	2.60897400
H	-4.18031500	0.67161100	-1.18300200
H	-4.93143800	2.16790900	0.61960100
O	2.00668900	-2.54274700	0.78784900
O	1.38296300	2.13020500	0.76834900
O	-1.86276000	-0.69532900	-1.20155900
N	0.76597500	-0.33188800	-0.36301500
H	1.06977400	0.63050500	-0.51336500
H	1.57840700	-0.94892500	-0.34594700
H	0.13597800	-0.61485200	-1.11419000
C	1.62971200	3.53234900	0.58755300
H	1.29721400	4.06005800	1.48946400
C	3.20012700	-3.31229600	1.00071800
H	3.12736700	-3.79633500	1.98181900
C	-2.78086000	-1.26696500	-2.14497700
H	-3.72080900	-1.48359600	-1.62342100
C	4.35165400	-2.30556200	1.02929300
H	5.29716700	-2.82349800	1.21345100
H	4.19012100	-1.56829400	1.82054800
H	4.42976800	-1.78613600	0.06857500
C	0.75450500	3.96419800	-0.59062000
H	0.86763100	5.03743900	-0.76789200
H	-0.29643100	3.74729600	-0.38043900
H	1.05274000	3.43552900	-1.50199800
C	-2.15037900	-2.58481000	-2.59905000
H	-1.19596500	-2.39827700	-3.10241400
H	-2.81307100	-3.09365700	-3.30470400
H	-1.97801600	-3.23739800	-1.73878500
C	-3.06584600	-0.34932700	-3.32503200
C	-4.28881300	-0.44938800	-3.99807200
C	-2.11069400	0.55757300	-3.79430900
C	-4.55211000	0.33651100	-5.12081100
H	-5.04419000	-1.14393300	-3.63906000
C	-2.37213400	1.34856000	-4.91418300
H	-1.16393500	0.65397600	-3.27350400
C	-3.59272200	1.23940100	-5.58285400
H	-5.50731800	0.24803800	-5.62888000
H	-1.62254900	2.05244600	-5.26249000
H	-3.79654300	1.85611700	-6.45233500
C	3.09718900	3.84897700	0.33739200
C	3.60442600	5.10203700	0.69972500
C	3.94480500	2.93794200	-0.30031700
C	4.92999800	5.44203500	0.42631100
H	2.95962000	5.81688700	1.20470400
C	5.27249600	3.27263900	-0.57123500
H	3.56852500	1.95796000	-0.57381500
C	5.76938100	4.52653300	-0.21113900
H	5.30736900	6.41746000	0.71703300
H	5.91960500	2.55176700	-1.06137000
H	6.80240000	4.78559300	-0.42003600
C	3.41514900	-4.37720900	-0.06491300
C	4.10021200	-5.55326400	0.26067000
C	2.99462100	-4.18636700	-1.38490900
C	4.36504300	-6.51824700	-0.71219900
H	4.42567300	-5.71935300	1.28451800
C	3.25336400	-5.15112800	-2.35959700
H	2.45265700	-3.28449100	-1.64911600
C	3.94123400	-6.31973000	-2.02738600
H	4.89511700	-7.42588600	-0.44090600
H	2.91502200	-4.99055900	-3.37861700
H	4.14045800	-7.07073600	-2.78513600

1c-c3

C	3.78196700	-1.39916200	1.58102500
C	3.60640500	-2.06853300	2.79409800
C	2.41482100	-1.92169300	3.49631300
C	1.40672200	-1.10840900	2.96925400
C	1.53124700	-0.40919300	1.75941600
C	2.76403800	-0.57782600	1.08550300
H	4.70339000	-1.53520700	1.02962300
H	4.40185200	-2.70266400	3.17402700
H	2.26507300	-2.43322400	4.44237100
H	0.48221800	-1.00342900	3.52898600
B	0.33634700	0.58723500	1.23079300
C	0.75850400	2.16903100	1.37102600
C	1.80138400	2.56062500	2.22304300
C	0.09881500	3.22185700	0.69063900
C	2.19337200	3.89083600	2.40287400
H	2.33196000	1.78805200	2.77108000
C	0.48002800	4.55972200	0.84392000
C	1.53045700	4.89229200	1.70244400
H	3.00617600	4.13488600	3.08014300
H	-0.03006600	5.35375800	0.31420200
H	1.81466100	5.93403200	1.81766000
C	-1.09879100	0.25882100	1.95687000
C	-1.63660900	1.12640100	2.91940400
C	-1.83927200	-0.92409000	1.72463900
C	-2.81857300	0.86287700	3.61846300
H	-1.09977700	2.04439500	3.13892800
C	-3.03251200	-1.20493400	2.39819400
C	-3.51935500	-0.30893600	3.35236100
H	-3.18393600	1.56816800	4.35883300
H	-3.59329600	-2.10473300	2.17967700
H	-4.44511900	-0.53412700	3.87341500
O	2.90862800	0.11672600	-0.10507900
O	-0.93639900	2.84113400	-0.14502500
O	-1.32101500	-1.79608300	0.77987300
N	0.14397900	0.28710000	-0.36258800
H	-0.46017100	0.99890500	-0.77533600
H	1.05522700	0.30797900	-0.82114300
H	-0.27827100	-0.63356800	-0.48470200
C	-1.71769300	3.81819700	-0.86655000
H	-1.04366700	4.58047300	-1.27089100
C	4.21149100	0.34489200	-0.66377700
H	4.91463500	0.51603300	0.15986000
C	-1.69323200	-3.18352400	0.79115100
H	-1.82704200	-3.49452000	1.83395300
C	4.09731700	1.62643200	-1.49154700
H	5.06673600	1.87321100	-1.93383500
H	3.77328400	2.45759300	-0.85901700
H	3.37570200	1.49462800	-2.30446200
C	-2.75768300	4.46872600	0.04906200
H	-3.33740600	5.20629300	-0.51504400
H	-2.28286600	4.97468500	0.89320500
H	-3.44609000	3.71544400	0.44215200
C	-0.50873900	-3.94529100	0.19355200
H	-0.33098200	-3.62711200	-0.83898300
H	-0.71925400	-5.01859900	0.18683400
H	0.39436900	-3.76399800	0.78251900
C	-2.97695600	-3.45847400	0.02212000
C	-3.81026400	-4.50996300	0.41974000
C	-3.31858300	-2.72132800	-1.11620100
C	-4.96020600	-4.82399400	-0.30622600
H	-3.56131300	-5.08589000	1.30746700
C	-4.46955100	-3.03016300	-1.84248500
H	-2.68755200	-1.89610000	-1.42892600
C	-5.29359900	-4.08361400	-1.44163300
H	-5.59667900	-5.64113400	0.01867000
H	-4.72313500	-2.44721000	-2.72252500
H	-6.18943200	-4.32184800	-2.00610500

C	-2.34771500	3.08898100	-2.03766200
C	-2.10970200	3.52182200	-3.34557200
C	-3.18822500	1.98597500	-1.83361000
C	-2.70075700	2.87290000	-4.43242900
H	-1.45755400	4.37354700	-3.51793900
C	-3.77395600	1.33163700	-2.91679600
H	-3.38350600	1.63488700	-0.82534000
C	-3.53411500	1.77483400	-4.22019000
H	-2.50673700	3.22444400	-5.44089100
H	-4.42367100	0.47978800	-2.74234500
H	-3.99441900	1.26771100	-5.06223300
C	4.70602900	-0.82231000	-1.50538100
C	6.07851100	-1.08080400	-1.59191100
C	3.82329700	-1.61398900	-2.24691200
C	6.56231900	-2.10652400	-2.40542600
H	6.77556700	-0.47864300	-1.01461000
C	4.30278900	-2.64372600	-3.05769000
H	2.75589600	-1.43107800	-2.18181200
C	5.67408800	-2.89255200	-3.14149500
H	7.63009500	-2.29492400	-2.45789300
H	3.60430100	-3.25316800	-3.62277500
H	6.04635900	-3.69475700	-3.77060100

1c-c4

C	3.42449900	-1.14647300	-1.86159100
C	3.49746200	-0.80446000	-3.21364000
C	2.34038100	-0.44418700	-3.89673100
C	1.12078900	-0.42471600	-3.21311100
C	0.98887300	-0.76506700	-1.85851800
C	2.18869200	-1.13534200	-1.20643500
H	4.33099600	-1.40509500	-1.32929500
H	4.45959200	-0.81762300	-3.71702900
H	2.38104500	-0.17784100	-4.94864400
H	0.22633600	-0.14135300	-3.75967500
B	-0.48322800	-0.79350500	-1.13102800
C	-0.97482600	-2.32889900	-0.80939400
C	-0.39504400	-3.42443500	-1.46523500
C	-2.01569500	-2.64301000	0.09860900
C	-0.78876100	-4.74914000	-1.25066900
H	0.40182200	-3.23178000	-2.17718900
C	-2.41947400	-3.96075200	0.34074300
C	-1.80252800	-5.01490000	-0.33687600
H	-0.30640400	-5.55765000	-1.79158800
H	-3.21272800	-4.18500600	1.04220700
H	-2.12666700	-6.03355800	-0.14566700
C	-1.61277000	0.04752700	-1.97703500
C	-2.64380800	-0.61595500	-2.65773600
C	-1.60705000	1.45818500	-2.10383600
C	-3.61974300	0.04297100	-3.41192400
H	-2.68472300	-1.69901700	-2.59409000
C	-2.58060900	2.14235000	-2.84060100
C	-3.58827800	1.43054800	-3.49556900
H	-4.39230200	-0.52416300	-3.92221000
H	-2.56707900	3.22152500	-2.92297600
H	-4.33552300	1.97079700	-4.06921500
O	2.07764800	-1.48229800	0.13085500
O	-2.59557600	-1.56434900	0.74187800
O	-0.58880600	2.11638900	-1.43753600
N	-0.30971300	-0.04232800	0.30880600
H	-1.14702500	-0.18558300	0.87477300
H	0.49720700	-0.43095000	0.79731400
H	-0.16844700	0.95751300	0.16031800
C	-3.72889800	-1.73011900	1.62023200
H	-3.55388200	-2.59651200	2.26671800
C	3.07991400	-2.30175600	0.75389800
H	3.45435700	-3.01300100	0.00834400
C	-0.39546900	3.53981800	-1.58016300
H	-1.36676200	4.03951900	-1.50179500
C	2.36563200	-3.07577800	1.86353100
H	3.07259800	-3.73716900	2.37265000
H	1.55495700	-3.67731200	1.44343200
H	1.95031400	-2.38560600	2.60512700
C	-5.01761300	-1.92620100	0.81712200
H	-5.86361600	-2.05110900	1.50046700
H	-4.95668000	-2.81030400	0.17773100
H	-5.20962300	-1.05505200	0.18450400
C	0.25797500	3.87163700	-2.92456800
H	1.23818300	3.39293500	-3.00121400
H	0.39354200	4.95434000	-3.01182400
H	-0.35823100	3.53231000	-3.76073400
C	0.44975500	3.98246400	-0.40158100
C	-0.00840500	4.98880600	0.45376500
C	1.70769600	3.41276800	-0.16198700
C	0.77239700	5.42575100	1.52663800
H	-0.98329200	5.43640100	0.28104900
C	2.48629600	3.84152000	0.91242700
H	2.07846100	2.62940400	-0.81537000
C	2.02209500	4.85175200	1.75902600
H	0.40153300	6.20976100	2.17935000
H	3.45877400	3.39111100	1.08435700
H	2.63095200	5.18779100	2.59238300
C	-3.77561700	-0.49596100	2.50036000
C	-3.76237400	-0.62332200	3.89238000
C	-3.85034900	0.78651700	1.93971100
C	-3.82783800	0.50465900	4.71404100
H	-3.69956700	-1.61146400	4.33969500
C	-3.90702400	1.91480500	2.75710500
H	-3.85939700	0.90320900	0.86064800
C	-3.89869300	1.77723700	4.14766300
H	-3.81822500	0.38694100	5.79306600
H	-3.96166700	2.90193200	2.30880700
H	-3.94612300	2.65602700	4.78289200
C	4.24792400	-1.49484600	1.30083000
C	5.52790200	-2.05962200	1.32721400
C	4.06375100	-0.21721100	1.83930500
C	6.60377000	-1.36582000	1.88341500
H	5.68676000	-3.04878700	0.90518300
C	5.13778300	0.48089000	2.39299200
H	3.07868000	0.23689000	1.81695900
C	6.41121200	-0.09127300	2.41892500
H	7.59048500	-1.81824700	1.89260700
H	4.97961800	1.47272800	2.80514800
H	7.24615900	0.45316200	2.84830300

1c-c5

C	1.13906000	-4.18566500	1.32701600
C	-0.08327200	-4.72433500	1.73569600
C	-1.20135800	-3.90367900	1.83321700
C	-1.08307200	-2.54943700	1.50489900
C	0.11616500	-1.95682900	1.08397900
C	1.23414400	-2.82424800	1.01697200
H	2.00111500	-4.83732000	1.26516400
H	-0.14522900	-5.78092100	1.97882400
H	-2.15607200	-4.30659500	2.15740900
H	-1.96589100	-1.92177400	1.57884200
B	0.18906900	-0.35092400	0.74177300
C	1.03362800	0.48395200	1.87788000
C	1.29715100	-0.07194800	3.13779400
C	1.50938500	1.80364300	1.68197100
C	1.98579900	0.60349200	4.15041500
H	0.94533800	-1.07973800	3.33647400
C	2.21219200	2.49640400	2.67403500
C	2.44934600	1.89245300	3.91109400
H	2.15615300	0.12423800	5.10970300
H	2.57414900	3.50281200	2.50644700
H	2.99108800	2.44016200	4.67650600
C	-1.30406100	0.28871700	0.49125300
C	-1.88644500	1.14770300	1.43412200
C	-2.10351100	-0.00650700	-0.64043500
C	-3.16424300	1.69794200	1.29167400
H	-1.31329900	1.39681700	2.32202200
C	-3.37958200	0.54153900	-0.81309100
C	-3.90912200	1.39566800	0.15719400
H	-3.56605800	2.35379200	2.05805400
H	-3.97680600	0.31023400	-1.68577400
H	-4.90251200	1.81119100	0.01635400
O	2.42099400	-2.24169400	0.61035700
O	1.25207900	2.35475400	0.43958400
O	-1.53550100	-0.85884700	-1.57037800
N	1.01179400	-0.19873100	-0.66062200

H	1.23721900	0.78359800	-0.82166100
H	1.87586500	-0.73968800	-0.61373500
H	0.44132300	-0.54258700	-1.43392400
C	1.53097000	3.74202900	0.15835200
H	2.52958400	3.98899700	0.53405700
C	3.65665000	-2.98640100	0.58985100
H	3.47016300	-3.97392600	0.15477900
C	-2.27574900	-1.32193400	-2.71920100
H	-2.81762000	-0.47724800	-3.15743500
C	4.22811700	-3.13544200	2.00269300
H	5.16151400	-3.70610500	1.96554600
H	3.52970100	-3.65805500	2.66094700
H	4.44029400	-2.15293700	2.43349100
C	0.48594000	4.65245600	0.80968700
H	0.71466800	5.69859600	0.58286800
H	0.47329100	4.52949000	1.89554000
H	-0.51150200	4.42421900	0.42355400
C	-3.25810600	-2.42813000	-2.32476400
H	-2.72031000	-3.28354000	-1.90631600
H	-3.81052600	-2.76453700	-3.20789700
H	-3.97690700	-2.07461500	-1.58140000
C	-1.25109100	-1.79287100	-3.73311400
C	-1.25809700	-1.27726900	-5.03241600
C	-0.29718300	-2.76279800	-3.39595100
C	-0.33477800	-1.72072700	-5.98235900
H	-1.99004300	-0.52226200	-5.30590900
C	0.63130300	-3.20108900	-4.33945200
H	-0.27768500	-3.17251000	-2.39097900
C	0.61394100	-2.68294900	-5.63700900
H	-0.35533900	-1.31003600	-6.98694000
H	1.36672300	-3.95020600	-4.06314500
H	1.33567300	-3.02659300	-6.37119700
C	1.55537800	3.87986100	-1.35144500
C	2.66190700	4.44890900	-1.98830900
C	0.46671600	3.46411600	-2.13007600
C	2.68438700	4.60741500	-3.37610900
H	3.51438300	4.77084900	-1.39651700
C	0.48998700	3.61287400	-3.51629700
H	-0.39887900	3.01787300	-1.65057100
C	1.59821300	4.18786300	-4.14384100
H	3.55151500	5.05243400	-3.85394900
H	-0.35882600	3.28310100	-4.10716400
H	1.61390500	4.30557700	-5.22269000
C	4.59675100	-2.24075100	-0.33737000
C	5.20936500	-2.90716200	-1.40250700
C	4.88547600	-0.88468100	-0.13261400
C	6.09815100	-2.23836700	-2.24770100
H	4.99044100	-3.95736400	-1.57498300
C	5.76536500	-0.21207400	-0.97983600
H	4.41620900	-0.35262900	0.68882600
C	6.37696700	-0.88781600	-2.03898700
H	6.56568500	-2.77187800	-3.06932000
H	5.97677100	0.83926200	-0.81135400
H	7.06370800	-0.36391700	-2.69619400

1c-c6

C	0.19457100	-4.59782200	1.28163100
C	0.49873800	-4.83924000	2.62316900
C	0.61999700	-3.77210300	3.50570700
C	0.43694500	-2.46973900	3.03080300
C	0.13154600	-2.16855400	1.69526000
C	0.01690700	-3.28565000	0.83240200
H	0.08141500	-5.43824900	0.60988900
H	0.63128400	-5.86172600	2.96393200
H	0.85318800	-3.94426200	4.55211500
H	0.53434500	-1.64691900	3.73223500
B	-0.12336900	-0.61480000	1.22127300
C	0.49437200	0.45749400	2.30382500
C	-0.35795200	1.13132300	3.19138100
C	1.87802600	0.70910500	2.47830100
C	0.09505800	1.99375700	4.19450100
H	-1.42680400	0.96512800	3.09911900
C	2.35494300	1.57831800	3.46649100
C	1.46044700	2.21654500	4.32868600
H	-0.61319100	2.48107800	4.85761200
H	3.41339400	1.77010800	3.58343400
H	1.84345500	2.88481900	5.09415000
C	-1.71142100	-0.31932100	0.91369700
C	-2.70741900	-1.14921300	1.44949700
C	-2.18131900	0.78712500	0.16502500
C	-4.07635000	-0.92579700	1.27155600
H	-2.39666100	-2.00895900	2.03504700
C	-3.54437400	1.03049000	-0.03235300
C	-4.49322500	0.16902000	0.52290600
H	-4.80215200	-1.59995000	1.71617800
H	-3.88276700	1.89209400	-0.59224900
H	-5.54942600	0.36966000	0.37022200
O	-0.28410100	-3.00249900	-0.49145100
O	2.73146000	0.03313200	1.62159000
O	-1.20761100	1.62144500	-0.36287900
N	0.67862800	-0.41208400	-0.18164200
H	0.38294800	0.46268400	-0.61536100
H	1.68104000	-0.38268800	0.00593400
H	0.46602500	-1.19357000	-0.80225300
C	4.16707400	0.11668400	1.77634600
H	4.39669000	0.12825000	2.84601200
C	-0.32812200	-4.04420300	-1.47751800
H	0.53359400	-4.70722700	-1.33277600
C	-1.56042000	2.73484000	-1.19670200
H	-2.31533900	2.41011200	-1.92335100
C	-0.18432200	-3.34445900	-2.83068600
H	-0.21135300	-4.08188600	-3.63759800
H	0.76774600	-2.80667500	-2.88569000
H	-1.00580200	-2.63764400	-2.98448600
C	4.71596900	1.38094200	1.11045400
H	5.80069000	1.43193900	1.24831200
H	4.27066600	2.28176300	1.53948000
H	4.50867700	1.37594700	0.03633800
C	-0.28463900	3.11040200	-1.95342000
H	0.50573200	3.39525600	-1.25186000
H	-0.47884400	3.95957000	-2.61429000
H	0.06220100	2.26991700	-2.56343700
C	-2.10347200	3.91269000	-0.40196300
C	-3.00239400	4.79872900	-1.00579400
C	-1.67714200	4.16515100	0.90572600
C	-3.46435100	5.92292100	-0.31987200
H	-3.34805600	4.60753600	-2.01877900
C	-2.14191300	5.28609400	1.59528100
H	-0.99338800	3.47547300	1.38900700
C	-3.03400800	6.16993300	0.98511100
H	-4.16356000	6.59944900	-0.80143900
H	-1.80747900	5.46650100	2.61221000
H	-3.39553100	7.04013500	1.52378400
C	4.74736000	-1.15833700	1.19401300
C	5.40420900	-2.06959000	2.02614600
C	4.64757600	-1.44843200	-0.17293800
C	5.95349400	-3.24491100	1.50920900
H	5.48468500	-1.86130900	3.08921500
C	5.18754900	-2.62416300	-0.69270300
H	4.14678100	-0.75425600	-0.84091500
C	5.84502200	-3.52594500	0.14757800
H	6.46008900	-3.93971900	2.17151900
H	5.10004100	-2.83454900	-1.75412000
H	6.26845300	-4.43954000	-0.25742500
C	-1.60840900	-4.86268000	-1.40878800
C	-1.59690700	-6.20140800	-1.81539800
C	-2.81703700	-4.28723000	-1.00385800
C	-2.77250800	-6.95356400	-1.82536900
H	-0.66148400	-6.66240100	-2.12298000
C	-3.99281800	-5.03915000	-1.00817800
H	-2.83439700	-3.25478600	-0.67114700
C	-3.97597100	-6.37279300	-1.42123500
H	-2.74628600	-7.99185100	-2.14125700
H	-4.92296700	-4.58149900	-0.68573600
H	-4.89130900	-6.95613000	-1.42233900

1c-c7

C	0.19918800	-4.46863000	1.24082300
C	0.51288700	-4.71553100	2.57911100
C	0.70221300	-3.64943000	3.45094600

C	0.57728800	-2.34313800	2.96842400
C	0.26594700	-2.03652500	1.63540900
C	0.08037900	-3.15233800	0.78400900
H	0.03237300	-5.30713300	0.57792400
H	0.59950800	-5.74093300	2.92587000
H	0.94352100	-3.82549800	4.49488800
H	0.72757000	-1.52078200	3.66111300
B	0.07505400	-0.47526000	1.15701300
C	0.77254400	0.57384200	2.21232700
C	-0.01846400	1.33351100	3.08657400
C	2.17254000	0.72778300	2.36677200
C	0.50758400	2.18833100	4.05996500
H	-1.09763300	1.24463100	3.00802400
C	2.72293000	1.58565500	3.32589800
C	1.88698000	2.31229900	4.17668200
H	-0.15549100	2.74798100	4.71267400
H	3.79380200	1.70447200	3.42572200
H	2.32630600	2.97290500	4.91822900
C	-1.50575200	-0.10861000	0.89370000
C	-2.51817500	-0.87825900	1.48573500
C	-1.94870200	1.01271600	0.14923300
C	-3.88028800	-0.58308900	1.37274800
H	-2.22520800	-1.74417800	2.07130700
C	-3.30724000	1.32019100	0.00917300
C	-4.27291400	0.52282100	0.62776800
H	-4.61847800	-1.21178800	1.86129700
H	-3.63394800	2.17150900	-0.57337500
H	-5.32325200	0.77467300	0.51542100
O	-0.22941600	-2.86412600	-0.53693000
O	2.96427600	-0.02781100	1.51671400
O	-0.95693900	1.79155400	-0.42433200
N	0.85246300	-0.31402200	-0.26529600
H	0.59375300	0.57562600	-0.69213500
H	1.85886900	-0.33698700	-0.09924100
H	0.58731600	-1.08295300	-0.88124500
C	4.39944300	-0.08185500	1.68708500
H	4.61770200	-0.05633100	2.75891300
C	-0.33607600	-3.90959500	-1.51372200
H	0.49600200	-4.61087000	-1.37609900
C	-1.27484700	3.04785600	-1.06755300
H	-2.08942900	3.52056600	-0.51091700
C	-0.17963600	-3.22744300	-2.87441900
H	-0.25373900	-3.96834600	-3.67518400
H	0.79620600	-2.73639800	-2.94708800
H	-0.96854600	-2.48311300	-3.02165000
C	5.07599600	1.10030100	0.98919700
H	6.15893300	1.05199800	1.14199800
H	4.71281600	2.05315600	1.38142900
H	4.88209600	1.07951400	-0.08717800
C	-1.69877000	2.82326400	-2.52093400
H	-0.88899600	2.36565600	-3.09677900
H	-1.94959200	3.78133900	-2.98730500
H	-2.57232700	2.16971600	-2.58295600
C	-0.05196900	3.93638200	-0.93593700
C	-0.11387100	5.09546500	-0.15659600
C	1.15001100	3.62832100	-1.58611500
C	0.99623800	5.93289500	-0.02813400
H	-1.03715400	5.34490900	0.35844800
C	2.26354000	4.45715400	-1.45504600
H	1.22228800	2.73693700	-2.20159500
C	2.18915600	5.61470900	-0.67662800
H	0.92796800	6.82853100	0.58118200
H	3.18794500	4.20263500	-1.96415300
H	3.05483300	6.26180700	-0.57729000
C	4.85909500	-1.42701000	1.15619600
C	5.38654700	-2.37898700	2.03383700
C	4.77547900	-1.74392400	-0.20582900
C	5.82415800	-3.62013400	1.56674800
H	5.45302800	-2.15024900	3.09364400
C	5.20446000	-2.98438600	-0.67636000
H	4.37537200	-1.01987800	-0.90887700
C	5.73291400	-3.92660600	0.20932100
H	6.23097800	-4.34551300	2.26418100
H	5.13136400	-3.21410200	-1.73488100
H	6.07016400	-4.89089100	-0.15734800
C	-1.65138100	-4.66786000	-1.41962500
C	-1.70863900	-6.00711400	-1.82067900
C	-2.82457500	-4.03644700	-0.99469900
C	-2.91741300	-6.70459800	-1.80583300
H	-0.80096800	-6.51159100	-2.14291400
C	-4.03350400	-4.73357500	-0.97427400
H	-2.78785500	-3.00345700	-0.66531600
C	-4.08538000	-6.06791500	-1.38212600
H	-2.94457300	-7.74411500	-2.11757800
H	-4.93549700	-4.23278800	-0.63633400
H	-5.02627700	-6.60875200	-1.36385900

1c-c8

C	0.33500800	-4.53920900	1.38572800
C	0.63689900	-4.73643300	2.73500900
C	0.73311300	-3.64219000	3.58721700
C	0.53174700	-2.35746300	3.07298000
C	0.22949100	-2.09985700	1.72740100
C	0.13411500	-3.24386700	0.89769500
H	0.23877600	-5.40089800	0.73875200
H	0.78484600	-5.74614800	3.10607300
H	0.95973300	-3.78000400	4.64015100
H	0.61074200	-1.51247600	3.74998200
B	-0.04633300	-0.56229300	1.21306900
C	0.58315900	0.54876700	2.24876800
C	-0.25461000	1.28298600	3.10340100
C	1.96898200	0.79724300	2.39453800
C	0.21969200	2.20137600	4.04406100
H	-1.32601600	1.12129300	3.03461800
C	2.46613700	1.73172300	3.30869600
C	1.58899400	2.42869100	4.14072000
H	-0.47524200	2.73246300	4.68757000
H	3.52817600	1.92585500	3.37470900
H	1.98504300	3.14626800	4.85311700
C	-1.64140200	-0.28926700	0.92280100
C	-2.62154900	-1.11188400	1.49765900
C	-2.13281900	0.79083200	0.14934800
C	-3.99505800	-0.90585900	1.33453000
H	-2.29437300	-1.95151200	2.10314800
C	-3.50102100	1.01597400	-0.03394600
C	-4.43327400	0.16278300	0.56075700
H	-4.70771400	-1.57312900	1.80973700
H	-3.85588800	1.85783400	-0.61332300
H	-5.49347800	0.34956400	0.41862100
O	-0.17255200	-3.00611900	-0.43345900
O	2.80540900	0.08504100	1.54615200
O	-1.17534100	1.61814700	-0.41744400
N	0.73335800	-0.38899600	-0.20784400
H	0.41716000	0.46802700	-0.66221400
H	1.73776200	-0.33650900	-0.03325600
H	0.52757500	-1.19066600	-0.80432900
C	4.19454800	-0.18705000	1.84721300
H	4.43758500	-0.91414300	1.06324900
C	-0.21812200	-4.07973400	-1.38525400
H	0.65384000	-4.72755600	-1.23239300
C	-1.55112000	2.69723400	-1.28639400
H	-2.31201200	2.33801900	-1.99014400
C	-0.10225200	-3.42168200	-2.76163100
H	-0.12871300	-4.18502400	-3.54401700
H	0.84087100	-2.87211200	-2.84645300
H	-0.93602100	-2.73209200	-2.92685100
C	4.36663800	-0.90519700	3.18510200
H	5.36174000	-1.35646000	3.23623900
H	3.62917700	-1.70880800	3.25362500
H	4.24371000	-0.24545500	4.04662000
C	-0.28935500	3.05814900	-2.07292300
H	0.50612800	3.37816200	-1.39269600
H	-0.50173900	3.87957200	-2.76265900
H	0.05957200	2.19910900	-2.65522000
C	-2.09611200	3.89757900	-0.52772500
C	-3.02995000	4.73904800	-1.14203400
C	-1.63689800	4.21522400	0.75448300
C	-3.49480100	5.88308200	-0.49165900
H	-3.40064500	4.49758100	-2.13522700
C	-2.10440200	5.35595800	1.40866000

H	-0.92462300	3.56120400	1.24619900
C	-3.03197500	6.19493200	0.78800400
H	-4.22130700	6.52444700	-0.98088100
H	-1.74381400	5.58742800	2.40611500
H	-3.39533600	7.08076600	1.29926200
C	5.09099300	1.02659200	1.60669400
C	4.82320500	1.87720200	0.52479400
C	6.22181300	1.28295900	2.38976300
C	5.65435000	2.95864700	0.24007600
H	3.94807800	1.69527900	-0.09006700
C	7.05985500	2.36486100	2.10455900
H	6.45924400	0.64470200	3.23325700
C	6.77954200	3.20735100	1.03020300
H	5.42394500	3.60804200	-0.59885900
H	7.93026100	2.54627800	2.72746200
H	7.42811500	4.04922400	0.81002500
C	-1.48672400	-4.91094300	-1.27049500
C	-1.46402700	-6.26233700	-1.63206000
C	-2.69635700	-4.33696700	-0.86665300
C	-2.62978200	-7.02896600	-1.59806600
H	-0.52759100	-6.72174900	-1.93888900
C	-3.86216900	-5.10319400	-0.82684900
H	-2.72210900	-3.29405700	-0.56912100
C	-3.83426000	-6.44984700	-1.19460500
H	-2.59496900	-8.07692000	-1.87925000
H	-4.79313700	-4.64643300	-0.50557000
H	-4.74180400	-7.04429100	-1.16136800

1c-c9

C	2.58801600	-2.96303500	-1.00857400
C	3.54564900	-2.37593000	-1.83841000
C	3.45648500	-1.02179400	-2.14335200
C	2.40826100	-0.26761200	-1.60688000
C	1.41594100	-0.80681400	-0.77436100
C	1.54074500	-2.18896600	-0.49868900
H	2.67581800	-4.01229300	-0.75747900
H	4.35498400	-2.98314400	-2.23270900
H	4.19246400	-0.55195100	-2.78885900
H	2.35185500	0.78783100	-1.85503000
B	0.16972500	0.11914800	-0.23755400
C	-1.24514400	-0.25642900	-0.98415800
C	-1.24752400	-0.94767300	-2.20508900
C	-2.51872000	0.13010900	-0.49810400
C	-2.41158100	-1.25083800	-2.91709800
H	-0.29325000	-1.25963100	-2.61854500
C	-3.69704900	-0.16021700	-1.19322800
C	-3.64149500	-0.85375200	-2.40360400
H	-2.35270000	-1.78722600	-3.85937200
H	-4.65690000	0.16512100	-0.81831500
H	-4.56307900	-1.07006500	-2.93573100
C	0.51400200	1.72100900	-0.34834900
C	-0.07039700	2.51023800	-1.35029100
C	1.45309000	2.38440700	0.47651500
C	0.23762400	3.86028200	-1.54531400
H	-0.79112900	2.04338900	-2.01470200
C	1.76591000	3.73807600	0.31482300
C	1.15881000	4.47518300	-0.70425700
H	-0.23966600	4.41946100	-2.34442500
H	2.46610000	4.23015100	0.97712500
H	1.40917300	5.52483700	-0.82548800
O	0.56421500	-2.73972900	0.31674300
O	-2.51058900	0.83631100	0.69511700
O	2.05131600	1.61372800	1.46132900
N	-0.02840700	-0.22376600	1.34503100
H	-0.90448900	0.19205000	1.66346800
H	-0.06430000	-1.23591100	1.46829900
H	0.75198400	0.16171600	1.87700200
C	-3.70463400	1.19537100	1.42871000
H	-3.26578700	1.71820800	2.28696400
C	0.34835700	-4.15838900	0.34024000
H	0.49991900	-4.54700400	-0.67381400
C	3.23370000	2.07270500	2.13254600
H	3.87825000	2.56901800	1.39711700
C	-1.11775500	-4.35502100	0.73124600
H	-1.36304700	-5.42079400	0.74237300
H	-1.77009900	-3.84718500	0.01537700
H	-1.30449100	-3.95235400	1.73226900
C	-4.43434100	-0.02774200	1.98796800
H	-5.13247000	0.28366700	2.77051500
H	-3.70408800	-0.70104900	2.44564500
H	-4.99183800	-0.58641000	1.23380800
C	3.94263300	0.81689100	2.64327600
H	3.31013200	0.28521800	3.36184300
H	4.87265100	1.09239400	3.14845900
H	4.17613900	0.14809700	1.81045900
C	2.93384600	3.04246200	3.26667600
C	3.89028800	3.99794900	3.62828400
C	1.74576900	2.96764500	4.00011200
C	3.66875400	4.85854900	4.70424600
H	4.81466300	4.07360500	3.06117200
C	1.51836200	3.83001300	5.07374800
H	0.99004300	2.24055800	3.72251800
C	2.47974800	4.77696900	5.43128900
H	4.42058800	5.59553300	4.96879500
H	0.58784900	3.76376000	5.62908600
H	2.30158900	5.44878900	6.26483700
C	-4.55806100	2.22400300	0.69151900
C	-3.92533000	3.24327300	-0.03397600
C	-5.95448200	2.23005500	0.77494700
C	-4.67056900	4.23582700	-0.66701700
H	-2.84311400	3.24843600	-0.11071800
C	-6.70358200	3.22727400	0.14369500
H	-6.47399500	1.45686800	1.32993600
C	-6.06518700	4.23276300	-0.58036800
H	-4.16157300	5.01328400	-1.22823500
H	-7.78647900	3.21237400	0.21919500
H	-6.64604600	5.00608500	-1.07291200
C	1.28436200	-4.88334700	1.29650000
C	1.62562400	-6.21804300	1.04975500
C	1.77138100	-4.26646000	2.45302400
C	2.43223800	-6.92560800	1.94206000
H	1.26258800	-6.70778800	0.14970200
C	2.58209100	-4.96951200	3.34555700
H	1.52618200	-3.22815400	2.64951800
C	2.91359700	-6.30220300	3.09474700
H	2.68922400	-7.95938600	1.73311600
H	2.95721000	-4.47432400	4.23591200
H	3.54577300	-6.84787900	3.78790400

1c-c10

C	-0.11473700	-4.39382600	0.76946600
C	0.07353900	-4.74325200	2.10871200
C	0.26823700	-3.74944200	3.06055300
C	0.26871900	-2.41115700	2.65587800
C	0.07576500	-2.00276100	1.32821600
C	-0.11754900	-3.04660000	0.39008000
H	-0.25557900	-5.18374800	0.04352300
H	0.07177500	-5.79203200	2.39045200
H	0.41864100	-4.00537000	4.10498400
H	0.41641500	-1.64435600	3.41006700
B	-0.01090300	-0.40543100	0.95163200
C	0.66402000	0.53349400	2.12016200
C	-0.14766900	1.26636400	2.99846100
C	2.05638000	0.60799200	2.37115900
C	0.35116500	2.02325200	4.06290800
H	-1.22220800	1.23539600	2.84714400
C	2.58054100	1.36625600	3.42445000
C	1.72412400	2.07044000	4.27378600
H	-0.32763800	2.56688700	4.71300300
H	3.64675200	1.42447800	3.59939700
H	2.14313800	2.65381900	5.08829000
C	-1.55591100	0.05015200	0.62474700
C	-2.63315400	-0.71314700	1.09853600
C	-1.90633600	1.24004200	-0.05942900
C	-3.97231900	-0.34851800	0.93063000
H	-2.41270200	-1.63192700	1.63304500
C	-3.23963700	1.62111100	-0.24947200
C	-4.27296300	0.82681800	0.25240100
H	-4.76433600	-0.97831100	1.32437400
H	-3.49513700	2.52667300	-0.78368900

H	-5.30321400	1.13461600	0.10014600
O	-0.32412200	-2.65137400	-0.92240200
O	2.86790100	-0.12093100	1.51642100
O	-0.84986600	2.00962200	-0.52061200
N	0.85791200	-0.18392200	-0.40727100
H	0.66451000	0.74525100	-0.78135000
H	1.85065300	-0.26865000	-0.18797600
H	0.59286700	-0.89294100	-1.09137100
C	4.28454600	-0.25791300	1.77265500
H	4.43091600	-0.32501800	2.85491600
C	-0.62383400	-3.62133500	-1.95183600
H	-1.28887100	-4.37881300	-1.52624300
C	-1.07924600	3.30979700	-1.10937000
H	-1.89774000	3.79216700	-0.56670300
C	0.65508200	-4.28347900	-2.47084900
H	0.40389600	-5.02946200	-3.23168700
H	1.20032400	-4.78046700	-1.66517200
H	1.31958000	-3.54324100	-2.92583500
C	5.05901700	0.93908700	1.21591600
H	6.12524800	0.82707300	1.43753500
H	4.71041300	1.87648700	1.65563000
H	4.94189100	1.00856900	0.13060000
C	-1.43954300	3.18367400	-2.59197800
H	-0.61771900	2.73055500	-3.15406200
H	-1.63360100	4.17575900	-3.01224300
H	-2.33012700	2.56692500	-2.73366600
C	0.17475300	4.13143700	-0.87694800
C	0.12227500	5.27268400	-0.07126000
C	1.39668900	3.78148100	-1.46598300
C	1.26121400	6.05221500	0.14233600
H	-0.81672900	5.55422100	0.39675800
C	2.53849600	4.55161400	-1.24930700
H	1.46192800	2.90281900	-2.10052400
C	2.47349800	5.69247400	-0.44562800
H	1.19981100	6.93508600	0.77074200
H	3.47805600	4.26483000	-1.71138600
H	3.36141700	6.29424600	-0.27986600
C	4.71878100	-1.57978500	1.16725000
C	5.13619400	-2.62304600	1.99921500
C	4.71925900	-1.78565200	-0.21845000
C	5.54840000	-3.84541400	1.46454600
H	5.13618400	-2.48055000	3.07607100
C	5.12277700	-3.00686300	-0.75705700
H	4.40519100	-0.98962100	-0.88658100
C	5.54168900	-4.04080700	0.08370500
H	5.86960000	-4.64268800	2.12733800
H	5.11555600	-3.15025300	-1.83312600
H	5.85952700	-4.99006200	-0.33572600
C	-1.39373200	-2.89264200	-3.03726400
C	-2.72772100	-3.22651300	-3.28953400
C	-0.79544600	-1.88964600	-3.81095200
C	-3.45146100	-2.57869000	-4.29292600
H	-3.20725100	-3.99842700	-2.69438900
C	-1.51582100	-1.23447700	-4.80883000
H	0.24074800	-1.61530300	-3.63831900
C	-2.84693700	-1.57946400	-5.05508400
H	-4.48591800	-2.85297900	-4.47394100
H	-1.03746700	-0.45831400	-5.39808900
H	-3.40659000	-1.07226800	-5.83453600

Conformers of 1d

1d-c1

B	0.07537	0.36807	-0.35904
C	0.81284	-1.09881	-0.35419
C	0.80200	-1.91618	-1.49318
C	1.53678	-1.60663	0.75238
C	1.44905	-3.15479	-1.55991
H	0.26431	-1.56695	-2.36945
C	2.18203	-2.84798	0.71655
C	2.13781	-3.62166	-0.44629
H	1.40912	-3.74339	-2.47147
H	2.72186	-3.22124	1.57729
H	2.64425	-4.58209	-0.46765
O	1.57354	-0.80602	1.87785
N	-0.75592	0.48385	1.04412
H	-1.57011	-0.13050	1.00597
H	-1.06206	1.44859	1.17588
H	-0.15081	0.20648	1.81786
C	-1.01901	0.50242	-1.57522
C	-2.25360	-0.19120	-1.60366
C	-0.74720	1.29416	-2.69991
C	-3.14736	-0.08070	-2.67497
C	-1.61698	1.41224	-3.78913
H	0.18987	1.84203	-2.72617
C	-2.82408	0.72294	-3.77135
H	-4.08950	-0.61345	-2.67262
H	-1.35058	2.03809	-4.63549
H	-3.52123	0.79995	-4.60045
C	1.15867	1.60133	-0.36572
C	0.82081	2.93871	-0.04457
C	2.49218	1.39042	-0.74245
C	1.75905	3.97633	-0.08546
C	3.44687	2.41119	-0.80370
H	2.79771	0.38156	-1.00269
C	3.07579	3.70847	-0.46919
H	1.48161	4.99015	0.17305
H	4.46557	2.18888	-1.10672
H	3.79728	4.51949	-0.50230
O	-0.49364	3.16710	0.31485
C	2.32670	-1.22571	3.01739
H	1.91065	-2.16522	3.40514
H	3.36950	-1.40824	2.72247
C	-0.92680	4.50063	0.59033
H	-0.38013	4.89072	1.45937
H	-0.70509	5.14490	-0.27183
O	-2.53388	-0.99024	-0.51093
C	-3.73961	-1.75698	-0.48957
H	-4.60384	-1.08066	-0.53049
H	-3.77461	-2.41217	-1.37114
C	2.26476	-0.13652	4.08926
C	2.97044	1.15150	3.62353
H	2.63300	1.39060	2.60946
H	4.04916	0.95218	3.54957
C	2.73499	2.36676	4.52804
H	3.13339	2.21745	5.53712
H	3.21930	3.25691	4.11287
H	1.66436	2.58653	4.62270
C	2.84976	-0.69085	5.39737
H	3.90694	-0.95683	5.27234
H	2.78668	0.04335	6.20507
H	2.31407	-1.58883	5.72477
H	1.20194	0.09295	4.25929
C	-2.43033	4.49257	0.86957
H	-2.61150	3.74235	1.65396
C	-3.23245	4.08914	-0.38251
H	-2.77393	3.19695	-0.82221
H	-3.13432	4.88699	-1.13265
C	-4.71748	3.81094	-0.12228
H	-5.21537	3.48128	-1.04027
H	-4.84519	3.01885	0.62594
H	-5.24947	4.69740	0.23893
C	-2.84245	5.86745	1.41790
H	-3.90548	5.89320	1.67239
H	-2.27985	6.12040	2.32343
H	-2.65915	6.65586	0.67705
C	-3.78233	-2.59206	0.79102
H	-3.62275	-1.90317	1.63422
C	-2.66284	-3.65035	0.81135
H	-2.87267	-4.39698	0.03209
H	-1.71982	-3.17015	0.52934
C	-2.47992	-4.35501	2.16058
H	-3.36574	-4.92827	2.45362
H	-1.63666	-5.05260	2.12099
H	-2.27344	-3.63122	2.95874
C	-5.18182	-3.20938	0.93623
H	-5.95878	-2.43693	0.94739
H	-5.39779	-3.89156	0.10438
H	-5.27158	-3.78035	1.86430

1d-c2

B	-0.34700	-0.45596	-0.85028
C	0.83146	-0.99768	-1.85752
C	2.16212	-0.58839	-1.69032
C	0.59033	-1.84417	-2.96745
C	3.20155	-0.97523	-2.54279
H	2.39569	0.06542	-0.85552
C	1.61420	-2.25618	-3.82816
C	2.92317	-1.81674	-3.61365
H	4.21326	-0.62330	-2.36512
H	1.40928	-2.90985	-4.66608
H	3.71107	-2.13785	-4.28853
O	-0.71846	-2.24462	-3.16082
N	-1.28689	-1.74076	-0.48222
H	-1.52177	-2.24286	-1.33916
H	-2.14159	-1.41508	-0.02919
H	-0.77879	-2.36030	0.15001
C	-1.31721	0.64897	-1.58117
C	-2.58950	1.02425	-1.08439
C	-0.89649	1.32705	-2.73380
C	-3.37940	1.99476	-1.71093
C	-1.66067	2.30949	-3.37262
H	0.07463	1.07698	-3.15000
C	-2.90929	2.63948	-2.85820
H	-4.35269	2.26028	-1.31867
H	-1.28134	2.80411	-4.26169
H	-3.52638	3.39415	-3.33681
C	0.26634	0.10666	0.56533
C	0.83275	-0.72542	1.56194
C	0.30586	1.48187	0.83523
C	1.37914	-0.20996	2.74278
C	0.85705	2.02403	2.00113
H	-0.11238	2.16047	0.09796
C	1.39175	1.17050	2.95930
H	1.79898	-0.86483	3.49542
H	0.86251	3.09900	2.15408
H	1.82098	1.56278	3.87649
O	0.81870	-2.08361	1.30676
C	-1.04299	-3.07409	-4.27871
H	-0.73681	-2.57380	-5.20799
H	-0.49417	-4.02216	-4.20138
C	1.42515	-2.98289	2.23724
H	2.47534	-2.69818	2.39117
H	0.90553	-2.91619	3.20246
O	-3.01324	0.37771	0.06090
C	-4.26349	0.73634	0.65287
H	-4.26744	1.81164	0.87935
H	-5.07637	0.53095	-0.05646
C	-2.55012	-3.33344	-4.28925
C	-2.86806	-4.40864	-5.34724
H	-2.20606	-5.26951	-5.18511
H	-2.62162	-4.01498	-6.34352
C	-4.32068	-4.89832	-5.33917
H	-5.02494	-4.10090	-5.59632
H	-4.45967	-5.70594	-6.06561
H	-4.60048	-5.28678	-4.35243
C	-3.33904	-2.03467	-4.50821
H	-3.15339	-1.63691	-5.51414
H	-4.41607	-2.19743	-4.40689
H	-3.04529	-1.26709	-3.78727
H	-2.81521	-3.74565	-3.30382
C	1.33784	-4.40640	1.68507
H	0.27784	-4.60412	1.46462
C	1.78232	-5.39799	2.77838
H	1.22826	-5.17785	3.70045
H	2.84314	-5.22513	3.00868
C	1.57398	-6.87399	2.42082
H	1.84570	-7.51746	3.26428
H	0.52479	-7.07566	2.17310
H	2.18410	-7.17960	1.56505
C	2.14553	-4.55239	0.38764
H	1.99855	-5.53618	-0.06771
H	1.85583	-3.79506	-0.34578
H	3.21815	-4.43213	0.58654
C	-4.45903	-0.07572	1.93399
H	-4.34973	-1.13709	1.66424
C	-5.89748	0.13202	2.44758
H	-6.02744	1.18555	2.73303
H	-6.59677	-0.05040	1.62090
C	-6.28721	-0.76583	3.62739
H	-5.68733	-0.55695	4.51873
H	-7.33810	-0.61527	3.89621
H	-6.15659	-1.82554	3.37677
C	-3.39665	0.27586	2.98512
H	-2.38792	0.18269	2.57382
H	-3.52338	1.31035	3.32917
H	-3.46594	-0.37784	3.85957

1d-c3

B	-0.28098	0.43787	0.17510
C	0.60481	-0.94256	0.26045
C	1.93219	-0.97242	-0.19016
C	0.09254	-2.17856	0.72536
C	2.72187	-2.12713	-0.18984
H	2.36794	-0.04960	-0.56084
C	0.86465	-3.34566	0.74754
C	2.18314	-3.31711	0.28557
H	3.74396	-2.08994	-0.55441
H	0.45536	-4.27841	1.11341
H	2.77415	-4.22804	0.30254
O	-1.22040	-2.17424	1.15713
N	-1.08848	0.57436	1.58857
H	-1.52778	-0.31895	1.81381
H	-1.80445	1.29632	1.49935
H	-0.43311	0.83109	2.32780
C	-1.41152	0.36786	-1.01410
C	-2.52201	1.24361	-1.09513
C	-1.29582	-0.55646	-2.06193
C	-3.44844	1.17933	-2.14211
C	-2.20066	-0.63402	-3.12606
H	-0.45727	-1.24601	-2.04612
C	-3.28324	0.23731	-3.16096
H	-4.29443	1.85340	-2.18080
H	-2.05763	-1.36925	-3.91215
H	-4.00453	0.19649	-3.97179
C	0.67331	1.76738	0.03840
C	1.47846	2.26862	1.09058
C	0.78091	2.45183	-1.18044
C	2.31276	3.38036	0.92777
C	1.61537	3.55803	-1.37329
H	0.18614	2.10181	-2.01875
C	2.38079	4.02416	-0.31064
H	2.91530	3.75046	1.74725
H	1.65996	4.04615	-2.34218
H	3.03368	4.88373	-0.42998
O	1.40002	1.59586	2.29521
C	-1.81356	-3.39029	1.61792
H	-1.76172	-4.14795	0.82363
H	-1.25413	-3.76303	2.48628
C	2.20623	2.02317	3.39610
H	3.26655	1.99321	3.10827
H	1.94814	3.05759	3.65164
O	-2.64441	2.17346	-0.07995
C	-3.72032	3.11383	-0.11099
H	-3.67970	3.68700	-1.04774
H	-4.67713	2.57576	-0.07795
C	-3.26986	-3.12320	2.00057
C	-3.83792	-4.36481	2.71624
H	-3.14875	-4.65396	3.52066
H	-3.85752	-5.20756	2.01078
C	-5.23604	-4.16996	3.31388
H	-5.98961	-3.97914	2.54338
H	-5.54838	-5.06372	3.86428
H	-5.25174	-3.32587	4.01396
C	-4.10166	-2.71064	0.77803
H	-4.18280	-3.54431	0.06887
H	-5.11546	-2.41481	1.06342
H	-3.64110	-1.86991	0.25257
H	-3.26796	-2.29252	2.72257
C	1.96023	1.08674	4.57989
H	0.87514	1.06449	4.76102

C	2.64033	1.62533	5.85541
H	3.70755	1.79176	5.65064
H	2.60187	0.83354	6.61373
C	2.02033	2.89898	6.44391
H	2.51603	3.17142	7.38180
H	2.10757	3.75733	5.76982
H	0.95552	2.75350	6.66193
C	2.43350	-0.33999	4.26745
H	2.18301	-1.01980	5.08911
H	1.97675	-0.72530	3.35190
H	3.52233	-0.36530	4.13388
C	-3.59821	4.05261	1.09012
H	-3.55168	3.42207	1.99093
C	-4.87249	4.91460	1.18710
H	-4.93450	5.56355	0.30207
H	-5.74879	4.25431	1.14554
C	-4.96416	5.77288	2.45395
H	-4.16911	6.52369	2.50025
H	-5.92067	6.30485	2.49132
H	-4.89264	5.15338	3.35614
C	-2.31686	4.89462	1.01254
H	-1.43590	4.26138	0.87815
H	-2.36291	5.58816	0.16314
H	-2.17053	5.48732	1.92031

1d-c4

B	-0.31504	0.46023	0.18819
C	0.58415	-0.90964	0.29567
C	1.92774	-0.92324	-0.10420
C	0.06822	-2.15246	0.73777
C	2.72975	-2.06926	-0.07611
H	2.36682	0.00525	-0.45645
C	0.85176	-3.31105	0.78677
C	2.18663	-3.26649	0.37565
H	3.76437	-2.01998	-0.40192
H	0.43914	-4.24929	1.13449
H	2.78678	-4.17082	0.41282
O	-1.25980	-2.16243	1.11991
N	-1.15387	0.58401	1.58408
H	-1.59290	-0.31291	1.79549
H	-1.87229	1.30215	1.48450
H	-0.51528	0.83813	2.33801
C	-1.41644	0.37705	-1.02623
C	-2.53885	1.23485	-1.12989
C	-1.26105	-0.54121	-2.07426
C	-3.43967	1.15953	-2.19827
C	-2.13948	-0.62916	-3.15948
H	-0.41185	-1.21703	-2.04149
C	-3.23526	0.22443	-3.21635
H	-4.29575	1.81953	-2.25387
H	-1.96621	-1.35874	-3.94476
H	-3.93682	0.17484	-4.04383
C	0.62472	1.80174	0.07884
C	1.42142	2.28894	1.14361
C	0.73285	2.50901	-1.12712
C	2.25425	3.40482	1.00222
C	1.56337	3.62161	-1.29723
H	0.14481	2.16955	-1.97440
C	2.32479	4.07055	-0.22414
H	2.85038	3.76359	1.83145
H	1.61004	4.12662	-2.25732
H	2.97561	4.93391	-0.32614
O	1.33519	1.60312	2.34143
C	-1.85616	-3.38462	1.55992
H	-1.76702	-4.14236	0.76901
H	-1.32482	-3.75062	2.44861
C	2.28366	1.88272	3.37480
H	3.29557	1.78565	2.96188
H	2.15650	2.91445	3.73328
O	-2.69988	2.15845	-0.11442
C	-3.78244	3.08978	-0.17261
H	-3.72412	3.66177	-1.10913
H	-4.73541	2.54399	-0.16143
C	-3.32836	-3.13300	1.88850
C	-3.90750	-4.37812	2.58910
H	-3.24402	-4.65618	3.41863
H	-3.89270	-5.22386	1.88709
C	-5.32794	-4.19682	3.13627
H	-6.05598	-4.01745	2.33894
H	-5.64946	-5.09197	3.67908
H	-5.37772	-3.35033	3.83184
C	-4.12039	-2.73527	0.63496
H	-4.16670	-3.57317	-0.07239
H	-5.14708	-2.45006	0.88261
H	-3.65122	-1.89147	0.12203
H	-3.36193	-2.29940	2.60624
C	2.08606	0.89829	4.52852
H	2.06426	-0.10974	4.09180
C	0.75342	1.14257	5.26295
H	-0.04896	1.24859	4.52335
H	0.81152	2.10787	5.78595
C	0.36128	0.04177	6.25548
H	-0.61619	0.25203	6.70281
H	0.29725	-0.93304	5.75732
H	1.08194	-0.05055	7.07429
C	3.29469	0.99177	5.47300
H	3.21194	0.28055	6.29924
H	4.22843	0.77468	4.94325
H	3.37874	1.99650	5.90636
C	-3.69649	4.03154	1.02939
H	-3.66770	3.40326	1.93251
C	-4.97889	4.88455	1.09313
H	-5.02341	5.53189	0.20586
H	-5.84914	4.21794	1.03063
C	-5.10841	5.74374	2.35604
H	-4.31999	6.50012	2.42130
H	-6.06924	6.26906	2.36858
H	-5.05527	5.12587	3.26060
C	-2.41957	4.88254	0.98185
H	-1.53078	4.25554	0.87159
H	-2.44911	5.57361	0.12967
H	-2.30059	5.47851	1.89146

1d-c5

B	-0.27247	0.42441	0.17391
C	0.62794	-0.94294	0.30253
C	1.96068	-0.96759	-0.13209
C	0.12573	-2.17272	0.79427
C	2.76492	-2.11150	-0.09156
H	2.38914	-0.04931	-0.52209
C	0.91245	-3.32851	0.85710
C	2.23582	-3.29521	0.40963
H	3.79066	-2.07088	-0.44535
H	0.51097	-4.25624	1.24381
H	2.83822	-4.19749	0.45826
O	-1.19225	-2.17417	1.21081
N	-1.09474	0.58533	1.57623
H	-1.52695	-0.30705	1.81835
H	-1.81749	1.29707	1.46305
H	-0.44898	0.86576	2.31527
C	-1.38906	0.31334	-1.02501
C	-2.51048	1.17171	-1.13649
C	-1.24718	-0.62896	-2.05345
C	-3.42193	1.07522	-2.19404
C	-2.13742	-0.73946	-3.12699
H	-0.39928	-1.30606	-2.01417
C	-3.23089	0.11635	-3.19245
H	-4.27547	1.73779	-2.25726
H	-1.97433	-1.48736	-3.89706
H	-3.94068	0.05060	-4.01175
C	0.66682	1.76225	0.01478
C	1.46058	2.29321	1.06107
C	0.77137	2.42516	-1.21618
C	2.28196	3.41192	0.88099
C	1.59276	3.53806	-1.42601
H	0.18491	2.05210	-2.05033
C	2.34766	4.03323	-0.36900
H	2.87616	3.80489	1.69590
H	1.63562	4.00864	-2.40360
H	2.99052	4.89839	-0.50159

O	1.38409	1.64243	2.27772
C	-1.77523	-3.38507	1.69752
H	-1.69967	-4.16472	0.92680
H	-1.22457	-3.72401	2.58516
C	2.17823	2.10170	3.37448
H	3.24051	2.08050	3.09320
H	1.90493	3.13732	3.60856
O	-2.65782	2.11949	-0.14146
C	-3.74648	3.04455	-0.20409
H	-3.69564	3.60796	-1.14646
H	-4.69212	2.49019	-0.18521
C	-3.24144	-3.12859	2.04837
C	-3.80253	-4.35520	2.79470
H	-3.12246	-4.60771	3.61895
H	-3.79796	-5.22032	2.11658
C	-5.21306	-4.16294	3.36333
H	-5.95667	-4.00886	2.57513
H	-5.52088	-5.04320	3.93749
H	-5.25295	-3.29700	4.03519
C	-4.05923	-2.76904	0.79980
H	-4.11712	-3.62711	0.11794
H	-5.08159	-2.47886	1.05924
H	-3.60246	-1.93961	0.25350
H	-3.26389	-2.27509	2.74288
C	1.93815	1.18493	4.57470
H	0.85219	1.14855	4.74806
C	2.59965	1.75979	5.84411
H	3.66479	1.94280	5.64270
H	2.57140	0.98175	6.61696
C	1.95169	3.03211	6.40473
H	2.43729	3.33274	7.33927
H	2.02505	3.87867	5.71424
H	0.88893	2.86968	6.62080
C	2.43686	-0.23970	4.29373
H	2.18937	-0.90795	5.12572
H	1.99511	-0.64935	3.38136
H	3.52722	-0.25000	4.17086
C	-3.64818	4.00369	0.98294
H	-3.59108	3.39228	1.89591
C	-4.91181	4.88280	1.08292
H	-4.71135	5.67287	1.81713
H	-5.06801	5.39794	0.12438
C	-6.19448	4.14724	1.49128
H	-6.06341	3.63035	2.44950
H	-7.02384	4.85322	1.60669
H	-6.50360	3.40205	0.75104
C	-2.38375	4.86998	0.89438
H	-1.48188	4.25926	0.79954
H	-2.43078	5.53413	0.02215
H	-2.28139	5.49811	1.78599

1d-c6

B	-0.68210	5.85597	3.60680
C	-0.75466	5.78731	1.96779
C	-1.78104	6.43661	1.26676
C	0.14573	5.03754	1.17201
C	-1.93283	6.36461	-0.12191
H	-2.49866	7.02074	1.83476
C	0.01975	4.95403	-0.21943
C	-1.02501	5.61956	-0.86574
H	-2.75130	6.88531	-0.60970
H	0.72111	4.37768	-0.80896
H	-1.11663	5.54737	-1.94544
O	1.16568	4.38700	1.84211
N	0.87763	6.13405	4.00113
H	1.47529	5.49404	3.47787
H	1.00534	5.98001	5.00207
H	1.11835	7.09831	3.76869
C	-1.09757	4.42522	4.29856
C	-0.79623	4.08606	5.64057
C	-1.83449	3.46821	3.58665
C	-1.19280	2.87093	6.21052
C	-2.25350	2.25157	4.13580
H	-2.09542	3.68586	2.55538
C	-1.92582	1.95320	5.45343
H	-0.94472	2.62847	7.23580
H	-2.82586	1.55006	3.53640
H	-2.23328	1.01433	5.90438
C	-1.55875	7.10679	4.20929
C	-1.18515	8.46644	4.07349
C	-2.78073	6.87988	4.85816
C	-1.97505	9.51042	4.56827
C	-3.59466	7.90516	5.35144
H	-3.11178	5.85323	4.98217
C	-3.18466	9.22559	5.20737
H	-1.66741	10.54248	4.45974
H	-4.53351	7.66870	5.84298
H	-3.79355	10.04168	5.58501
O	0.00733	8.71171	3.41940
C	1.97058	3.43781	1.13737
H	1.31379	2.68730	0.67968
H	2.52963	3.94020	0.33492
C	0.43710	10.06055	3.21781
H	-0.33158	10.61127	2.65768
H	0.56717	10.54655	4.19194
O	-0.08527	5.02428	6.36490
C	0.21999	4.77102	7.73803
H	-0.71077	4.58893	8.29312
H	0.84700	3.87256	7.81323
C	2.94097	2.77049	2.11314
C	4.01315	3.75879	2.61233
H	3.52673	4.69592	2.90795
H	4.67457	4.01427	1.77214
C	4.85094	3.24863	3.79060
H	5.43357	2.35999	3.52743
H	5.55784	4.01519	4.12589
H	4.21300	2.98736	4.64339
C	3.55968	1.53777	1.43567
H	4.13153	1.82424	0.54388
H	4.24015	1.01103	2.11018
H	2.78654	0.82669	1.12551
H	2.34972	2.43202	2.97523
C	1.74749	10.04818	2.42928
H	2.44638	9.38716	2.96325
C	2.37257	11.45771	2.38464
H	1.63055	12.16751	1.99203
H	3.18632	11.43926	1.64919
C	2.92820	11.97258	3.71848
H	3.41071	12.94674	3.58528
H	2.14821	12.09792	4.47660
H	3.67883	11.28351	4.12375
C	1.53781	9.49636	1.01194
H	2.49344	9.41460	0.48272
H	1.06939	8.50861	1.02788
H	0.89031	10.16410	0.42972
C	0.95389	5.98058	8.31868
H	1.82427	6.17308	7.67322
C	1.48129	5.62233	9.72219
H	0.62750	5.43442	10.38855
H	2.03575	4.67662	9.65975
C	2.39139	6.68444	10.34952
H	1.86106	7.62428	10.53235
H	2.78432	6.33686	11.31082
H	3.24746	6.90388	9.70006
C	0.05850	7.22764	8.32165
H	-0.35474	7.41928	7.32777
H	-0.78283	7.09567	9.01399
H	0.61182	8.11813	8.63408

1d-c7

B	-0.31062	0.41325	0.17380
C	0.59572	-0.95221	0.27248
C	1.93331	-0.96062	-0.14730
C	0.09237	-2.19611	0.72594
C	2.74113	-2.10268	-0.12781
H	2.36261	-0.03103	-0.50869
C	0.88219	-3.35079	0.76670
C	2.21049	-3.30113	0.33551
H	3.77051	-2.04957	-0.46919
H	0.47931	-4.28987	1.12343

H	2.81543	-4.20250	0.36644
O	-1.22969	-2.21149	1.12800
N	-1.13546	0.53608	1.57716
H	-1.57345	-0.36040	1.79260
H	-1.85275	1.25552	1.48386
H	-0.49039	0.79003	2.32569
C	-1.42527	0.32666	-1.02767
C	-2.53984	1.19454	-1.12900
C	-1.28511	-0.59894	-2.07172
C	-3.44297	1.12697	-2.19610
C	-2.16844	-0.68187	-3.15307
H	-0.44168	-1.28198	-2.04066
C	-3.25325	0.18574	-3.21105
H	-4.29187	1.79632	-2.25088
H	-2.00594	-1.41632	-3.93611
H	-3.95738	0.14197	-4.03667
C	0.62055	1.76030	0.05509
C	1.42731	2.24858	1.11180
C	0.70753	2.47404	-1.14872
C	2.24926	3.37186	0.96521
C	1.52663	3.59442	-1.32392
H	0.11212	2.13331	-1.99034
C	2.29824	4.04437	-0.25851
H	2.85326	3.73127	1.78847
H	1.55737	4.10402	-2.28225
H	2.94091	4.91331	-0.36476
O	1.36185	1.55635	2.30715
C	-1.81355	-3.43500	1.58109
H	-1.73225	-4.19517	0.79168
H	-1.26742	-3.79507	2.46320
C	2.32199	1.83800	3.32919
H	3.32885	1.75584	2.90102
H	2.18845	2.86528	3.69810
O	-2.69541	2.12010	-0.11308
C	-3.64490	3.17789	-0.27296
H	-3.44286	3.69436	-1.21970
H	-4.66341	2.76584	-0.31714
C	-3.28208	-3.18938	1.93010
C	-3.84524	-4.43501	2.64267
H	-3.16909	-4.70700	3.46395
H	-3.83588	-5.28303	1.94332
C	-5.25896	-4.25878	3.20860
H	-5.99870	-4.08595	2.42066
H	-5.56853	-5.15351	3.75899
H	-5.30349	-3.40996	3.90166
C	-4.09385	-2.79947	0.68676
H	-4.14632	-3.63987	-0.01720
H	-5.11815	-2.51804	0.94818
H	-3.63605	-1.95518	0.16456
H	-3.30957	-2.35366	2.64566
C	2.15277	0.84162	4.47715
H	2.13822	-0.16289	4.03213
C	0.82711	1.06197	5.23134
H	0.01350	1.16149	4.50330
H	0.87858	2.02435	5.76035
C	0.46383	-0.05114	6.22117
H	-0.51065	0.14198	6.68250
H	0.40733	-1.02334	5.71705
H	1.19632	-0.13858	7.02995
C	3.37280	0.94325	5.40599
H	3.31122	0.22331	6.22651
H	4.30211	0.74410	4.86161
H	3.44890	1.94499	5.84768
C	-3.52391	4.15391	0.89844
H	-2.45986	4.41028	0.99613
C	-3.99742	3.51793	2.21986
H	-5.08126	3.34401	2.15820
H	-3.53896	2.52761	2.32493
C	-3.67845	4.33999	3.47410
H	-4.18866	5.30847	3.47265
H	-3.99216	3.80695	4.37806
H	-2.60182	4.53113	3.55655
C	-4.30203	5.43507	0.55942
H	-3.92869	5.89209	-0.36328
H	-5.36996	5.22275	0.42228
H	-4.21188	6.17937	1.35515

1d-c8

B	-0.29492	0.37217	-0.92472
C	-0.55753	1.98282	-1.10406
C	-1.50479	2.45820	-2.02223
C	0.17930	2.97973	-0.41884
C	-1.72751	3.81711	-2.26788
H	-2.09086	1.73063	-2.57545
C	-0.02818	4.34577	-0.64171
C	-0.98448	4.76309	-1.57100
H	-2.47222	4.12585	-2.99523
H	0.54435	5.09107	-0.10473
H	-1.13715	5.82501	-1.73944
O	1.12236	2.53467	0.48944
N	-0.20016	0.08123	0.67879
H	0.42619	0.76232	1.10819
H	0.16419	-0.86044	0.82867
H	-1.13017	0.15917	1.09200
C	1.14148	-0.08699	-1.57670
C	1.78636	-1.31114	-1.27290
C	1.78708	0.71287	-2.53017
C	2.99295	-1.68856	-1.87317
C	2.98771	0.35476	-3.15282
H	1.32811	1.65911	-2.80008
C	3.59257	-0.85113	-2.81759
H	3.47143	-2.62633	-1.62190
H	3.43980	1.01418	-3.88752
H	4.52681	-1.15307	-3.28171
C	-1.54375	-0.52584	-1.50024
C	-2.81072	-0.60896	-0.87192
C	-1.42179	-1.23316	-2.70474
C	-3.86216	-1.36046	-1.40923
C	-2.46044	-1.98039	-3.27014
H	-0.47002	-1.19621	-3.22605
C	-3.68411	-2.04567	-2.61393
H	-4.82001	-1.41716	-0.90848
H	-2.30786	-2.50495	-4.20849
H	-4.50657	-2.62288	-3.02595
O	-2.95887	0.09773	0.30678
C	2.05458	3.46819	1.04173
H	2.56915	3.98265	0.22035
H	1.52298	4.22225	1.63978
C	-4.21832	0.08140	0.98430
H	-5.00433	0.44536	0.30791
H	-4.46002	-0.94943	1.26906
O	1.15680	-2.12162	-0.34755
C	1.73032	-3.38815	-0.01312
H	1.82848	-3.99833	-0.92206
H	2.73133	-3.23138	0.40563
C	3.06298	2.72229	1.91684
C	2.40255	2.16719	3.19386
H	1.46502	1.66733	2.92307
H	2.11889	3.01133	3.83860
C	3.27211	1.18389	3.98591
H	4.18604	1.65398	4.36295
H	2.72619	0.79111	4.85044
H	3.56924	0.33128	3.36361
C	4.23848	3.66041	2.23222
H	3.89977	4.54121	2.79243
H	5.00011	3.15767	2.83441
H	4.72208	4.01110	1.31419
H	3.44251	1.87813	1.32450
C	-4.13018	0.98717	2.21337
H	-3.25022	0.67087	2.79306
C	-5.37301	0.81474	3.11078
H	-6.27752	0.97575	2.50700
H	-5.36300	1.62144	3.85413
C	-5.47175	-0.52818	3.84537
H	-6.34427	-0.54284	4.50724
H	-5.57152	-1.37494	3.15843
H	-4.58348	-0.70399	4.46388
C	-3.94745	2.45655	1.80744
H	-3.79786	3.08720	2.69052
H	-3.08949	2.58844	1.14282

H	-4.83742	2.82477	1.28161
C	0.81820	-4.09234	0.99223
H	0.65735	-3.39916	1.83144
C	1.49523	-5.36048	1.55161
H	0.73633	-5.92771	2.10458
H	1.79902	-6.00478	0.71429
C	2.69340	-5.11275	2.47679
H	2.41071	-4.47709	3.32442
H	3.06935	-6.05799	2.88268
H	3.52773	-4.62690	1.96041
C	-0.54300	-4.42842	0.36675
H	-1.02272	-3.54204	-0.05692
H	-0.42635	-5.16299	-0.44000
H	-1.21713	-4.85973	1.11479

1d-c9

B	-0.23462	0.42859	0.13037
C	0.67150	-0.93675	0.23690
C	2.00718	-0.94743	-0.18887
C	0.17003	-2.17857	0.69821
C	2.81481	-2.08955	-0.16859
H	2.43525	-0.01937	-0.55542
C	0.95961	-3.33339	0.73921
C	2.28588	-3.28597	0.30156
H	3.84279	-2.03787	-0.51435
H	0.55796	-4.27127	1.10043
H	2.89051	-4.18754	0.33300
O	-1.15052	-2.19246	1.10544
N	-1.06307	0.55976	1.53224
H	-1.49206	-0.33896	1.75588
H	-1.78875	1.27012	1.42948
H	-0.42163	0.82966	2.27891
C	-1.34721	0.33287	-1.07370
C	-2.47102	1.18934	-1.17470
C	-1.20087	-0.59425	-2.11519
C	-3.38027	1.10564	-2.23526
C	-2.08871	-0.69160	-3.19200
H	-0.35116	-1.26948	-2.08416
C	-3.18447	0.16202	-3.24720
H	-4.23575	1.76644	-2.29030
H	-1.92208	-1.42784	-3.97248
H	-3.89249	0.10621	-4.06878
C	0.69874	1.77348	-0.00091
C	1.48464	2.29069	1.05814
C	0.80604	2.45764	-1.21995
C	2.30097	3.41656	0.90114
C	1.62269	3.57807	-1.40685
H	0.22553	2.09582	-2.06318
C	2.36965	4.05926	-0.33781
H	2.88903	3.79871	1.72559
H	1.66798	4.06529	-2.37615
H	3.00863	4.92983	-0.45254
O	1.40601	1.61893	2.26324
C	-1.73175	-3.41516	1.56625
H	-1.66071	-4.17631	0.77658
H	-1.17328	-3.77232	2.43943
C	2.19141	2.06465	3.37185
H	3.25530	2.05465	3.09609
H	1.91047	3.09457	3.62148
O	-2.62315	2.12151	-0.16583
C	-3.71336	3.04537	-0.21748
H	-3.66299	3.62083	-1.15255
H	-4.65809	2.48919	-0.20625
C	-3.19871	-3.16364	1.91714
C	-3.80651	-4.38441	2.63826
H	-3.65375	-5.28028	2.01964
H	-4.89249	-4.23697	2.68671
C	-3.28207	-4.63813	4.05726
H	-3.43217	-3.75866	4.69484
H	-3.81243	-5.47713	4.52027
H	-2.21460	-4.88142	4.07221
C	-4.01495	-2.80294	0.66782
H	-4.05503	-3.65258	-0.02542
H	-5.04471	-2.54545	0.93859
H	-3.57942	-1.95571	0.13142
H	-3.22822	-2.31250	2.61374
C	1.95071	1.12676	4.55555
H	0.86412	1.08071	4.72248
C	2.60181	1.68486	5.83779
H	3.66664	1.87861	5.64496
H	2.57515	0.89379	6.59735
C	1.94203	2.94301	6.41628
H	2.42078	3.23135	7.35821
H	2.01285	3.80152	5.74043
H	0.87939	2.76949	6.62411
C	2.45992	-0.28996	4.25420
H	2.21229	-0.97313	5.07394
H	2.02547	-0.68751	3.33305
H	3.55094	-0.29152	4.13694
C	-3.61695	3.98949	0.98170
H	-3.55706	3.36647	1.88660
C	-4.88296	4.86359	1.09430
H	-4.68394	5.64476	1.83837
H	-5.04167	5.39054	0.14262
C	-6.16308	4.11920	1.49453
H	-6.02955	3.59058	2.44599
H	-6.99438	4.82123	1.61970
H	-6.47077	3.38256	0.74519
C	-2.35512	4.86051	0.90289
H	-1.45181	4.25370	0.79735
H	-2.40598	5.53691	0.04033
H	-2.25259	5.47620	1.80312

1d-c10

B	-0.02302	0.31510	-0.31987
C	0.64984	-1.18168	-0.26969
C	0.62239	-2.02507	-1.38928
C	1.34145	-1.68841	0.85776
C	1.22886	-3.28529	-1.42100
H	0.10910	-1.67681	-2.28053
C	1.95084	-2.94833	0.85442
C	1.89403	-3.74679	-0.29081
H	1.17972	-3.89271	-2.31969
H	2.47038	-3.31838	1.72896
H	2.37202	-4.72193	-0.28585
O	1.38435	-0.87109	1.97267
N	-0.88068	0.48534	1.05819
H	-1.71712	-0.09641	1.00726
H	-1.14590	1.46410	1.17490
H	-0.30636	0.19176	1.84876
C	-1.08319	0.47687	-1.56244
C	-2.33109	-0.19066	-1.61936
C	-0.76726	1.26010	-2.68204
C	-3.19188	-0.07077	-2.71640
C	-1.60596	1.39062	-3.79385
H	0.18196	1.78718	-2.68703
C	-2.82480	0.72207	-3.80667
H	-4.14407	-0.58522	-2.73619
H	-1.30543	2.00767	-4.63523
H	-3.49761	0.80780	-4.65482
C	1.11837	1.49549	-0.32642
C	0.86019	2.84631	0.01226
C	2.43146	1.21667	-0.73163
C	1.85380	3.83091	-0.03698
C	3.43949	2.18401	-0.80235
H	2.67614	0.19532	-1.00720
C	3.14654	3.49600	-0.44824
H	1.63723	4.85609	0.23409
H	4.43838	1.90933	-1.12757
H	3.91031	4.26702	-0.48786
O	-0.43371	3.14721	0.39532
C	2.25730	-1.21272	3.05321
H	1.93390	-2.15810	3.51208
H	3.27132	-1.35161	2.65773
C	-0.77095	4.49294	0.73899
H	-0.16101	4.81486	1.59355
H	-0.55043	5.15599	-0.10925
O	-2.66197	-0.97310	-0.52804
C	-3.77552	-1.86613	-0.62428
H	-4.70723	-1.29317	-0.73619

H	-3.64869	-2.49373	-1.51532
C	2.24271	-0.09167	4.09332
C	3.39891	-0.31699	5.08863
H	4.32705	-0.45665	4.51968
H	3.22739	-1.25656	5.63328
C	3.60658	0.82253	6.09286
H	2.75154	0.94045	6.76606
H	4.48841	0.63238	6.71375
H	3.76202	1.77791	5.57762
C	0.88143	0.00904	4.79639
H	0.68339	-0.89389	5.38808
H	0.84007	0.86825	5.47184
H	0.06350	0.12042	4.07858
H	2.43509	0.84786	3.55683
C	-2.25697	4.56614	1.09339
H	-2.44623	3.80268	1.86296
C	-3.14371	4.25431	-0.12723
H	-2.76816	3.34684	-0.61136
H	-3.02714	5.06473	-0.86098
C	-4.63020	4.06684	0.19789
H	-5.19080	3.79335	-0.70224
H	-4.77530	3.26639	0.93383
H	-5.08495	4.97684	0.60315
C	-2.55557	5.94424	1.70356
H	-3.60009	6.02368	2.01660
H	-1.93169	6.13381	2.58406
H	-2.36460	6.74435	0.97738
C	-3.84051	-2.73595	0.63180
H	-2.84257	-3.17402	0.77183
C	-4.83699	-3.88803	0.39151
H	-5.84296	-3.46958	0.24412
H	-4.57175	-4.38840	-0.54869
C	-4.88093	-4.93673	1.50886
H	-5.24463	-4.51900	2.45301
H	-5.54851	-5.76110	1.23673
H	-3.88599	-5.36030	1.69073
C	-4.19615	-1.90787	1.87489
H	-3.51072	-1.06568	2.00777
H	-5.21167	-1.50002	1.79137
H	-4.15057	-2.51062	2.78636
